# Nuclear pore protein POM121 regulates subcellular localization and transcriptional activity of PPARγ

**DOI:** 10.1038/s41419-023-06371-1

**Published:** 2024-01-04

**Authors:** Yanxiong Yu, Mohammad S. Farooq, Sabine Eberhart Meessen, Yidan Jiang, Dominik Kato, Tianzuo Zhan, Christel Weiss, Rony Seger, Wei Kang, Xiang Zhang, Jun Yu, Matthias P. A. Ebert, Elke Burgermeister

**Affiliations:** 1 Department of Medicine II, University Medical Center Mannheim, Medical Faculty Mannheim, Heidelberg University, Mannheim, Germany; 2 Department of Surgery, Hospital of the University of Pennsylvania, Philadelphia, PA USA; 3 Department of Medical Statistics and Biomathematics, Medical Faculty Mannheim, Heidelberg University, Mannheim, Germany; 4 Department of Immunology and Regenerative Biology, Weizmann Institute of Science, Rehovot, Israel; 5 Department of Anatomical and Cellular Pathology, State Key Laboratory of Digestive Disease, Li Ka Shing Institute of Health Sciences, The Chinese University of Hong Kong, Hong Kong, China; 6 Institute of Digestive Disease and Department of Medicine and Therapeutics, State Key Laboratory of Digestive Disease, Li Ka Shing Institute of Health Sciences, The Chinese University of Hong Kong, Hong Kong, China; 7 DKFZ-Hector Institute, Medical Faculty Mannheim, Heidelberg University, Mannheim, Germany; 8 Mannheim Institute for Innate Immunoscience (MI3), Medical Faculty Mannheim, Heidelberg University, Mannheim, Germany; 9 Clinical Cooperation Unit Healthy Metabolism, Center of Preventive Medicine and Digital Health, Medical Faculty Mannheim, Heidelberg University, Mannheim, Germany; 10 Mannheim Cancer Center (MCC), Medical Faculty Mannheim, Heidelberg University, Mannheim, Germany

**Keywords:** Nuclear pore complex, Colorectal cancer

## Abstract

Manipulation of the subcellular localization of transcription factors by preventing their shuttling via the nuclear pore complex (NPC) emerges as a novel therapeutic strategy against cancer. One transmembrane component of the NPC is POM121, encoded by a tandem gene locus *POM121A/C* on chromosome 7. Overexpression of POM121 is associated with metabolic diseases (e.g., diabetes) and unfavorable clinical outcome in patients with colorectal cancer (CRC). Peroxisome proliferator-activated receptor-gamma (PPARγ) is a transcription factor with anti-diabetic and anti-tumoral efficacy. It is inhibited by export from the nucleus to the cytosol via the RAS-RAF-MEK1/2-ERK1/2 signaling pathway, a major oncogenic driver of CRC. We therefore hypothesized that POM121 participates in the transport of PPARγ across the NPC to regulate its transcriptional activity on genes involved in metabolic and tumor control. We found that *POM121A/C* mRNA was enriched and POM121 protein co-expressed with PPARγ in tissues from CRC patients conferring poor prognosis. Its interactome was predicted to include proteins responsible for tumor metabolism and immunity, and *in-silico* modeling provided insights into potential 3D structures of POM121. A peptide region downstream of the nuclear localization sequence (NLS) of POM121 was identified as a cytoplasmic interactor of PPARγ. POM121 positivity correlated with the cytoplasmic localization of PPARγ in patients with *KRAS* mutant CRC. In contrast, *POM121A/C* silencing by CRISPR/Cas9 sgRNA or siRNA enforced nuclear accumulation of PPARγ and activated PPARγ target genes promoting lipid metabolism and cell cycle arrest resulting in reduced proliferation of human CRC cells. Our data suggest the POM121-PPARγ axis as a potential drugable target in CRC.

## Introduction

Colorectal cancer (CRC) is one of the most frequently diagnosed and deadly malignancy worldwide [1]. The success of targeted or immunotherapies is limited to small subsets of patients with defined consensus molecular subtypes (CMS) [2], thus, calling for broader intervention strategies. Spatio-temporal compartmentalization of transcription factors is an emerging theme in cancer therapy. As such, subcellular transport of nuclear-factor-kappa-B (NFκB) [3], MYC, E2F1 and androgen receptor [4] has been identified as potential target both in vitro and in vivo.

Nuclear pore complexes (NPC) represent multi-protein supra-molecular assemblies (>120 MDa) which form hydrophilic channels that facilitate the energy-dependent and passive transport across the nuclear envelope [5]. The RAN GTPase cycle fosters the reciprocal import and export of cargo proteins, with importins and exportins (e.g., CRM1) as cargo-acceptors which piggy-back cargo across the nuclear pores. The NPC is composed of about 100 distinct nucleoproteins (Nups), and three of them are integral transmembrane proteins essential to assemble and anchor the NPC to the nuclear envelope and the endoplasmic reticulum (ER) at the exit of mitosis and during the interphase of the cell cycle [6]. Among the three, POM121 has been shown to be indispensable for NPC stability and function in both processes [7, 8]. These genes encoding this protein appear on a fragile locus in human chromosome (chr) 7q11.23, a region associated with Williams Beuren syndrome, an inherited developmental disease [9]. This region was subjected to gene duplications during hominid evolution [10] and encodes for all three POM121 isoforms, *POM121A* and *POM121C* and a pseudogene *POM121B* [11].

POM121 is a 121 kDa type-1 transmembrane (TM) protein that comprises a single-pass N-terminal membrane-spanning domain, an internal nuclear localization sequence (NLS) which interacts with importin-α/β, and a C-terminal cytoplasmic/nucleoplasmic domain with phenylalanine-glycine (FG) repeats which aid cargo transfer [12]. N- or C-terminally truncated variants of POM121 include TM- and/or NLS-deficient soluble forms [13, 14], which act as chaperones in the cytoplasm and coregulators of the chromatin and gene expression by binding to transcription factors [15]. POM121 isoforms are implicated in neurodegenerative diseases [16], inflammation [17], virus replication [14, 18] and regulation of cell viability [19, 20].

Due to the association between chromosomal instability (CIN), nuclear structural aberrations and tumor progression, POM121 and other Nups have been linked to cancer. As such, POM121 promoted lethal prostate cancer through binding to importin-β, leading to nuclear import of oncogenic transcription factors (E2F1, MYC e.a.) [4]. Importazole, a small molecule inhibitor with selectivity to RAN-importin-β, as well as importin-β knockout (KO) decreased tumor cell proliferation and prolonged survival in mice. In patients, POM121 is overexpressed in cancers of the upper gastrointestinal tract [21–23], the lungs [24] and in CRC [25], and therein associated with higher tumor (TNM) staging, metastasis [26] and poor prognosis, while POM121 gene fusions present in leukemia [27]. POM121 has been linked to metabolic disease (e.g., fasting serum insulin) [28] and lipid/carbohydrate metabolism [22, 29] in cancers by contributing to adipogenesis in human white fat tissues. POM121 is also essential for nuclear envelope herniations (blebs) through interaction with torsin ATPases [30], regulators of lipid energy metabolism and membrane phospholipid turn-over, suggesting a functional crosstalk between POM121 and metabolic transcription factors.

Peroxisome-proliferator-activated-receptors (PPARs: α, β/δ, γ (31)) belong to the nuclear “hormone” receptor superfamily. Beyond its function as insulin-sensitizer in diabetics, PPARγ promotes cell and tissue differentiation and host immunity [31]. Lipid mediators from diets or inflammation activate PPARγ as do prescription-approved drug agonists (glitazones) [32]. Targeting PPARγ has been proven to be efficacious against leukemia [33–35] and in preclinical settings [31].

RAS-RAF-MEK1/2-ERK1/2 signaling is a major driver of CRC and inhibits transcription by PPARs via their export from the nucleus to the cytoplasm [36–38]. However, the role of the NPC in this transport is unknown. Since the latter may be one possible mechanism causing failure of PPARγ agonists in therapy of solid tumors, we hypothesized that POM121 regulates the subcellular localization of PPARγ in CRC cells. Our data show that POM121 (i) interacts with PPARγ, (ii) prevents its accumulation in the nucleus and (iii) down-regulates transcription from PPARγ target genes. Thus, POM121 may contribute to the progression of CRC by disrupting the balance of pro- *vs*. anti-oncogenic transcription factors.

## Materials and methods

### Reagents

Chemicals were obtained from Merck (Darmstadt, Germany) if not stated otherwise. Antibodies (Abs) are listed in Table S1. Rosiglitazone (rosi) was purchased from Cayman (Ann Arbor, MI). Control siRNA and siRNA against human *POM121A/C* transcripts were from Ambion Silencer® Select (s59624) (Thermofisher Scientific, Waltham, MA).

### Subjects

Tissue microarrays (TMAs) were generated from formalin-fixed and paraffin-embedded (FFPE) specimens of patients with CRC (*n* = 408 cases) [39]. Written informed consent was provided by all patients. The study followed the principles of the Declaration of Helsinki and was approved by the Medical Ethics Committees of the participating Universities. The clinical and pathological characteristics of the cohorts were provided previously [39].

### Cell lines

Human embryonic kidney cells immortalized by large T antigen of Simian Virus 40 (HEK293T) and CRC cell lines (HT29, SW480, HCT116) were from the American Type Culture Collection (ATCC, Manassas, VA). All lines were mycoplasma-free and cultivated in complete Dulbecco’s Modified Eagle’s Medium (DMEM) according to the guidelines of the distributor. Basal media, here from defined as “complete” media, were supplemented with 10% (*v/v*) fetal calf serum (FCS), 2 mM L-glutamine and 100 U/ml penicillin/streptomycin (all from Thermofisher).

### CRISPR/Cas9 sgRNA

The sgRNA sequences were designed using the E-CRISP target site identification tool from the German Cancer Research Center (DKFZ) corresponding to the (98% identical) human genomic sequences of *POM121A* transcript variant 1 (NM_001257190.3) and *POM121C* transcript variant 1 (NM_001099415.3) (Table S2). Custom oligonucleotides for POM121 sgRNA were obtained from Eurofins (Heidelberg, Germany), annealed and inserted into the pSpCas9(BB)-2A-Puro(PX459) vector (#62988, Addgene, LGC Standards Teddington, UK) utilizing the NEB Golden Gate Assembly Kit (New England Biolabs GmbH, Frankfurt, Germany). Oligonucleotide annealing was accomplished using a thermal cycler with the following conditions: 37 °C for 30 min, 95 °C for 5 min, and decrease to 25 °C at a rate of 5 °C per min. The Golden Gate reaction was carried out under the following conditions: 37 °C for 5 min and 20 °C for 5 min repeated for 15 cycles. Transformation of the ligated sgRNA/Cas9 plasmid was performed using One Shot^TM^ TOP10 *E. coli* chemically competent cells (Thermofisher). Cells were processed according to the manufacturer’s recommendations. Afterwards, cells were spread on LB agar plates and incubated overnight at 37 °C. Plasmid DNA was harvested from colonies using the Qiagen HiSpeed Plasmid Midi Kit (Qiagen, Hilden, Germany).

### Cell transfection

Cells (4 × 10^6^) were treated with a mixture of Turbofect^TM^ transfection reagent (Thermofisher) and plasmid DNA (2 µg/ml) according to the manufacturer protocol. Cells transiently transfected with plasmids or siRNAs were analyzed after 24–72 h. For generation of stable clones, selection was performed using puromycin dihydrochloride at a concentration of 1–10 μg/ml. In brief, transfected cells were seeded in 6-well plates and, after 4–7 days, surviving single cell clones were diluted into individual wells of 96-well plates and screened for *POM121* mRNA/protein knockout (KO) by RT-qPCR and Western blot using N- and C-terminal primers (Table S2) and Abs (Table S1). Two clones with a maximal difference (Delta (Δ)) of high *vs*. low POM121 full-length (FL) protein expression were selected for further experiments.

### Viability assay (MTT)

Colorimetric cell viability assay based on 3-(4,5-dimethylthiazol-2-yl)-2,5-diphenyl tetrazolium bromide (MTT) was conducted according to the manufacturer’s protocol (Roche Diagnostics, Mannheim, Germany).

### Immunofluorescence microscopy (IF)

In situ staining was done after fixation. In brief, cells were seeded in 8-well chamber slides, followed by fixation in 4% (*v/v*) formaldehyde buffered in PBS for 30 min at room temperature. Cells were permeabilized by 0.5% (*v/v)* Triton X-100 in PBS for 30 min. After 1 h blocking with 100% (*v/v*) FCS, primary Ab [in 0.5% Triton X-100, 10% FCS/PBS, all (*v/v*)] was added overnight at 4 °C, followed by an 1 h incubation with secondary Ab (in 10% FCS/PBS (*v/v)*) and 4’,6-diamidino-2-phenylindol (DAPI, 50 ng/ml) for 10 min in the dark at room temperature. Mounting medium (Dako, Hamburg, Germany) was used to cover the slide, and images were acquired in triple color mode using AXIO Observer.Z1-ApoTome.2. fluorescence microscope and ZEN software (Zeiss, Jena, Germany). Fluorescence signals (*n* > 50 cells/nuclei per field, *n* ≥ 3 fields per image) from Abs, phalloidin and DAPI dyes were manually counted with Image J (imagej.nih.gov/ij).

### Co-immunoprecipitation (CoIP) and mass spectrometry (MS)

Briefly, cytosolic lysates were prepared from cells using detergent-free hypotonic lysis buffer (20 mM Tris-HCl pH 7.4, 2 mM EGTA, 2 mM MgCl_2_, protease inhibitor tablet (cOmplete®, Roche), 5 mM Na_3_VO_4_, 1 mM DTT) and subjected to immunoprecipitation (IP), followed by SDS-PAGE and detection of the precipitated proteins by Western (immuno) blot (IB). For mass spectrometry (MS), immunoprecipitates were separated by SDS-PAGE and detected by silver staining as detailed previously [40]. Bands were cut from gels and sequenced (Table S3) using GPS Explorer 2 software (Applied Biosystems/Thermofisher) in cooperation with the Dept. of Proteomics and Bioanalytics (Technische Universität München, Munich, Germany) [41]. Subcellular fractionation (SCF) followed a previous protocol [42].

### Western blot

Methods were conducted as described previously [42]. Abs are listed in Table S1. Membranes were stained for 1 min by chemiluminescence staining protocol with solution A (5 ml 0.1 M Tris-HCl pH 8.5 + 3 µl 30% (*v/v*) H_2_O_2_) and solution B (5 ml 0.1 M Tris-HCl pH 8.5 + 50 µl 250 mM luminol + 22 µl 90 mM p-coumaric acid). Membranes were imaged using the Fusion Solo S CCD imaging system (Vilber Lourmat Deutschland GmbH, Germany).

### Nucleic acid isolation, reverse transcription (RT) and quantitative PCR (qPCR)

Samples were processed and methods performed on total RNA as published [43]. PCR primers are listed in Table S2. Gel imaging was conducted using the Gel Jet Imager (Intas Science Imaging, Germany) and analyzed using the LabImage software.

### Immunohistochemistry (IHC)

IHC was conducted on FFPE TMA samples [39] using Ventana NexES automated Stainer (Ventana, Roche). Primary Abs were against PPARγ(C) (#95128, Cell Signaling) and POM121(N) (#PA5–85161, Thermofisher) and both diluted 1:100 (Table S1). The immunoreactive score was represented by its percentage of positive cells and the intensity of the stain in epithelial and lamina propria (stroma) cells: 0 + = negative (0–25%), 1 + = weak (25–50%), 2 + = moderate (50–75%), 3 + = strong (75–100%). Signals were quantified observer-blinded at a standard bright-field microscope using Image J (imagej.nih.gov/ij) (*n* > 20 signals per field; *n* = 5 fields per image). The field area was defined by the morphology (e.g., crypt-villus unit) as indicated in the legends to figures. For dichotome analysis, staining scores were grouped as negative (low expression = *scores* 0/1) *vs*. positive (high expression = *scores* 2/3). Staining scores and clinical data were analyzed by SAS statistical package version 9.4 (SAS Institute, Cary, NC).

### Statistics

Results are displayed as means ± S.E. from independent experiments, herewith defined as replicates, from different cell passages or individuals (patients). Optical densities (OD) of bands in gels from Western blots and PCRs were measured using automated imaging devices and quantified with Image J (imagej.nih.gov/ij). Data were normalized to house-keeping genes or proteins as indicated in the legends to figures and calculated as -fold or % compared to control. Statistical analysis was done with Graphpad Prism (version 4.0, La Jolla, CA). Therein, data were first tested for non- *vs*. parametric distribution, followed by the appropriate statistical procedures with Bonferroni post-tests for 2way-ANOVA and Tukey or Dunn post-tests for 1way-ANOVA or Kruskal Wallis test, respectively. The 2group comparisons were done with Mann Whitney/Wilcoxon tests for non-parametric or *t*-test for parametric data. All tests were unpaired and two-sided assuming equal variance between groups. *P*-values < 0.05 were considered significant and marked by asterisk (*). Trends (*p* < = 0.1) are indicated by exact *p*-values. Non-significance (n.s.) was annotated in the legend.

### Bioinformatics

Search queries for were conducted separately for human POM121 (*POM121* *A*) and POM121C (*POM121C*) genes/proteins. Protein-protein interaction (PPI) networks were generated and visualized with UniProt (https://www.uniprot.org) programs: STRING [44] (https://string-db.org), IntAct [45] (http://www.ebi.ac.uk/intact) and BioGRID (https://thebiogrid.org) [46]. Genomic alterations were inquired from TCGA datasets using cBioPortal (https://www.cbioportal.org) [47], and copy number variations (CNV) from GISTIC (https://software.broadinstitute.org/ cancer/cga/gistic). Oncomine [48] and OncoDB [49] (http://oncodb.org) were analyzed for mRNA expression in cancer *vs*. normal patient tissues. Secondary structure and hydrophathy prediction were done with EMBOSS explorer (https://www.bioinformatics.nl/cgi-bin/emboss/) and Jpred [50] (http://www.compbio.dundee.ac.uk/jpred). For 3D modeling, primary amino acid sequences were submitted to I-TASSER server (https://zhanggroup.org/I-TASSER/) [51]. Predicted top hit models were then assessed in the Protein Data Bank (PDB) (https://www.rcsb.org) and visualized with UCSF Chimera (https://www.cgl.ucsf.edu), PyMOL 3D (https://pymol.org) or EzMOl 2.1 (http://www.sbg.bio.ic.ac.uk/ezmol/). Phyre2 [52] was employed to model protein/peptide subdomains (http://www.sbg.bio.ic.ac.uk/phyre2). SWISS-MODEL (alpha-fold DB) (https://swissmodel.expasy.org/repository/uniprot) was queried for homologous 3D structures. Nucleic and amino acid sequence alignments were done with NCBI blastn/p (https://blast.ncbi.nlm.nih.gov).

## Results

### Identification of POM121 as interactor of PPARγ

**Fig. 1:**
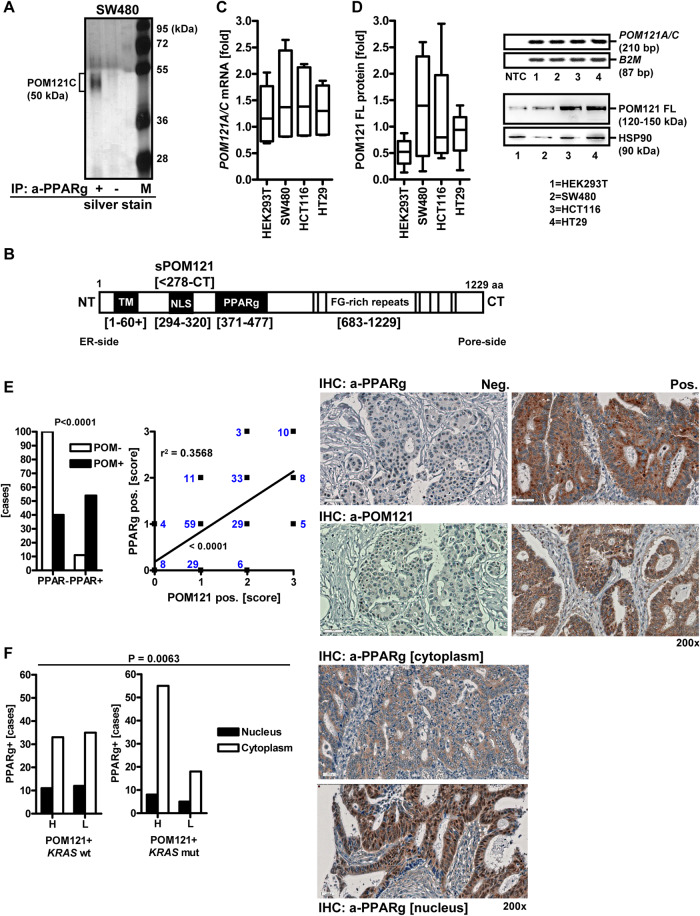
Identification of POM121 as interaction partner of PPARγ. **A** Pull-down of peptides by PPARγ Ab. CoIP was performed on cytosolic lysates of SW480 cells using PPARγ Ab or IgG (bead control) followed by detection of precipitated bands by silver staining. MS sequencing of the ~50 kDa band precipitated by PPARγ Ab contained peptides from the internal NLS region (Table S3) of POM121C (Uniprot ID: C9JFL1, A8CG34, P121C_HUMAN). M = protein marker (kDa). **B** Scheme of POM121 protein isoforms. Legend: aa = amino acid; ER = endoplasmic reticulum, TM = transmembrane (domain); sPOM121 = soluble POM121 (truncation variant); NLS = nuclear localization sequence (K + /R + ); PPARγ (binding site) = POM121 peptides precipitated by PPARγ Ab and identified by MS in A; FG = phenylalanine and glycine-rich (domain), NT = N-terminus; CT = C-terminus. NCBI BlastP alignment of *POM121A* and *POM121C* gene products (Uniprot IDs: P121A_HUMAN *vs*. P121C_HUMAN) is presented in **S1**. **C**, **D** *POM121A/C* mRNA and protein expression. HEK293T non-cancer control and human CRC cell lines (HT29, HCT116, SW480) were cultivated until subconfluency followed by RNA or protein extraction. Quantitative analyses (left) and representative images (right) from Western blots or ethidium bromide-stained agarose gels visualizing bands for a common amplification product shared by *POM121A/C* cDNAs (Table S2). **C** RT-qPCRs. Ct-values normalized to *B2M* are -fold ± S.E. **D** Western blots. O.D. values of bands normalized to HSP90 are -fold ± S.E. (n.s., Kruskal-Wallis test with Dunn post-tests, *n* = 3 per cell line and method). **E** In situ expression of POM121 and PPARγ proteins in CRC tissues. FFPE sections from patients’ TMAs were stained with Abs by immunohistochemistry (IHC). Left: Dichotome analysis grouped by negative (*scores 0/1*) *vs*. positive (*scores 2/3*) staining for each protein in both tumor/epithelial and stroma cells (*p < 0.0001, test of symmetry and Mc Nemar test, *n* = 205 cases) (Tables S5, 6). Middle: Correlation plot of graded scores for each protein (linear regression, r^2^ = 0.3658, non-zero slope = *p < 0.0001, *n* = 154 cases). Data points represent cumulative overlays of patients with the same numerical score as depicted in the graph. Right: Representative pictures of tumor tissues with pos. *vs*. neg. staining; scale bar = 50 µm; original magnifications 200x. **F** Subcellular distribution of PPARγ correlates with POM121 positivity and the *KRAS* gene mutation status of CRC tissues. Patients were stratified into *KRAS* mutant (mut) *vs*. wildtype (wt) cases and grouped by high (Abbrev. “H”, *scores 2/3*) *vs*. low (Abbrev. “L”, *scores 0/1*) POM121 protein expression (Tables S5, 6). Left: Data were analyzed as in E (*p < 0.05, Fisher Exact test, *n* = 208). Right: Representative pictures of tumor tissues with cytoplasmic *vs*. nuclear staining; scale bar = 50 µm; original magnifications 200x.

To identify novel regulators of PPARγ, co-immunoprecipitation (CoIP) experiments were performed in cytoplasmic lysates from the aneuploid CIN+ human CRC cell line SW480 [53] followed by silver staining and sequencing by mass spectrometry (MS) [41] (Fig. 1A). This cell line is suitable to detect extra-nuclear interactors for PPARs because it harbors the constitutively active *KRASG12V* mutant which triggers cytosolic retention of PPARs mediated by the downstream RAF-MEK1/2-ERK1/2 pathway [36–38]. Peptides within a band of ~50 kDa were precipitated by PPARγ antibody (Ab) and corresponded to an internal region (aa 371–477) of the human POM121 C protein, C-terminal adjacent to its NLS (aa 294–320) (Fig. 1B) (Table S3). This region is >98% (105 of 107 aa) identical between POM121 [UniProt ID: Q96HA1 (P121A_HUMAN)] and POM121C [UniProt ID: A8CG34 (P121C_HUMAN)] (**S1**) and encoded by the tandem gene locus *POM121A/C* (**S2**), whereas *POM121B* is a pseudogene [11] and was not studied further. Moreover, keratin type I cytoskeletal 10 (K1C10_HUMAN) and cytoplasmic IQ domain-containing protein G / dynein regulatory complex subunit 9 (Q9H5C8_HUMAN) were identified (Table S3).

To assess mRNA and protein expression of the two *POM121(A/C*) genes, a set of human CRC cell lines (CIN + MSS + : *BRAFV600E* HT29, *KRASG12V* SW480; CIN^-^ MSI + : *KRASG13D* HCT116 [53]) and HEK293T cells (non-cancer *KRAS* wt control) were subjected to RT-qPCR (Fig. 1C) and Western blot (Fig. 1D) using primers and Abs directed against the N- or the C-terminus of full-length (FL) *POM121A/C*. Quantitative analyses from gel imaging revealed the presence of FL mRNA and protein (120–150 kDa) [13] in all cell lines tested. Both Abs recognized the FL protein and smaller sized variants (55–75 kDa) (Table S4).

For detection of in situ expression of POM121 and PPARγ proteins, FFPE tissues from CRC patients (*n* = 205 cases) were stained with Abs against POM121 or PPARγ by immunohistochemistry (IHC) (Fig. 1E). Dichotome analysis grouped by negative (*scores 0/1*) *vs*. positive (*scores 2/3*) staining for each protein in the same sample evinced co-expression of POM121 and PPARγ in a subset of patients (*p < 0.0001, test of symmetry and Mc Nemar test; linear regression, r^2^ = 0.3658, non-0 slope = *p* < 0.0001) (Tables S5, 6).

Activating *KRAS* and *BRAF* gene mutations are frequent in CRC, and the downstream MEK1/2-ERK1/2 pathway mediates cytoplasmic retention and inactivation of PPARγ [36]. To explore if the subcellular distribution of PPARγ correlates with POM121 expression, patient cases were stratified by their *KRAS* gene mutation status (Tables S5, 6). Notably, *KRAS* mutant cases with high POM121 protein positivity had more PPARγ protein localized to the peri-nuclear and cytoplasmic regions of the tumor cells compared with *KRAS* wt cases, who showed nuclear positivity of PPARγ (*n* = 208, *p < 0.05, Fisher Exact test) (Fig. 1F). Thus, POM121 seems to inhibit nuclear accumulation of PPARγ in presence of active RAS signaling.

**Fig. 2:**
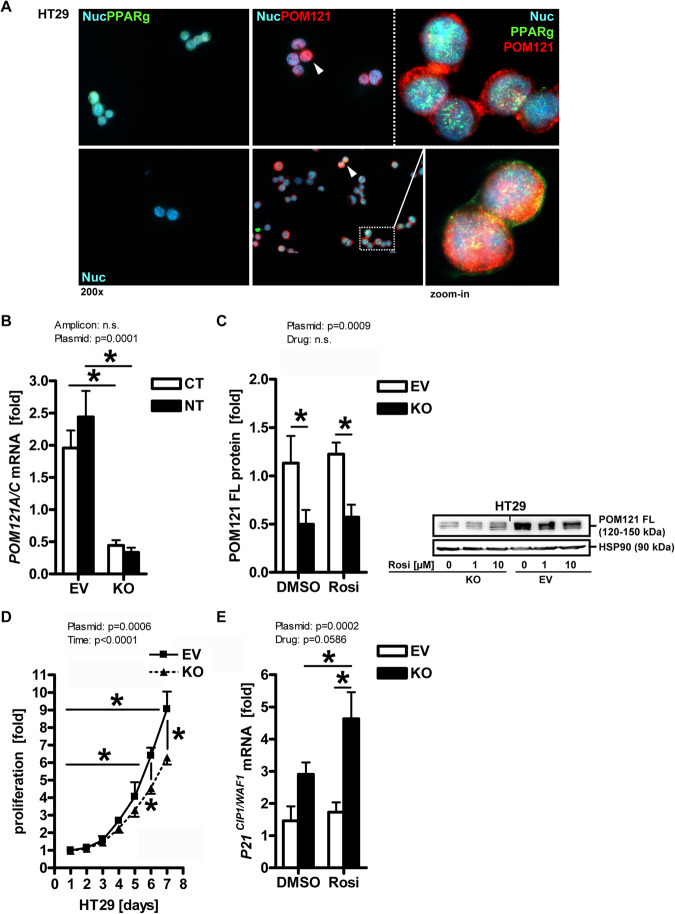
POM121 knockout reduces proliferation of human CRC cells. **A** Subcellular localization of POM121 and PPARγ proteins. Subconfluent HT29 cells were subjected to immunofluorescence (IF) microscopy. Representative images are shown. Color code: yellow = POM121/PPARγ (overlay); red = POM121; green = PPARγ; blue = nuclei (DAPI); scale bar = 50 µm; original magnifications 200x (zoomed-in 630x). Note the perinuclear distribution of POM121 and partial colocalization with PPARγ as marked by white arrows. **B** POM121 knockout (KO) reduces *POM121A/C* mRNA. HT29 cells were subjected to stable transfection with *POM121A/C* targeting CRISPR/Cas9 sgRNA plasmid (KO) or empty vector (EV), followed by clonal selection and RNA extraction. Ct-values from RT-qPCRs using primers against NT or CT amplicons normalized to *B2M* are -fold ± S.E. (*p < 0.05 *vs.* EV, 2way-ANOVA with Bonferroni post-test, *n* = 3 per clone). **C** POM121 KO reduces POM121 protein. Clonal cells were treated with vehicle (DMSO) or rosi (1–10 µM) for 48 h and then subjected to extraction of total cell lysate (TCL). Representative images (right) and quantitative analyses (left) from Western blots using Abs against the NT or CT domains. O.D. values of bands in gels normalized to HSP90 are -fold ± S.E. (*p < 0.05 *vs*. EV, 2way-ANOVA with Bonferroni post-test, *n* = 3 per clone). **D** POM121 KO reduces cell proliferation. Viability of clonal cells from B was measured by colorimetric MTT assay. O.D. values were calculated as -fold ± S.E. compared with day 0 (*p < 0.05 *vs*. EV or day, 2way-ANOVA with Bonferroni post-tests, *n* = 3 per clone). **E** POM121 KO increases basal- and ligand-mediated mRNA expression of cyclin-dependent kinase inhibitor *P21* ^*CIP1/WAF1*^. Clonal cells were treated with vehicle (DMSO) or rosi (1–10 µM) for 48 h, followed by RNA extraction. Ct-values from RT-qPCRs normalized to *B2M* are -fold ± S.E. (*p < 0.05 *vs*. vehicle or EV, 2way-ANOVA with Bonferroni post-tests, n = 3 per clone).

To recapitulate these findings on co-expression in vitro, immunofluorescence (IF) microscopy (Fig. 2A) was performed in the *BRAF* mutant HT29 cell line, the kinase bridging RAS to the MEK1/2-ERK1/2 effector module. Cells were subjected to imaging, and partial colocalization of POM121 and PPARγ proteins was visualized in the perinuclear region overlapping with the nuclear envelope, despite a strong nuclear signal for PPARγ. Collectively, these data indicated that soluble isoforms of full-length (FL) POM121 bind PPARγ and may influence its subcellular distribution.

To further dissect the protein-protein interaction (PPI) networks centered around the POM121 proteins, queries were conducted using UniProt programs (STRING [44] / IntAct [45] / BioGRID [46]) (Table S7). The initial query resulted in >100 interactor proteins with considerable overlap between POM121 (*POM121A*) and POM121C (**S3**). A significant portion of the closest interactions generated by the network involved other nucleoporins and proteins linked to NPC assembly. Proteins related to the cell cycle and cancer, e.g., breast cancer (estrogen receptor, BRCA1) and adenomatous polyposis coli (APC), a known oncogenic driver gene in CRC, were also identified. Others included HLA molecules, TRAF2/3, NFκBIA and cytokine receptors, all implicated in immune defense. Overall, this PPI network analysis suggested that POM121 is connected to cell functions apart from NPC formation, such as transcriptional regulation (e.g., of nuclear “hormone” receptors) and the cancer immuno-microenvironment.

### Correlation of *POM121A/C* with clinical characteristics in CRC patients

To explore mRNA expression of *POM121A*/C genes in publicly available clinical cohorts, we resorted to Oncomine® [48] with the filter: “*Differential Analysis: Cancer vs. Normal*” [threshold = *p < 0.0001, -fold change = >2; gene rank = top 10%]. *POM121A/C* was significantly overexpressed in two patients’ datasets [TCGA Colorectal (*n* = 237); Hong Colorectal (*n* = 82)] with the subcategories: cecum, colon, rectosigmoid and rectal adenocarcinoma (Table S8).

Similar results were obtained from interrogating the OncoDB® [49] with the data sets “colorectal adenocarcinoma” (COAD, *n* = 349) and “rectal adenocarcinoma” (READ, *n* = 104) (**S4**) (Table S9).

Then, cBioPortal® [47] database was queried for *POM121A/C* mRNA and DNA data along with associative clinical factors (**S5a**). In the dataset [Colorectal Adenocarcinoma, TCGA, PanCancer Atlas (*n* = 594)], *POM121A/C* were found to be altered in 17% and 14% of samples, respectively, comprising mainly increased mRNA expression according to the default definition (>2 and <−2 z-scores relative to diploid samples). Similar results were collected from other data sets (Table S10). Data from the pan-cancer cohorts (*n* = 10 studies; *n* = 76,639 cases) gave a trend of unfavorable prognosis in patients with *POM121A*/C gene alterations beyond mRNA as a classifier **(S5b)** (Table S11).

To further investigate correlative associations between *POM121A* (**S6)** and *POM121C* (**S7)** mRNA and clinical outcomes, an advanced Kaplan-Meier analysis (Table S11) was conducted. We selected subsets of patients with “high” *vs*. “low” mRNA, as defined as top 10% and bottom 10% of mRNA expression levels, represented as z-score >1.28 and <−1.28 when compared to diploid samples from a reference population. Therein, 28% of CRC samples fell into the two categories. Survival curves based on this subset of samples showed that patients with high mRNA expression had worse overall (OS), progression-free (PFS) and disease-specific (DSS) survival.

Stratification of patients according to the CMS [2] system revealed that both *POM121A/C* mRNA expression and copy number alterations (CNA) were enriched in the CMS2 CIN+ subtype of CRC, consistent with its role as a safeguard of chromosome segregation during mitosis (**S8)**. Head-to-head comparison of overall *POM121A vs. POM121C* gene alterations revealed 53 % (61 of 116) overlap in the same patient, indicating that changes in both genes within the chr7 locus translate into modified POM121 protein functions in CRC.

Finally, we aligned mutations in *POM121A* (**S9)** and *POM121C* (**S10)** genes as given by GISTIC [54]. This query collected supportive evidence for POM121 mRNA/protein variants due to alternative splicing and/or translation (as depicted in Table S12) in large series of cancer cell lines (e.g., CCLE [55]) and CRC patients (*n* = 13 studies). Soluble isoforms with N-terminal truncation of the TM domain or C-terminal fragments with deletions including the NLS were annotated, both of which may function as nucleo-cytoplasmatic shuttles or chromatin/transcriptional regulators independently of the NPC.

Conclusively, *POM121A/C* mRNA expression was higher in CRC compared with normal tissue, confirming reports from others [25] that POM121 overexpression positively correlates with CRC progression and is a negative prognostic factor for patients’ clinical outcome.

In our own clinical cohort, co-expression of POM121 and PPARγ proteins (**S11–15**) in tumor tissue of the same CRC patient increased with age and tumor grade of dedifferentiation (Tables S5, 6), alluding at a functional role of POM121/PPARγ signaling cross-talk in vivo.

### POM121 knockout reduces proliferation of human CRC cells

We next asked if loss of POM121 affects the viability of tumor cells. HT29 cells were stably transfected with sgRNA vector targeting the 5’-prime regions of the two *POM121A/C* genes (Table S2). Clones harboring sgRNA or empty vector (EV) were assessed for mRNA and protein expression by RT-qPCR (Fig. 2B) and Western blot (Fig. 2C). Quantitative analyses and gel imaging confirmed the presence of the FL cDNA and protein in EV-transfected clonal cells. In contrary, POM121 knockout (KO) clones displayed reduced POM121 mRNA and FL protein (120–150 kDa) (*p < 0.05 *vs*. EV, 2way-ANOVA with Bonferroni post-test, *n* = 3 per clone). Rosiglitazone (rosi, 1–10 µM for 48 h), an exemplary potent and selective prescription-approved PPARγ drug agonist (IC_50_ = 100 nM), did not alter POM121 expression, rejecting the hypothesis that POM121 is a direct PPARγ target gene.

Clonal cells were then subjected to colorimetric MTT growth and viability assay (Fig. 2D). O.D. values were calculated as -fold ± S.E. (*p < 0.05 *vs*. EV, 2way-ANOVA with Bonferroni post-test, *n* = 3 per clone). POM121 KO reduced cell proliferation by ~30–40 % compared with EV control clones. To explore exemplary genes underlying this growth inhibitory effect, we treated HT29 clonal cells with PPARγ agonist as above for quantification of the cell cycle inhibitor *P21* ^*CIP1/WAF1*^. RT-qPCR analyses confirmed basal and ligand-dependent up-regulation of *P21* mRNA in POM121 KO cells compared with EV controls (*p < 0.05 *vs*. vehicle or EV, 2way-ANOVA with Bonferroni post-test, *n* = 3 per clone) (Fig. 2E).

### POM121 regulates expression of PPARγ target genes

**Fig. 3:**
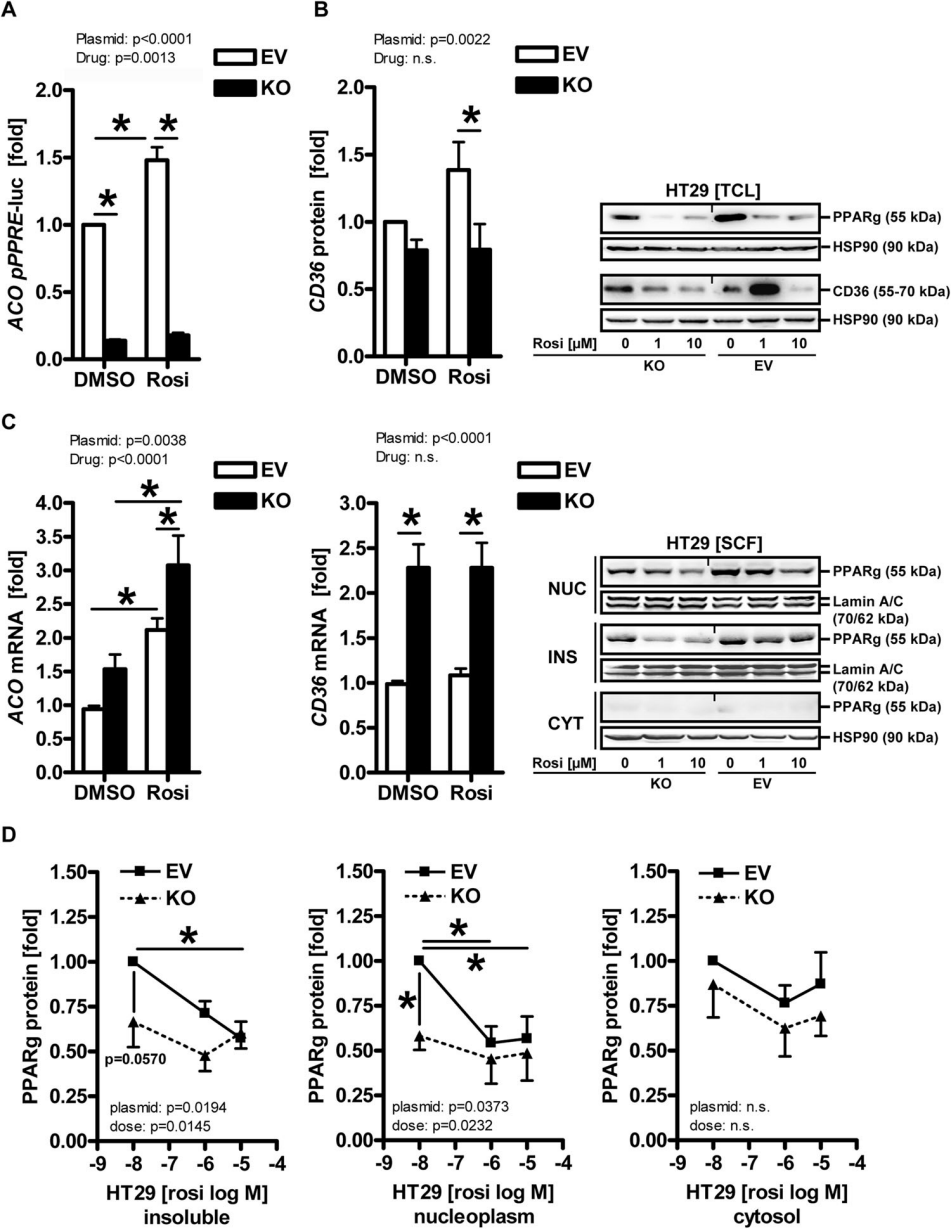
POM121 alters expression of PPARγ target genes. **A** POM121 KO reduces basal- and ligand-mediated protein expression of luciferase enzyme encoded on episomal reporter plasmids driven by DNA-binding motifs for PPARγ protein. HT29 clonal cells were transfected with a plasmid containing 3xPPREs from the enhancer region of the *ACOX1* gene followed by incubation with vehicle (DMSO) or rosi (1–10 µM) for 48 h. Luciferase activity was normalized to protein content and expressed as -fold ± S.E. (*p < 0.05 *vs*. vehicle or EV, 2way-ANOVA with Bonferroni post-tests, *n* = 3 per clone). **B** POM121 KO decreases basal- and ligand-mediated protein expression of PPARγ target genes. Clonal cells were treated as in (**A**), followed by extraction as total cell lysate (TCL). Quantitative analyses (left) and representative images (right) from Western blots. O.D. values from gels normalized to HSP90 are -fold ± S.E. (*p < 0.05 *vs*. EV, 2way-ANOVA with Bonferroni post-tests, *n* = 3 per clone). **C** POM121 KO increases basal- or ligand-mediated mRNA expression of PPARγ target genes. Clonal cells were treated as in (**A**), followed by RNA extraction. Ct-values from RT-qPCRs normalized to *B2M* are -fold ± S.E. (*p < 0.05 *vs*. vehicle or EV, 2way-ANOVA with Bonferroni post-tests, *n* = 3 per clone). **D** POM121 KO and PPARγ agonist alter the subcellular distribution of PPARγ protein. Clonal cells were treated with vehicle (DMSO) or 10 µM rosi for 48 h and subjected to subcellular fractionation (SCF). Representative images (top right) and quantitative analyses (bottom) from Western blots. O.D. values of bands in gels normalized to HSP90 or lamin A/C are -fold ± S.E. (*p < 0.05 *vs*. vehicle or EV, 2way-ANOVA with Bonferroni post-tests, *n* = 3 per clone). Legend: CYT = soluble cytoplasm; NUC = soluble nucleoplasm; INS = insoluble fraction (cytoskeleton and membrane proteins).

To test whether POM121 KO alters the transcriptional activity of PPARγ, expression of cognate target genes was measured by employing a luciferase gene reporter plasmid driven by three copies of a bona fide PPAR-responsive element (PPRE) from the enhancer of the human acyl-CoA-oxidase (*ACOX1)* gene (Fig. 3A). HT29 clonal cells were transfected with the reporter plasmid and incubated for 48 h with vehicle (DMSO) or rosi (1–10 µM) followed by measurement of luciferase counts in total cell lysates (TCL). Unexpectedly, loss of POM121 fully abrogated both basal and ligand-dependent reporter enzyme activity (*p < 0.05 *vs*. vehicle or EV, 2way-ANOVA with Bonferroni post-tests, *n* = 3 per clone), indicative of reduced binding of PPARγ protein to the PPRE in the reporter plasmid. Similar results were obtained for protein expression in TCL exemplified by the PPARγ target gene/protein CD36 (*p < 0.05 *vs*. EV, 2way-ANOVA with Bonferroni post-tests, n = 3 per clone) (Fig. 3B). These findings suggested that in absence of POM121, less PPARγ protein-dependent DNA-binding and/or protein synthesis takes place within the cytosol.

In contrary, POM121 KO increased endogenous mRNA expression of PPARγ target genes (Fig. 3C). Clonal cell lines were treated with vehicle (DMSO) or rosi (1–10 µM) for 48 h, followed by RNA extraction and RT-qPCRs detecting *ACO* and *CD36* mRNAs (*p < 0.05 *vs*. vehicle or EV, 2way-ANOVA with Bonferroni post-tests, *n* = 3 per clone). These findings suggested that in absence of POM121, more PPARγ-driven mRNA transcription occurs in the nucleus.

To evaluate if loss of POM121 also changes the localization of PPARγ, subcellular fractionation (SCF) studies were conducted with clonal HT29 cells (Fig. 3D). Overall, less PPARγ protein was found in the soluble nucleoplasm and the insoluble fraction after POM121 KO compared with EV controls (*p < 0.05 *vs*. vehicle or EV, 2way-ANOVA with Bonferroni post-tests, *n* = 3 per clone), indicative of enriched DNA-bound PPARγ in absence of POM121. Moreover, rosi led to a reduction of PPARγ protein in both TCL and SCF experiments, presumably due to receptor degradation upon ligand exposure [56]. In contrast, PPARγ levels remained low but stable in the cytosol under all conditions, and POM121 KO did not change PPARγ mRNA levels either (not shown). Conclusively, these data proposed that the integral nuclear pore protein POM121 regulates PPARγ on a post-translational level.

**Fig. 4:**
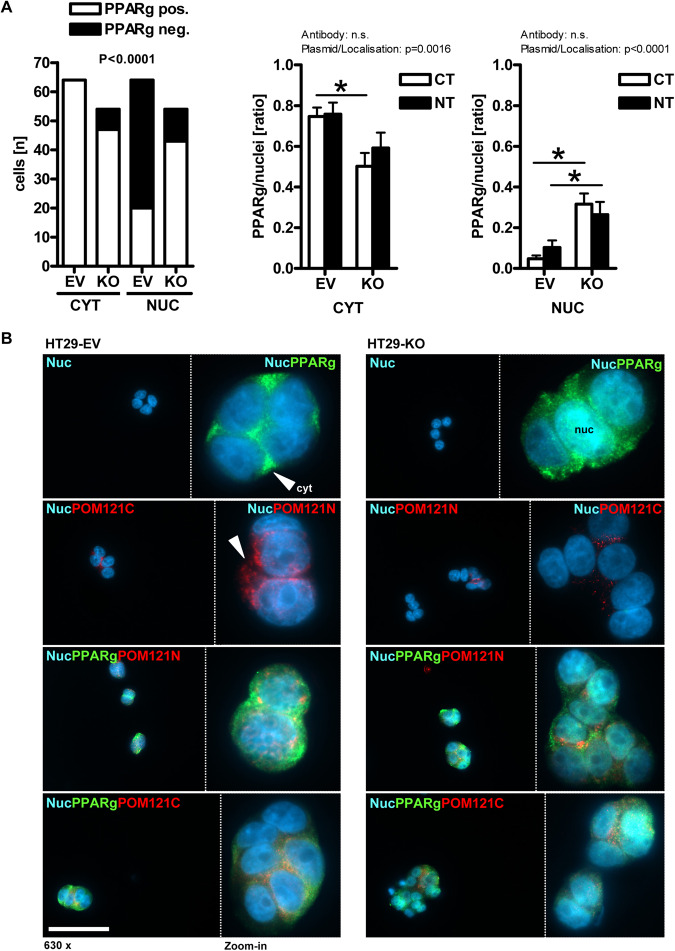
POM121 regulates nuclear transport of PPARγ. **A** POM121 KO promotes nuclear translocation of PPARγ. Clonal cells were subjected to IF microscopy using PPARγ and Abs directed against the NT or CT of POM121. Data are mean numbers of  PPARγ+ signals per cell (= DAPI+ nucleus) ± S.E. (*n* ≥ 5 cells per field, *n* ≥ 15 fields; *p < 0.05 *vs*. EV, Fisher Exact test and 2way-ANOVA with Bonferroni post-tests, *n* = 2 per clone). **B** Representative images. Color code: yellow = POM121/PPARγ (overlay); green = PPARγ; red = POM121; blue = nuclei (DAPI); scale bar = 50 µm; original magnifications 630x (with zoom-in). Arrows mark cytoplasmic (abbrev. “cyt”) PPARγ in EV *vs*. nuclear (abbrev. “nuc”) PPARγ in KO cells and perinuclear POM121+ punctae.

To underpin these data, images were collected from IF microscopy (Fig. 4). Clonal cell lines were treated as in Fig. 3 and subjected to staining with Abs against PPARγ and POM121. Fluorescence signals (*n* ≥ 5 cells per field, *n* ≥ 15 fields) showcased reduced perinuclear POM121 staining in KO clones. Notably, POM121 KO clones displayed more PPARγ positivity in the nucleus than EV cells (*p < 0.05 *vs*. EV, Fisher Exact test and 2way-ANOVA with Bonferroni post-tests, *n* = 2 per clone), which had mostly perinuclear PPARγ consistent with other transformed cell lines.

**Fig. 5:**
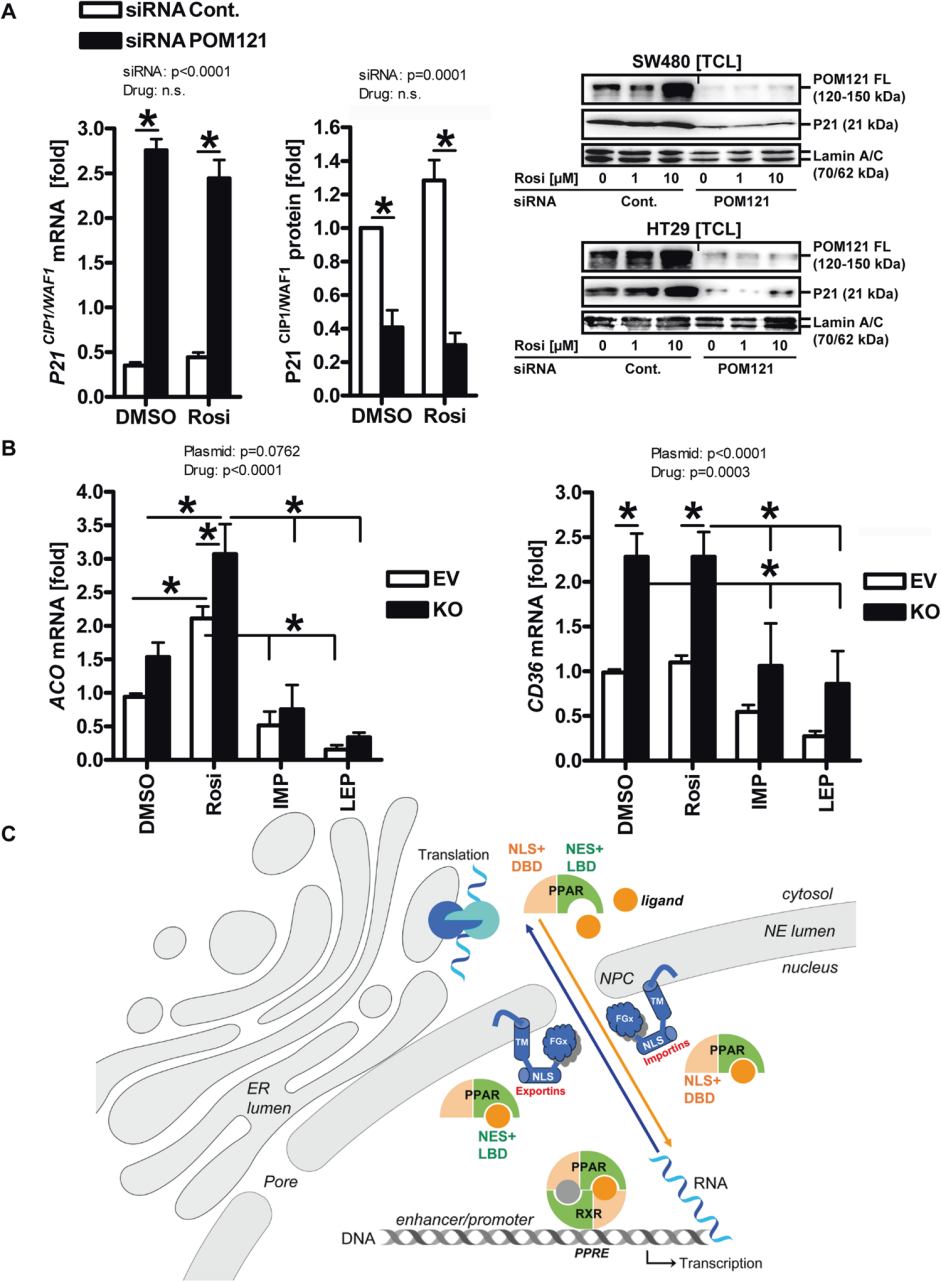
siRNA and transport inhibitors phenocopy sgRNA-mediated POM121 knockout. **A** POM121 knockdown (KD) by siRNA increases mRNA expression of PPARγ target genes but reduces protein levels. Parental HT29 cells were transiently transfected with *POM121A/C* siRNA or control siRNA for 48 h followed by extraction of RNA or total cell lysate (TCL). Left: Quantitative analyses from RT-qPCRs. Ct-values normalized to *B2M* are -fold ± S.E.; Right: Quantitative analyses and representative images from Western blots. O.D. values from gels normalized to lamin A/C are -fold ± S.E. (*p < 0.05 *vs*. control siRNA, 2way-ANOVA with Bonferroni post-tests, *n* = 3 per method). **B** Inhibitors of NPC transport mitigate the transcriptional activity of PPARγ. Clonal cells from Fig. 4 were treated with importazol, leptomycin B, vehicle (DMSO) or rosi (1–10 µM) for 48 h, followed by RNA extraction. Ct-values from RT-qPCRs normalized to *B2M* are -fold ± S.E. (*p < 0.05 *vs*. vehicle or EV, 2way-ANOVA with Bonferroni post-tests, *n* = 3 per clone). **C** Model of POM121-mediated transport of PPARγ. POM121 (FL, 120–150 kDa) as an essential integral transmembrane protein of the NPC is overexpressed in cancers and gives rise to soluble N- and C-terminal truncation variants. It has been identified to bind to PPARγ via amino acids C-terminal to its NLS and to disrupt bi-directional traffic of transcription factors (e.g., NFκB, MYC) across the nuclear pore. POM121 gain of function (GOF) reduced transcription in the nucleus and enhanced translation of PPARγ target genes/proteins in the cytosol, whereas POM121 loss of function (LOF) had the opposite effect. We propose that changes in the availability of POM121 FL protein and/or its soluble variants tip the balance between import and export of PPARγ. This “dysbalance” may be caused by competition for transport factors (importins, exportins) between POM121 and known PPARγ transport motifs: (i) the NLS in the DBD and the adjacent hinge region [73, 74] and (ii) the MEK1-binding NES-like motifs in the LBD [37, 38]. Color legend: Blue circles = ribosome; Blue cyclinders (with TM, NLS and FG domains) = POM121 (FL); Orange full circle = PPARγ-ligand (e.g., rosi); Orange quarter = PPARγ DNA-binding domain (DBD); Green quarter = PPARγ ligand-binding domain (LBD); Blue arrow = Transport nucleus-to-cytosol (mRNA translation); Orange arrow = Transport cytosol-to-nucleus (mRNA transcription).

Similar results were obtained upon knockdown (KD) of *POM121A/C* gene expression by siRNA (Fig. 5A) **(S16)**. Collectively, these findings indicate that loss of POM121 promotes the transcriptional activity of PPARγ, whereas the presence of POM121 inhibits this process.

To finally assess if the importin/exportin-RAN GTPase system contributes to the transport of PPARγ, we applied the following compounds on the HT29 clones: importazol [57], an inhibitor of cytoplasm-to-nucleus import, which interferes with the interaction between RAN GTPase and importin-β, and leptomycin B [58], which blocks exportin-1/CRM1-dependent nucleus-to-cytoplasm export (Fig. 5B). Clonal cells were treated with importazol, leptomycin B, vehicle (DMSO) or rosi (1–10 µM) for 48 h, followed by RNA extraction. As expected, RT-qPCR analyses evinced that nuclear transport inhibitors mitigated the transcriptional activity of PPARγ on the cognate target genes *ACO* and *CD36* (*p < 0.05 *vs*. vehicle or EV, 2way-ANOVA with Bonferroni post-tests, *n* = 3 per clone). Thus, both prevention of cytoplasm-to-nucleus (import) and nucleus-to-cytoplasm (export) via the NPC diminished transcription of PPARγ target genes. Again, POM121 KO clones exhibited higher mRNA expression levels of PPARγ target genes than the EV controls.

Taken together, overexpression of POM121 in CRC may inhibit the transcriptional activity of PPARγ, thereby abrogating its tumor suppressor function, e.g., via the cell cycle inhibitor P21 ^CIP1/WAF1^ (Fig. 5C).

### 3D modeling of holo-POM121 protein

Hitherto, no full structures of POM121 holo-proteins have been annotated in the PDB. To identify possible 3D analogs, I-TASSER [51] was queried using the canonical FL input peptide sequence submitted in FASTA format [UniProt ID: Q96HA1 (P121A_HUMAN), isoform 1], which is 95% identical to the one of POM121C (**S1**, Table S4), and 3D models were generated using iterative fragment assembly simulations. Outputs with predicted sequence and structural similarity are based on existing PDB entries, which were visualized by Chimera and PyMOL.

**Fig. 6:**
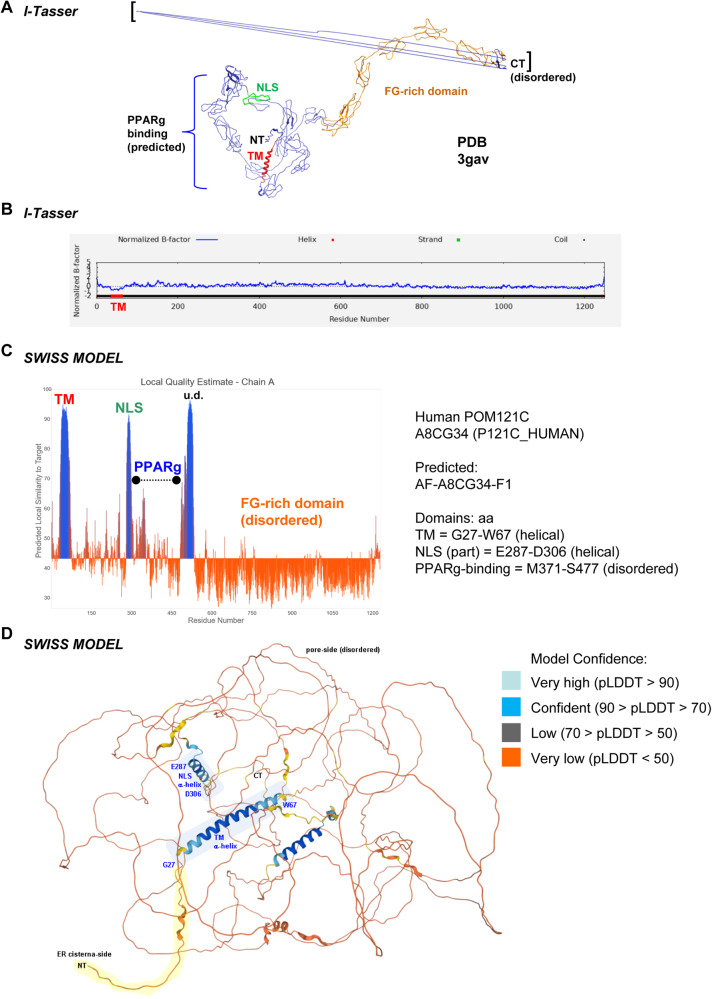
Structure prediction models of POM121 holo protein. **A** I-TASSER query of FL POM121 protein [UniProt ID: Q96HA1 (P121A_HUMAN)]. Top 5 theoretical 3D models are listed (Table S13) with C-scores [-5 (low) to +2 (high)] for model confidence: Model (Rank A) -1.19 [3gavA] is shown. Putative sequences of TM (red), NLS (green), PPARγ-binding (blue) and FG-rich (orange) domains of POM121 were mapped to model Rank A. Note the ordered N-terminal (NT) domains *vs*. the disordered/unspecified (low confidence) structure of the C-terminus (CT, black brackets). **B** B-Factor graph of FL POM121 protein [UniProt ID: Q96HA1 (P121A_HUMAN)] in the top theoretical model (Rank A) with confidence [y-axis: - (ordered) to + (disordered)]. B-Factor was lowest (most reliable) along the N-terminal TM domain, whereas peaked (most disordered) toward the C-terminus (aa 1225–1249), corresponding to the elongated irregular shape of the model in (A), consistent with the FG-repeats of the hydrophilic “basket” of the NPC protein. **C** Local Quality Estimate plot of FL POM121C protein [UniProt ID: A8CG34 (P121C_HUMAN)] for the theoretical model AF-A8CG34-F3 given by SWISS-MODEL (alpha-fold DB: average model confidence = 43.19). Legend: TM = G27 to W67 (helical); NLS (partial) = E287 to D306 (helical), PPARγ-binding peptides = M371 to S477 (disordered). **D** 3D model of FL POM121C protein [UniProt ID: A8CG34 (P121C_HUMAN)] given by SWISS-MODEL (alpha-fold DB in **C**). Model Confidence: Light blue = very high (pLDDT > 90); Dark blue = confident (90 > pLDDT > 70); Gray = low (70 > pLDDT > 50); Orange = very low (pLDDT < 50). N-terminal TM and NLS helices are highlighted as shaded blue rectangles, the N-terminus orientated towards the ER cisterna side in yellow. Note that the sequence consecutive of the NLS was predicted to be mostly disordered, including the PPARγ-peptide binding regions identified by MS (Table S3) **(S1)**. The third high-confidence helix did not overlap with the putative PPARγ-binding peptides.

I-TASSER gave five theoretical models of differing fidelity according to their confidence C-scores (Table S13) Though all models were phylogenetically and functionally unrelated to NPC proteins and had distinct structural conformations, they shared at least one α-helix at the N-terminus, as exemplified by model A (Fig. 6A).

B-Factor analysis [51] for the top predicted model A (3gavA, *Solution structure of Human Complement Factor H in 137* *mM NaCl buffer*) is shown (Fig. 6B) with B-Factor normalized score of >0 indicating instability. The B-Factor along the peptide of model A ranged from −1 to 1, with a peak occurring towards the C-terminal end. This relative instability may explain the disorder displayed at the C-terminal of model A, where the final 5 residues are unable to be modeled sufficiently and showcase a linearly elongated structure.

Likewise, SWISS-MODEL (alpha-fold DB) (Fig. 6C, D) simulations for POM121C protein [UniProt ID: A8CG34 (P121C_HUMAN)] retrieved a low confidence model of the holo protein, nonetheless again with high fidelity predictions for the N-terminal TM and NLS α-helices, whereas the C-terminal portions were of high disorder.

Notably, only the iTASSER Model C (Table S13) exhibited a structure-function relationship to POM121, as it is a transmembrane ion channel involved in cellular transport processes (5vkqA, *Structure of a mechanotransduction ion channel Drosophila NOMPC in nanodisc*). Since this protein has a similar radial symmetry like the NPC cocrystalized with “nanodiscs”, bilayered phospholipid-rich artificial membranes [59], it may be a suitable template for future simulation modeling approaches. Due to the limitation of these holo-protein models, we next resorted to modeling of subdomains.

### 3D modeling of the N-terminal TM domain

Consistent with topologies of single-pass TM proteins [60], the N-terminal domain (aa 27–67) is likely to form an α-helix preceded by an disordered N-terminus exposed into the ER lumen:.

27 GCGGPA(G/R^POM121A^)A(A/V)**LL**G**L**S**L**VG**LLL**Y**L**V***P***AAAA**L**AW**L(**A/T)VG(T/A) TAAWW 67 (**S17**). Secondary structure prediction of FL POM121C [UniProt ID: A8CG34 (P121C_HUMAN)] using *Jpred* [50] proposed an N-terminal α-helix within this peptide (aa 27–67) followed by a proline turn/kink and an elongated C-terminal tail. Several potential covalent post-translational modification (PTM) sites are present (e.g., C cysteine, Y/T/S phosphorylation). Hydrophobicity plots by EMBOSS *octanol* and *pepwindow* also suggested an amphipathic/hydrophobic N-terminal TM domain. This region is >90% (37 of 41 aa) conserved between the *POM121A/C* gene products. 3D models were then generated by *Phyre2* [52]. Best fit models (*n* = 20) were calculated based on crystallographic data from the PDB. Alignment and length coverage were highest for the transmembrane protein “*arabinofuranosyltransferase aftd2 from mycobacteria, mutant r1389s class 2*” [c6wbyA: CI 44.9 / ID 24%] corroborating the secondary structure predicted by EMBOSS, while all models proposed an N-terminal α-helix (CI 11.8–44.9 / ID 43–62%).

EZMOL visualized the N-terminal helix with exposed residues for PTMs (C/Y/T/S) and a kink formed by the central proline residue followed by an elongated tail. Simulation of the equivalent POM121A peptide with 4 aa substitutions compared with the POM121C peptide gave similar results (not shown).

### 3D modeling of the NLS/PPARγ-binding peptides

To further expand our knowledge on the POM121 interactome, the 3D structure of the region in POM121C [UniProt ID: A8CG34 (P121C_HUMAN)] identified by our MS experiments was evaluated by in silico screening in PDB and UniProt (**S18**). Query for “POM121” in the crystallographic database identified a hit comprising the mouse orthologue POM121 NLS_291–320_ bound to importin-α1 (PDB ID: 4YI0) [61]. 3D simulation of the human POM121 NLS_295–322_ peptide was also found in SWISS-MODEL (alpha-fold DB), with the sequence referring to POM121C (A8CG34). All simulations predicted an unordered, elongated conformation of the NLS. Outputs of *Phyre2* modeling gave partial helicity predictions for the human NLS of POM121

[291 SAL**K**E**K(K**^POM121C^**/**E^POM121A^**)KKR**TVEEEDQIFLDGQEN**KRRR**HDSS 323], which is >96% (26 of 27 aa) conserved between the two *POM121A*/C gene products (**S1**). The best fit model was again mouse POM121 NLS_291–320_ bound to importin-α1 (PDB ID: 4YI0) [61]. No high confidence model was retrieved for the PPARγ-binding peptides (aa 371–477) C-terminal of the NLS and identified by our MS experiments (Table S3). Simulation of equivalent POM121A peptides harboring a total of 3 aa substitutions in both regions (NLS/PPARγ) compared with POM121C gave similar results (not shown). One may thus speculate that PPARγ, bound to a disordered/elongated, surface-exposed region adjacent to the NLS, may account for the observed regulation of this transcription factor by POM121.

## Discussion

Membrane topology and biogenesis of the nuclear envelope and NPCs are driven by lipogenic enzymes (e.g., lipin) [30], consistent with the function of PPARγ in adipogenesis and fat tissue metabolism [62]. *POM121C* is a candidate gene for fasting insulin in genome-wide association studies [28] and part of a gene signature involved in glycolysis in thyroid cancer [29]. Gene-set enrichment analyses in laryngeal cancer confirmed that POM121 relates to fatty acid metabolism and PPAR signaling [22]. Consistently, our current study identified POM121 as a post-translational regulator of PPARγ. PPARγ bound to peptides of POM121C (aa 371–477) which are located C-terminally adjacent to the NLS (aa 291–323), a region of >98% identity between the *POM121A/C* transcripts and proteins. Dynein binding protein IQCG [63] and the intermediary filament component “cytoskeletal keratin type I-10” [64] were also precipitated by PPARγ, suggesting a broader cytoplasmic interactome of this transcription factor.

One limitation of our study is that we pulled down small POM121 fragments but not FL POM121 protein with PPARγ Ab. This may be explained by the existence of multiple POM121 isoforms generated by alternative splicing or translational initiation. During evolution of the NPC [65], N-terminally truncated, soluble “s”POM121 (ΔTM / NLS + ) emerged as a mobile transcriptional regulator at the chromatin and coactivator at gene promoters in the nucleoplasm [13]. N-terminally truncated, cytoplasmic POM121 (ΔTM / ΔNLS) inhibits HIV replication [14], while FL POM121 (TM + / NLS + ) promotes import of the HIV pre-integration complex [18]. The internal fragment of POM121 (ΔTM / NLS + ) is a soluble dominant-negative (DN) mutant for nuclear membrane assembly during post-mitosis and interphase [15]. POM121C peptides are elevated in the plasma of sepsis patients [17]. Consistently, the POM121 fragments identified in the current study overlap with these bioactive peptide domains.

Due to its high molecular weight, N-terminal TM and C-terminal FG-rich domains, extraction of FL POM121 protein from cells required high stringency conditions using detergents which destroyed the interaction otherwise evident upon hypotonic, detergent-free lysis (Fig. 1A). Thus, future subcloning of the N-terminal domain of POM121 until the NLS and PPARγ-binding regions shall further fine map the specific interaction sites of the two proteins.

Though there is ample knowledge on POM121 functions, no 3D model of the FL protein currently exists. Our *in-silico* predictions of the N-terminal TM domain (aa 27–67) agreed with the reports of others to form an α-helix [7], as did the NLS (aa 291–323) whose structure has been revealed in complex with importin-α1 [61]. The PPARγ-binding (aa 371–477) and FG-rich domains were simulated to adopt elongated loop-like structures, consistent with the disordered C-terminus forming the octagonal hydrophilic channel pore (basket) in the NPC holo-complex. In critical view of the limitations of *in-silico* models, the radial symmetric ion channel [59] suggested in the current study may be a suitable template for future structural simulation of FL POM121. Likewise, our *in-silico* interactome proposed that POM121, beyond the transcription factors described, binds oncogenic drivers of CRC (e.g., APC) and clinical immune checkpoints (e.g., CTLA4). In line with this repertoire of cargo/client proteins, POM121 has been shown to inhibit NFκB activity in the nuclei of inflammatory macrophages [3]. Thus, POM121 regulates transcription factors (i) by binding to and altering their subcellular localization(s) and/or (ii) by acting as a direct coregulator at the DNA.

POM121 overexpression in tumors confers unfavorable prognosis in patients [25]. Our bioinformatic analyses confirmed the enrichment of *POM121A/C* mRNA in public datasets from gastrointestinal (GI) cancers predicting poor survival and coinciding with copy-number gains or gene amplifications in both genes. Likewise, co-positivity of POM121 and PPARγ in our own clinical cohort of CRC agreed with the idea of a functional crosstalk between the two proteins. *KRAS* mutant cases, who expressed high levels of POM121 had more PPARγ in the cytoplasm, the perinuclear compartment overlapping with the ER and the nuclear envelope, than those who expressed low levels of POM121 or were *KRAS* wt, supporting our initial hypothesis that POM121 inhibits the nuclear activity of this transcription factor, especially in cases with active RAS-ERK1/2 signaling that facilitates cytosolic retention and inactivation of PPARγ [36].

On a mechanistic basis, POM121 fosters many oncogenic signaling pathways (e.g., hedgehog, P53, TGFβ/SMAD, PI3K/AKT) [21, 25, 26]. Thereby, POM121 augments cell proliferation, migration and invasion, underscoring the observed clinical associations. Conversely, loss of POM121 or perturbance of NPC function results in cell death by apoptosis or autophagy [20, 66]. As such, POM121 [67] and whole NPCs [68, 69] are proteolytically degraded by caspase-3. In mouse prostate cancer xenografts, POM121 drives tumor progression through importin-β-dependent nuclear import of androgen receptor, E2F1 and MYC, whereas importin-β inhibitor importazol attenuated tumor growth. Therein, POM121 associates with importin-α/β via the NLS, and mutation of the NLS decreases cell viability [19], suggesting that steric competition between importins and transcription factors like PPARγ at this critical POM121 protein interface may determine cell fates. Hence, POM121 acts as a driver or passenger of cell death, beyond its structural role, a feature which may be therapeutically exploitable.

Unexpectedly, silencing of gene expression from the human *POM121A/C* locus either by stable sgRNA knockout or by transient siRNA knockdown resulted in a reciprocal expression pattern of the same PPARγ target gene as exemplified here by ACO, CD36, and P21. Specifically, in absence of POM121 enhanced mRNA but reduced protein levels of the same gene were observed. However, this phenomenon may be a more general principle due the essential role of POM121 as a guardian of the NPC.

In the nucleus, loss of POM121 augmented the transcriptional activity of PPARγ at target gene promoters relevant for differentiation and cell cycle arrest (*P21 CIP1/WAF1*) and reduced cell growth in human CRC cell lines in vitro. However, this anti-proliferative effect cannot be attributed to PPARγ alone. Instead, companion transcription factors (MYC, E2F, NFκB e.a.), which are transported by POM121 as well [4], shall contribute to and converge on this cell phenotype. Thus, PPARγ adds as one novel candidate to previously known POM121 cargo/client proteins. Moreover, cofactors (e.g., SRC, NCoR e.a.) and post-translational modifications, including ubiquitination, impact the transcriptional activity of PPARγ [70]. Accordingly, POM121 deficiency precluded agonist-induced degradation of PPARγ and reduced PPARγ protein in the nucleoplasm and at structural components (e.g., cytoskeleton). In contrast, enhanced chromatin/DNA-bound PPARγ protein could be visualized in cell nuclei in situ, the proportion which could be actively responsible for the observed increase of target gene transcription upon POM121 loss.

In the cytosol, loss of POM121 resulted in reduced protein synthesis of PPARγ target genes and failure of PPARγ protein to bind and transactivate the *ACO* enhancer element in the reporter plasmid. One may speculate that POM121 transports DNA plasmids into the nucleus, supported by POM121’s involvement in entry of viral particles into the nucleus [18, 71]. In general, circular DNA plasmids remain episomal and are not integrated into the genome without forced selection by antibiotics [72], but rather transiently enter the nucleus to be transcribed. Thus, the reduction of protein levels, we see after POM121 silencing, may be attributed to a decreased global protein translation rate resulting from reduced mRNA export from the nucleus towards the cytosol in absence of POM121 (see model in Fig. 5). However, not all genes were equally affected, e.g., lamin A/C and β-actin proteins remained stable and independent of the POM121 status. Hence, POM121 seems to control the bi-directional traffic of both nucleic acids (RNA, DNA) and proteins across the NPC resulting in a “dysbalance” between transcription in the nucleus and translation in the cytosol if absent. However, this concept warrants further research.

In summary, we conclude that POM121 binds to PPARγ via a peptide region C-terminally adjacent to the NLS/importin binding site, thereby restricting bi-directional shuttling of this transcription factor between the nucleus and the cytoplasm. Since POM121 is frequently overexpressed in cancers including CRC, this post-translational inhibition of PPARγ may prevent its anti-proliferative, differentiation-promoting effects in tumor cells and also mitigate its modulatory function on metabolism and immunity. Thus, POM121 possesses potential as clinical therapy target and/or biomarker in CRC. Future studies will have to go beyond associations and explore the mechanistic connection of the aforementioned proteins with CRC, as well as relevant outcomes in patients.

### Supplementary information

Supplementary figure legends R2

Supplementary figures R2

Supplementary tables R2

Original Western Blots

## Data Availability

The data supporting the findings of this study are available within the article, its supplementary figures and tables. Original Western blots are provided as an online file. The public databases used in our study are listed in Materials and Methods. Additional information on data, materials and methods are available upon request with the corresponding author (EB).
