# Supplementary material for: Nuclear pore protein POM121 regulates subcellular localization and transcriptional activity of PPARγ
Source: Cell Death Dis. 2024 Jan 4;15(1):7. doi: 10.1038/s41419-023-06371-1 (PMC10766976; doi:10.1038/s41419-023-06371-1)
Supplement: Supplementary file 1 — Supplementary figure legends R2 [file 41419_2023_6371_MOESM1_ESM.docx]

**Legends to Supplementary Figures**

**S1 Sequence alignments of human POM121 (*POM121A)* and POM121C proteins**

NCBI BlastP alignment of the proteins. UniProt IDs: Query = Human POM121A Isoform 1 (Q96HA1-1, P121A_HUMAN); Subject = Human POM121C Isoform 1 (A8CG34, P121C_HUMAN). Color legend: green = ER cisterna-side (N-terminus); yellow = TM domain; blue = NLS; grey = PPARγ-binding region with peptides in bold (**Table S3**), red = non-matched residues; pink = start and end (C-terminus) of proline / serine / threonine-rich region; blue FGs = pore-side hydrophilic basket with FG repeats.

**S2 Sequence alignments of human POM121 (*POM121A)* and POM121C transcripts**

NCBI BlastN alignment and localisation of the sgRNA in the mRNAs. Genbank® IDs: Query = Human *POM121A* transcript variant 1; Subject = Human *POM121C* transcript variant 1. The sgRNA site was localized in proximity with and upstream of the ATG start codon in both mRNAs. Color legend: green = sgRNA targeting site; yellow = start codon CDS; blue = stop codon CDS.

**S3 Identification of protein interaction candidates for POM121**

Protein-protein interaction (PPI) network for POM121 generated from UniProt (BioGRID: n=100 interactors; evidence level=1) (**Table S7**). Concentric circles near the center represent close interactions. Blue circles are associated proteins from the same organism (e.g. *Homo sapiens*). Yellow circles showcase proteins from another organism. Yellow lines mark associations with physical evidence, purple lines those with both physical and genetic evidence. Red circles highlight cancer-relevant genes/proteins.

**S4 Correlation of *POM121* mRNA with clinical factors in CRC patients**

*POM121A/C* mRNA expression in TCGA data sets from OncoDB® (**Table S9**). Data are log2 -fold change ± S.D. (*p<0.05, t-test): READ = rectal adenocarcinoma (n=104 cases); COAD = colon adenocarcinoma (n=349 cases); STAD = stomach adenocarcinoma (n=450 cases).

**S5 Correlation of *POM121* mRNA with prognosis in CRC patients**

**A,** *POM121A/C* gene alterations. Oncoprint® files were obtained from cBioPortal® data set [Colorectal Adenocarcinoma, TCGA, PanCancer Atlas (n=594 cases)], Left: Percent (%) altered cases compared with the total patient number (**Table S10)**. Right: Vertical bars representing samples from patients without gene alterations were marked in grey or cut off.

**B**, Kaplan-Meier survival curve analysis query of cBioPortal® data sets (**Table S11)** from PanCancer cohorts (n=10 studies; n=76639 cases, *POM121C*: p=0.110; *POM121A* p=0.302) including all genomic alterations (mRNA changes, mutations, amplifications, deletions). Log-rank tests for overall survival (OS) are depicted in the graph Legend: Blue “WT” = wildtype / unaltered genes; Red “ALT” = altered genes.

**S6 Correlation of *POM121A* mRNA with prognosis in CRC patients**

Kaplan-Meier survival curve analysis was performed based on the cBioPortal® data set provided in **S5a** [Colorectal Adenocarcinoma, TCGA, PanCancer Atlas (n=594 cases)]. Advanced analysis query using *POM121A* mRNA expression cutoffs of >1.28 and <-1.28: a) Chart depicting proportion of samples that fit definition of high *vs.* low mRNA, with 28% of samples fitting this range (n=149); of that 28% of samples, 79% (n=118) were classified as mRNA high and 21% (n=31) as mRNA low. b) In the curve of the 28% of samples, the high expression group had a lower OS (*p=0.0069). c) DFS (p=0.414). d) PFS was lower among the mRNA high group (*p=0.0342). d) DSS was lower among the mRNA high group (*p=0.0202). Legend: OS = overall; PFS = progression-free; DFS = disease-free; DSS = disease-specific survival.

**S7 Correlation of *POM121C* mRNA with prognosis in CRC patients**

Advanced analysis query of cBioPortal® data for *POM121C* mRNA expression as in **S6**. a) 26% of samples fit this range (n=138); of that 26% of samples, 78% (n=107) were classified as mRNA high and 22% (n=31) as mRNA low. b) OS for mRNA high trends lower (p=0.0553). c) DFS (p=0.147). d) PFS was lower among the mRNA high group (*p=0.0062). e) DSS was lower among the mRNA high group (*p=0.0306).

**S8 Correlation of *POM121* mRNA with subtypes in CRC patients**

**A-B**, Association of *POM121A/C* mRNAs with clinical factors. Data were retrieved from cBioportal® data sets [Colorectal Adenocarcinoma, TCGA, PanCancer Atlas (n=594 cases) and Nature 2012 (n=276 cases] and calculated as -fold change ± S.E. compared to the mean of all diploid samples (*p<0.05, Fisher Exact or Kruskal-Wallis test with Tukey post-test). Legend: CNA = copy number alteration; MSI/CIMP = microsatellite instable/CpG island methylator phenotype; INV/GS = invasive/genomically stable; CIN = chromosomal instable; AMP = amplification; DEL = deletion; DIPL = diploid. Note enrichment of *POM121A/C* mRNA in CIN+ samples.

**S9 Mutation profiling of *POM121A* mRNAs in human cancers**

*POM121A* gene alterations (**A**) and mutations (**B,C**) were retrieved from GISTIC entries in cBioportal® data sets from cell lines [CCLE and NCI60: n=2826 lines] and patients [“Bowel” including CRC: n=4535 cases; n=13 studies] (**Table S12)**. Legend: TM = trans-membrane; s = soluble, dn = dominant-negative; NLS = nuclear location sequence; CT = C-terminus (protein).

**S10 Mutation profiling of *POM121C* mRNAs in human cancers**

*POM121C* gene alterations (**A**) and mutations (**B,C**) were retrieved from GISTIC entries in cBioportal® data sets from cell lines [CCLE and NCI60: n=2826 lines] and patients [“Bowel” including CRC: n=4535 cases; n=13 studies] (**Table S12)** as in **S9**.

**S11 Supportive images on POM121/PPARγ protein expression in CRC tissues**

FFPE sections from patients’ TMAs were stained with Abs against PPARγ (left) and POM121 (right) by immunohistochemistry (IHC) (**Tables S5-6**). Representative pictures of negative (*score 0) vs*. positive (*scores* *2/3*) stainings in tumor tissues; scale bar = 50 µm; original magnifications 200-400x. Legend: NC = normal colon; TU = tumor.

**S12** **Correlation of POM121/PPARγ protein expression with clinical factors**

Staining scores in tumor and stroma cells from TMA images (as exemplified in **S11)** were quantified upon dichotome grouping into double negative (Abbrev. “LL”, *scores 0/1*) *vs.* double positive (Abbrev. “HH”, *scores 2/3*) (n=208, *p<0.05, Mann Whitney and Fisher Exact tests). Single positive cases were excluded. Detailed statistics is presented in **Tables S5-6**.

**S13** **Correlation of POM121/PPARγ protein expression with patients’ survival status**

Staining scores were quantified as described in **S12**.

Detailed statistics is presented in **Tables S5-6**.

**S14** **Correlation of POM121/PPARγ protein expression with patients’ overall survival**

Kaplan-Meier survival curves were calculated from positivity scores in tumor and stroma cells. Scores were quantified from TMA images (as exemplified in **S11)** upon dichotome grouping into double negative (Abbrev. “LL”, *scores 0/1*) *vs.* double positive (Abbrev. “HH”, *scores 2/3*) (n=208, *p<0.05, log-rank tests). Single positive cases are included (Abbrev. “L” = low *vs.* “H” = high) for each protein. Detailed statistics is presented in **Tables S5-6** and log-rank tests depicted in the graphs. Legend: OS = Overall Survival.

**S15** **Correlation of POM121/PPARγ protein expression with patients’ disease-free survival**

Kaplan-Meier survival curves were calculated as in **S14**. Detailed statistics is presented in **Tables S5-6** and log-rank tests depicted in the graphs. Legend: DFS = Disease-Free Survival.

**S16 Supportive data on POM121 knockdown by siRNA**

**A,** POM121 siRNA decreases POM121 mRNA and protein. Parental *BRAFV600E* HT29 cells were transiently transfected with *POM121A/C* siRNA or control siRNA for 48 h followed by extraction of RNA or total cell lysate (TCL), respectively. Left: Quantitative analyses from RT-qPCRs. Ct-values normalized to *B2M* are -fold ± S.E.; Right: Quantitative analyses from Western blots. O.D. values from gels normalized to HSP90 are -fold ± S.E. (*p<0.05 *vs*. control siRNA, 2way-ANOVA with Bonferroni post-tests, n=3 per method).

**B,** POM121 siRNA reduces cell proliferation. HT29 cells were transfected as in A and viability measured by colorimetric MTT assay. O.D. values were calculated as -fold ± S.E. compared with day 0 (*p<0.05 *vs.* control siRNA or day, 2way-ANOVA with Bonferroni post-tests, n=3).

**C**, POM121 siRNA increases mRNA but reduces protein expression of PPARγ target genes. Parental *KRASG12V* SW480 cells were transfected and data analysed as in A (*p<0.05 *vs*. vehicle or control siRNA, 2way-ANOVA with Bonferroni post-tests, n=3 per method). Insert: Representative images from Western blots.

**D,** POM121 siRNA lowers basal- and ligand-mediated protein expression of luciferase enzyme encoded on episomal reporter plasmids driven by DNA-binding motifs for PPARγ protein. *KRAS* wt HEK293T non-cancer control and two parental *KRAS* mutant human CRC cell lines (*KRASG13D* HCT116, *KRASG12V* SW480) were transiently transfected with *POM121A/C* siRNA or control siRNA together with a reporter gene plasmid containing 3xPPREs from the enhancer region of the *ACOX1* gene followed by incubation with vehicle (DMSO) or rosi (1-10 µM) for 48 h. Luciferase activity was normalized to protein content and expressed as -fold ± S.E. (*p<0.05 *vs.* vehicle or control siRNA, 2way-ANOVA with Bonferroni post-tests, n=3 per cell line).

**S17 Supportive 3D prediction models of the N-terminal TM domain**

**A,** Primary sequence of the N-terminal TM domain (aa 27-67) of POM121C [UniProt ID: A8CG34 (P121C_HUMAN)]: 27 GCGGPAGAALLGLSLVGLLLYLVPAAAALAWLA VGTTAAWW 67. Secondary structure prediction using *Jpred4* proposed an N-terminal α-helix within this peptide. This region is >90% (37 of 41 aa) conserved between the *POM121A/C* gene products (**S1**). Color legend: green = β-sheet; red = α-helix.

**B,** Hydrophobicity plots by EMBOSS *octanol* and *pepwindow* also suggest formation of an amphipathic/hydrophobic N-terminal TM domain. Color legend: red = region of maximal hydrophobicity (aa 1-100).

**C,** 3D models by *Phyre2*. Top: Peptide sequence from N to C terminal with secondary structure predictions. Bottom: Best fit models (n=20) generated based on PDB data. Alignment and length coverage were highest for transmembrane protein: “*arabinofuranosyltransferase aftd2 from mycobacteria, mutant r1389s class 2*” [c6wbyA: CI 44.9 / ID 24 %]. All models proposed the N-terminal α-helix (CI 11.8-44.9 / ID 43-62 %).

**D**, EZMOL visualized the N-terminal helix with exposed residues for post-translational modifications (PTMs) (e.g. oxidation at C or phosphorylation at Y/T/S residues) and a turn/kink formed by the central proline residue followed by an elongated C-terminal tail.

**S18 Supportive 3D prediction models of the NLS/PPARγ-binding peptides**

**A,** X-ray diffraction structure of rat POM121 NLS 291-320 peptide [orange; UniProt ID: P52591 (PO121_RAT); 291 LKEKKKRTVAEEDQLHLDGQENKRRRHDSS 320] bound to FL mouse importin-α1 protein (green) [PDB ID: 4YI0].

**B,** 3D model of human POM121C NLS 295-322 peptide by SWISS-MODEL (alpha-fold DB) [UniProt ID: A8CG34 (P121C_HUMAN); 295 EKKKKRTVEEEDQIFLDGQENKRRRHDS 322]. Top: Peptide sequence from N to C terminal; Bottom: Representation of the relative position of this NLS compared to the holo-protein. Images adapted from (https://swissmodel. expasy.org/repository/uniprot).

**C**, 3D model of human POM121 NLS 291-323 by *Phyre2* [UniProt ID: A8CG34 (P121C_HUMAN); 291 SALKEKKKKRTVEEEDQIFLDGQENKRRRHDSS 323]. Top: Peptide sequence from N to C terminal with secondary structure predictions. Bottom: Best fit models (n=6) generated based on PDB [Top Rank: rat POM121 NLS 291-320 (PDB ID: 4YI0)]. Alignment and length coverage were highest for “*structure of mouse importin a1 bound to pom121nls*” [c4yi0A: CI 99.6 / ID 82 %]. Bottom: *Phyre2* visualizing elongated features of the NLS peptide. Outputs of modeling gave partial helicity predictions for the NLS (aa 291-323), which is >97% (32 of 33 aa) conserved between *POM121A/C* gene products (**S1**).

**D**, *Phyre2* visualizing elongated features of the predicted PPARγ-binding region of human POM121 [UniProt ID: A8CG34 (P121C_HUMAN); 371 MSSL>…>TPGS 477]. Outputs of modeling gave partial helicity predictions for the PPARγ-binding peptides identified by MS (**Table S3**), located C-terminal to the NLS and which are >98% (105 of 107 aa) conserved between *POM121A/C* gene products (**S1**). No model of confidence was predicted.
