# Supplementary material for: Nuclear pore protein POM121 regulates subcellular localization and transcriptional activity of PPARγ
Source: Cell Death Dis. 2024 Jan 4;15(1):7. doi: 10.1038/s41419-023-06371-1 (PMC10766976; doi:10.1038/s41419-023-06371-1)
Supplement: Supplementary file 2 — Supplementary figures R2 [file 41419_2023_6371_MOESM2_ESM.pdf]

S1

Alignment of human *POM121A* and *POM121C* full-length proteins *

* UniProt identifiers:
Query = Human POM121A Isoform 1 (Q96HA1-1, P121A_HUMAN)
Subject = Human POM121C Isoform 1 (A8CG34, P121C_HUMAN)

Statistics for match: from NCBI BLASTP

Identities	Positives	Gaps
1183/1251(95%)	1192/1251(95%)	26/1251(2%)

Color legend: green = ER cisterna-side; yellow = TM domain; **blue** = NLS; grey = PPARg-binding region with peptides in bold (**Table S3**); red = non-matched residues; pink = proline / serine / threonine-rich region (start&end); blue = pore-side hydrophilic basket with FG repeats

```
Query  1     MSPAAAAAGAGERRRPIASVRDGRGRGCGGPARAVLLGLSLVGLLLYLVPAAAALAWLTV  60
             MSPAAAAAGAGERRRPIASVRDGRGRGCGGPA A LLGLSLVGLLLYLVPAAAALAWL V
Sbjct  1     MSPAAAAAGAGERRRPIASVRDGRGRGCGGPAGAALLGLSLVGLLLYLVPAAAALAWLAV  60

Query  61    GATAAWWGLSREPRGSRPLSSFVRKARHRRPLSSFVRKARHRRTLFASPLAKSTANGNLL  120
             G TAAWWGLSREPRGSRPLSSFV+             KARHRRTLFASP AKSTANGNLL
Sbjct  61    GTTAAWWGLSREPRGSRPLSSFVQ-------------KARHRRTLFASPPAKSTANGNLL  107

Query  121   EPRTLLEGPDPAELLLMGSYLGKPGPPQPAAAPEGQDLRDRPGRRPPARPAPRSPPPRSP  180
             EPRTLLEGPDPAELLLMGSYLGKPGPPQPA APEGQDLR+RPGRRPPARPAPRS PP
Sbjct  108   EPRTLLEGPDPAELLLMGSYLGKPGPPQPAPAPEGQDLRNRPGRRPPARPAPRSTPP---  164

Query  181   PPRSPPPSPPTHRAHHVYPSLPTPLLRPSRRPSPRDCGTLPNRFVITPRRRYPIHQAQYS  240
                    S PTHR HH YPSLPTPLLRPS RPSPRD GTLP+RFVITPRRRYPIHQ QYS
Sbjct  165   -------SQPTHRVHHFYPSLPTPLLRPSGRPSPRDRGTLPDRFVITPRRRYPIHQTQYS  217

Query  241   CLGVLPTVCWNGYHKKAVLSPRNSRMVCSPVTVRIAPPDRRFSRSAIPEQIISSTLSSPS  300
             C GVLPTVCWNGYHKKAVLSPRNSRMVCSPVTVRIAPPDRRFSRSAIPEQIISSTLSSPS
Sbjct  218   CPGVLPTVCWNGYHKKAVLSPRNSRMVCSPVTVRIAPPDRRFSRSAIPEQIISSTLSSPS  277

Query  301   SNAPDPCAKETVLSALKEKEKKRTVEEEDQIFLDGQENKRRRHDSSGSGHSAFEPLVANG  360
             SNAPDPCAKETVLSALKEK+KKRTVEEEDQIFLDGQENKRRRHDSSGSGHSAFEPLVA+G
Sbjct  278   SNAPDPCAKETVLSALKEKKKKRTVEEEDQIFLDGQENKRRRHDSS GSGHSAFEPLVASG  337

Query  361   VPASFVPKPGSLKRGLNSQSSDDHLNKRSRSSSMSSLTGAYASGIPSSSRNAITSSYSST  420
             VPASFVPKPGSLKRGLNSQSSDDHLNKRSRSSSMSSLTGAY SGIPSSSRNAITSSYSST
Sbjct  338   VPASFVPKPGSLKRGLNSQSSDDHLNKRSRSSSMSSLTGAYTSGIPSSSRNAITSSYSST  397

Query  421   RGISQLWKRNGPSSSPFSSPASSRSQTPERPAKKIREEELCHHSSSSTPLAADRESQGEK  480
             RGISQLWKRNGPSSSPFSSPASSRSQTPERPAKKIREEELCHHSSSSTPLAAD+ESQGEK
Sbjct  398   RGISQLWKRNGPSSSPFSSPASSRSQTPERPAKKIREEELCHHSSSSTPLAADKESQGEK  457

Query  481   AADTTPRKKQNSNSQSTPGSSGQRKRKVQLLPSRRGEQLTLPPPPQLGYSITAEDLDLEK  540
             AADTTPRKKQNSNSQSTPGSSGQRKRKVQLLPSRRGEQLTLPPPPQLGYSITAEDLDLEK
Sbjct  458   AADTTPRKKQNSNSQSTPGSSGQRKRKVQLLPSRRGEQLTLPPPPQLGYSITAEDLDLEK  517

Query  541   KASLQWFNQALEDKSDAASNSVTETPPITQPSFTFTLPAAAPASPPTSLLAPSTNPLLES  600
             KASLQWFNQALEDKSDAASNSVTETPP TQPSFTFTLPAAA ASPPTSLLAPSTNPLLES
Sbjct  518   KASLQWFNQALEDKSDAASNSVTETPPTTQPSFTFTLPAAATASPPTSLLAPSTNPLLES  577

Query  601   LKKMQTPPSLPPCPESAGAATTEALSPPKTPSLLPPLGLSQSGPPGLLPSPSFDSKPPTT  660
             LKKMQTPPSLPPCPESAGAATTEALSPPKTPSLLPPLGLSQSGPPGLLPSPSFDSKPPTT
Sbjct  578   LKKMQTPPSLPPCPESAGAATTEALSPPKTPSLLPPLGLSQSGPPGLLPSPSFDSKPPTT  637

Query  661   LLGLIPAPSMVPATDTKAPPTLQAETATKPQATSAPSPAPKQSFLFGTQNTSPSSPAAPA  720
             LLGLIPAPSMVPATDTKAPPTLQAETATKPQATSAPSPAPKQSFLFGTQNTSPSSPAAPA
Sbjct  638   LLGLIPAPSMVPATDTKAPPTLQAETATKPQATSAPSPAPKQSFLFGTQNTSPSSPAAPA  697

Query  721   ASSAPPMFKPIFTAPPKSEKEGPTPPGPSVTATAPSSSSLPTTTSTTAPTFQPVFSSMGP  780
             ASSA PMFKPIFTAPPKSEKEG TPPGPSV+ATAPSSSSLPTTTSTTAPTFQPVFSSMGP
Sbjct  698   ASSASPMFKPIFTAPPKSEKEGLTPPGPSVSATAPSSSSLPTTTSTTAPTFQPVFSSMGP  757

Query  781   PASVPLPAPFFKQTTTPATAPTTTAPLFTGLASATSAVAPITSASPSTDSASKPAFGFGI  840
             PASVPLPAPFFKQTTTPATAPTTTAPLFTGLASATSAVAPITSASPSTDSASKPAFGFGI
Sbjct  758   PASVPLPAPFFKQTTTPATAPTTTAPLFTGLASATSAVAPITSASPSTDSASKPAFGFGI  817

Query  841   NSVSSSSVSTTTSTATAASQPFLFGAPQASAASFTPAMGSIFQFGKPPALPTTTTVTTFS  900
             NSVSSSSVSTTTSTATAASQPFLFGAPQASAASFTPAMGSIFQFGKPPALPTTTTVTTFS
Sbjct  818   NSVSSSSVSTTTSTATAASQPFLFGAPQASAASFTPAMGSIFQFGKPPALPTTTTVTTFS  877

Query  901   QSLHTAVPTATSSSAADFSGFGSTLATSAPATSSQPTLTFSNTSTPTFNIPFGSSAKSPL  960
             QSL TAVPTATSSSAADFSGFGSTLATSAPATSSQPTLTFSNTSTPTFNIPFGSSAKSPL
Sbjct  878   QSLPTAVPTATSSSAADFSGFGSTLATSAPATSSQPTLTFSNTSTPTFNIPFGSSAKSPL  937

Query  961   PSYPGANPQPAFGAAEGQPPGAAKPALAPSFGSSFTFGNS--AAPAAAPTPAPPSMIKVV  1018
             PSYPGANPQPAFGAAEGQPPGAAKPAL PSFGSSFTFGNS   APA APTPAP S IK+V
Sbjct  938   PSYPGANPQPAFGAAEGQPPGAAKPALTPSFGSSFTFGNSAAPAPATAPTPAPASTIKIV  997

Query  1019  PAYVPTPIHPIFGGATHSAFGLKATASAFGAPASSQPAFGGSTAVF-FGAATSSGFGATT  1077
             PA+VPTPI P FGGATHSAFGLKATASAFGAPASSQPAFGGSTAVF FGAATSSGFGATT
Sbjct  998   PAHVPTPIQPTFGGATHSAFGLKATASAFGAPASSQPAFGGSTAVFSFGAATSSGFGATT  1057

Query  1078  QTASSGSSSSVFGSTTPSPFTFGGSAAPAGSGSFGINVATPGSSTTTGAFSFGAGQSGST  1137
             QTASSGSSSSVFGSTTPSPFTFGGSAAPAGSGSFGINVATPGSS TTGAFSFGAGQSGST
Sbjct  1058  QTASSGSSSSVFGSTTPSPFTFGGSAAPAGSGSFGINVATPGSSATTGAFSFGAGQSGST  1117

Query  1138  ATSTPFAGGLGQNALGTTGQSTPFAFNVSSTTESKPVFGGTATPTFGLNTPAPGVGTSGS  1197
             ATSTPF GGLGQNALGTTGQSTPFAFNV STTESKPVFGGTATPTFG NTPAPGVGTSGS
Sbjct  1118  ATSTPFTGGLGQNALGTTGQSTPFAFNVGSTTESKPVFGGTATPTFGQNTPAPGVGTSGS  1177

Query  1198  SLSFGASSAPAQGFVGVAPFGSAALSFSIGAGSKTPGARQRLQARRQHTRK  1248
             SLSFGASSAPAQGFVGV PFGSAA SFSIGAGSKTPGARQRLQARRQHTRK
Sbjct  1178  SLSFGASSAPAQGFVGVGPFGSAAPSFSIGAGSKTPGARQRLQARRQHTRK  1228
```

S2

Alignment of human *POM121A* and *POM121C* full-length mRNAs *

* GenBank identifiers:
Query = Human *POM121A* Transcript Variant 1 (NM_001257190.3)
Subject = Human *POM121C* Transcript Variant 1 (NM_001099415.3)

Statistics for match: from NCBI BLASTN

Identities	Gaps	Strand
2966/3019(98%)	9/3019(0%)	Plus/Plus

Color legend: green = sgRNA targeting site; yellow = start codon CDS; blue = stop codon CDS

```
Query  898   GGGATTGTGGGACTTTACCAAATCGGTTTGTAATAACACCTAGAAGACGCTATCCGATCC  957
             ||||| |||||||||||||| |||||||||||||||||||| ||||||||||||||||||
Sbjct  742   GGGATCGTGGGACTTTACCAGATCGGTTTGTAATAACACCTCGAAGACGCTATCCGATCC  801

Query  958   ATCAGGCCCAGTATTCCTGTCTGGGGGTACTTCCCACCGTGTGCTGGAATGGTTATCACA  1017
             ||||| ||||||||||||||| ||||||||||||||| ||||||||||||||||||||||
Sbjct  802   ATCAGACCCAGTATTCCTGTCCGGGGGTACTTCCCACAGTGTGCTGGAATGGTTATCACA  861

Query  1018  AGAAGGCTGTGCTGTCCCCTCGCAACTCCAGGATGGTGTGTAGCCCAGTGACTGTGAGGA  1077
             ||||||||||||||||||||||||||||||||||||||||||||||||||||||||||||
Sbjct  862   AGAAGGCTGTGCTGTCCCCTCGCAACTCCAGGATGGTGTGTAGCCCAGTGACTGTGAGGA  921

Query  1078  TCGCCCCTCCTGACAGAAGATTTTCGCGTTCTGCGATACCAGAGCAGATAATCAGCTCAA  1137
             ||||||||||||||||||||||||| ||||||||||||||||||||||||||||||||||
Sbjct  922   TCGCCCCTCCTGACAGAAGATTTTCACGTTCTGCGATACCAGAGCAGATAATCAGCTCAA  981

Query  1138  CACTGTCCTCACCATCAAGTAACGCCCCAGACCCATGTGCAAAGGAGACAGTACTGAGTG  1197
             ||||||| |||||||||||||| |||||||||||||||||||||||||| ||||||||||
Sbjct  982   CACTGTCGTCACCATCAAGTAATGCCCCAGACCCATGTGCAAAGGAGACTGTACTGAGTG  1041

Query  1198  CCCTCAAAGAGAAGGAGAAGAAAAGGACAGTGGAGGAAGAAGACCAAATATTCCTTGATG  1257
             |||||||||||||| |||||||||||||||||||||||||||||||||||||||||||||
Sbjct  1042  CCCTCAAAGAGAAGAAGAAGAAAAGGACAGTGGAGGAAGAAGACCAAATATTCCTTGATG  1101

Query  1258  GCCAGGAAAATAAAAGAAGGCGCCATGATAGCAGTGGCAGTGGACATTCAGCATTTGAGC  1317
             ||||||||||||||||||||||||||||||||||||||||||||||||||||||||||||
Sbjct  1102  GCCAGGAAAATAAAAGAAGGCGCCATGATAGCAGTGGCAGTGGACATTCAGCATTTGAGC  1161

Query  1318  CCCTGGTGGCCAATGGAGTCCCCGCTTCTTTTGTGCCTAAGCCTGGGTCTCTGAAGAGAG  1377
             |||||||||||| |||||||||||||||||||||||||||||||||||||||||||||||
Sbjct  1162  CCCTGGTGGCCAGTGGAGTCCCCGCTTCTTTTGTGCCTAAGCCTGGGTCTCTGAAGAGAG  1221

Query  1378  GCCTCAATTCTCAGAGCTCAGATGACCACTTGAATAAGAGATCCCGAAGCTCTTCCATGA  1437
             ||||||||||||||||||||||||||||||||||||||||||||||||||||||||||||
Sbjct  1222  GCCTCAATTCTCAGAGCTCAGATGACCACTTGAATAAGAGATCCCGAAGCTCTTCCATGA  1281

Query  1438  GCTCCTTGACAGGCGCTTACGCAAGTGGCATCCCTAGCTCCAGCCGCAATGCCATTACCA  1497
             |||||||||||||||||||| |||||||||||||||||||||||||||||||||||||||
Sbjct  1282  GCTCCTTGACAGGCGCTTACACAAGTGGCATCCCTAGCTCCAGCCGCAATGCCATTACCA  1341

Query  1498  GTTCCTACAGCTCCACTCGAGGCATCTCACAGCTCTGGAAGAGAAATGGCCCCAGTTCAT  1557
             |||||||||||||||||||||||||||||||||| |||||||||||||||||||||||||
Sbjct  1342  GTTCCTACAGCTCCACTCGAGGCATCTCACAGCTGTGGAAGAGAAATGGCCCCAGTTCAT  1401

Query  1558  CACCCTTCTCTAGCCCAGCCTCCTCCCGCTCCCAGACACCGGAGAGGCCAGCAAAGAAAA  1617
             |||||||||||||||||||||| |||||||||||||||||||||||||||||||||||||
Sbjct  1402  CACCCTTCTCTAGCCCAGCCTCATCCCGCTCCCAGACACCGGAGAGGCCAGCAAAGAAAA  1461

Query  1618  TAAGAGAAGAGGAGCTGTGTCATCATTCCAGTTCTTCAACTCCATTGGCAGCAGACAGGG  1677
             |||||||||| |||||||||||||||||||||||||||||||||||||||||||||| ||
Sbjct  1462  TAAGAGAAGAAGAGCTGTGTCATCATTCCAGTTCTTCAACTCCATTGGCAGCAGACAAGG  1521

Query  1678  AGTCCCAGGGAGAAAAGGCTGCAGATACAACCCCAAGGAAGAAACAAAACTCGAATTCTC  1737
             ||||||||||||||||||||||||||||||||||||||||||||||||||||||||||||
Sbjct  1522  AGTCCCAGGGAGAAAAGGCTGCAGATACAACCCCAAGGAAGAAACAAAACTCGAATTCTC  1581

Query  1738  AGTCTACACCTGGCAGCTCTGGGCAGCGTAAGCGGAAAGTTCAGCTGCTGCCTTCTCGGC  1797
             ||||||||||||||||||||||||||||||||||||||||||||||||||||||||||||
Sbjct  1582  AGTCTACACCTGGCAGCTCTGGGCAGCGTAAGCGGAAAGTTCAGCTGCTGCCTTCTCGGC  1641
{continued until}
Query  3889  TTGGTGTTGCACCTTTCGG  3907
             ||||||||| ||| |||||
Sbjct  3742  TTGGTGTTGGACCGTTCGG  3760   >>> TGA (stop codon at >4000)
```

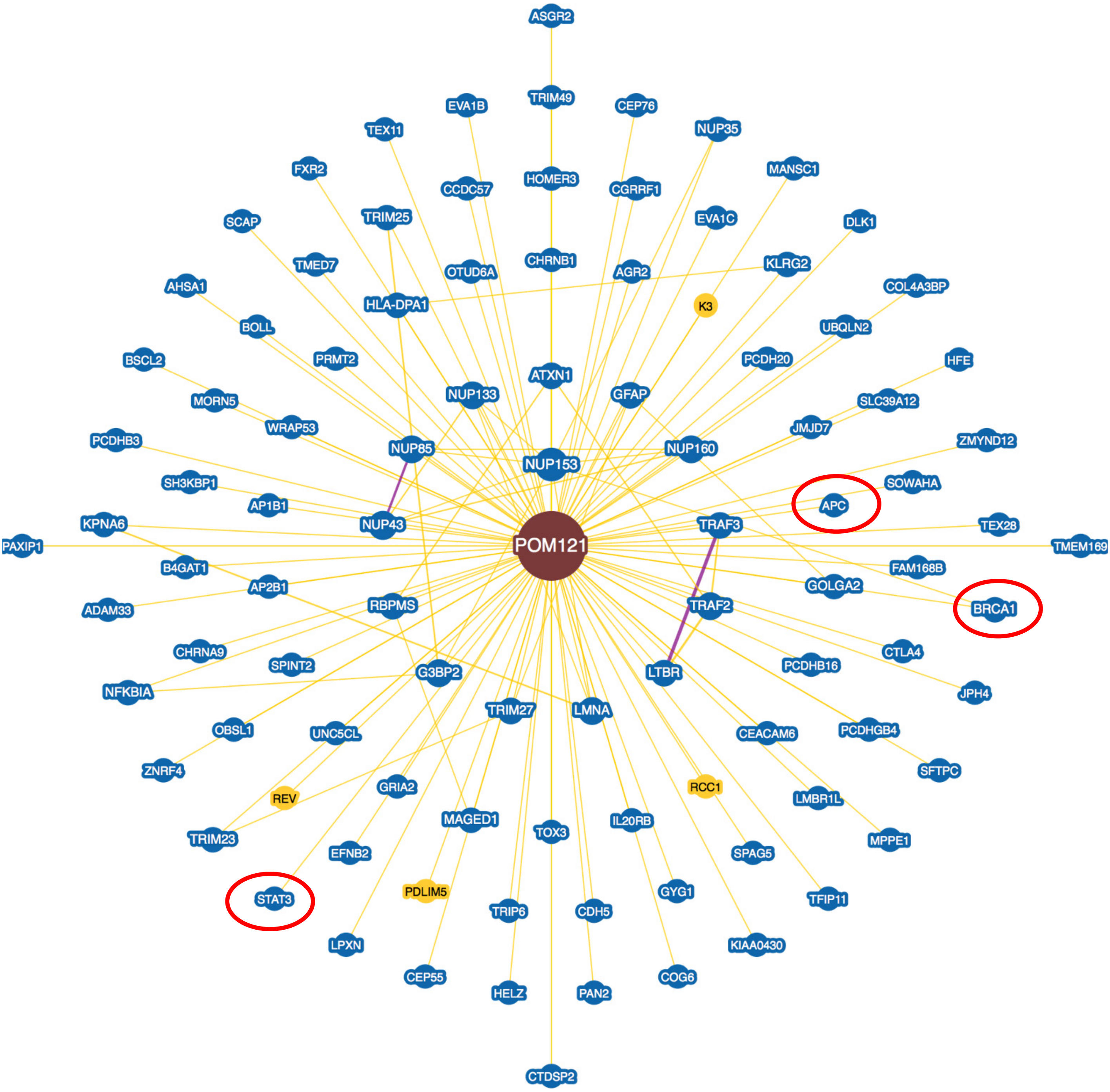
ASGR2
TRIM49
EVA1B
CEP76
TEX11
NUP35
FXR2
HOMER3
CCDC57
CGRRF1
MANSC1
SCAP
TRIM25
EVA1C
DLK1
TMED7
CHRNB1
OTUD6A
AGR2
KLRG2
AHSA1
COL4A3BP
HLA-DPA1
K3
BOLL
UBQLN2
BSCL2
PRMT2
PCDH20
HFE
ATXN1
MORN5
NUP133
GFAP
SLC39A12
WRAP53
JMJD7
PCDHB3
ZMYND12
NUP85
NUP160
NUP153
SH3KBP1
SOWAHA
AP1B1
APC
KPNA6
NUP43
TRAF3
TEX28
PAXIP1
POM121
TMEM169
B4GAT1
FAM168B
AP2B1
GOLGA2
ADAM33
RBPMS
TRAF2
BRCA1
CHRNA9
CTLA4
SPINT2
G3BP2
LTBR
PCDHB16
NFKBIA
JPH4
TRIM27
LMNA
OBSL1
UNC5CL
CEACAM6
PCDHGB4
ZNRF4
SFTPC
RCC1
REV
GRIA2
LMBR1L
MAGED1
IL20RB
TOX3
TRIM23
MPPE1
EFNB2
SPAG5
GYG1
PDLIM5
STAT3
TFIP11
TRIP6
CDH5
LPXN
KIAA0430
CEP55
COG6
HELZ
PAN2
CTDSP2

S3

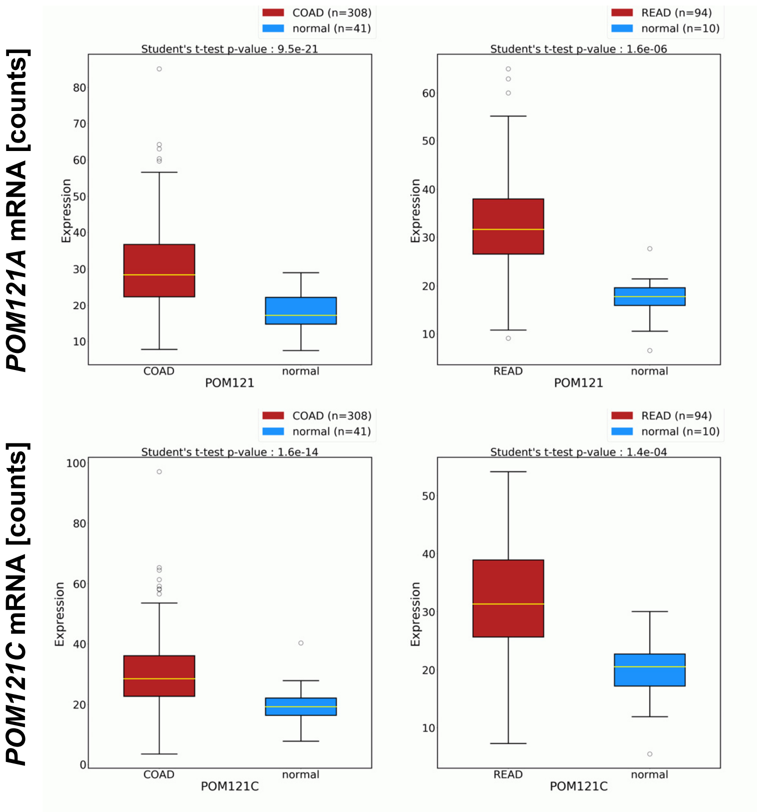

POM121A mRNA [counts]
POM121C mRNA [counts]
COAD (n=308)
normal (n=41)
READ (n=94)
normal (n=10)
Student's t-test p-value : 9.5e-21
Student's t-test p-value : 1.6e-06
Student's t-test p-value : 1.6e-14
Student's t-test p-value : 1.4e-04
Expression
COAD
READ
normal
POM121
POM121C

S4

A

CRC_PanCA

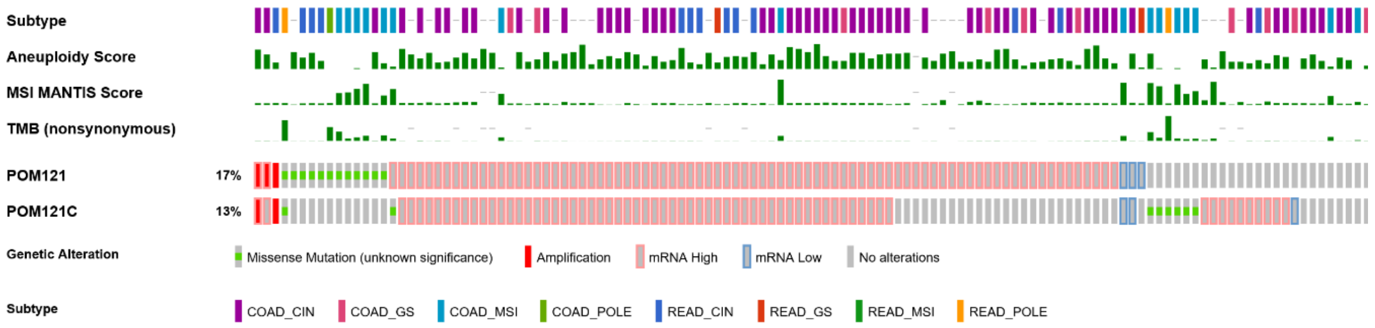

B

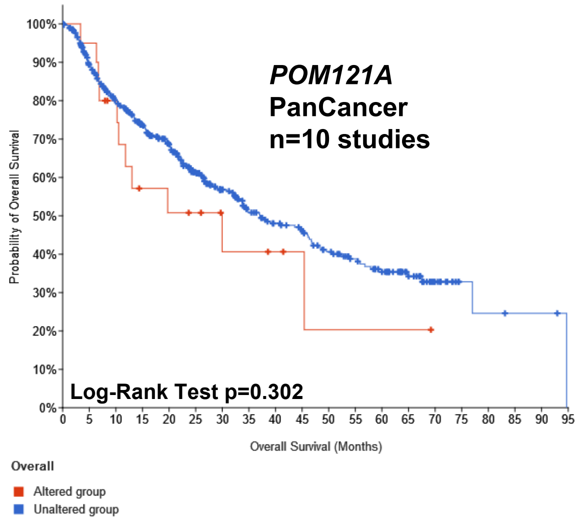

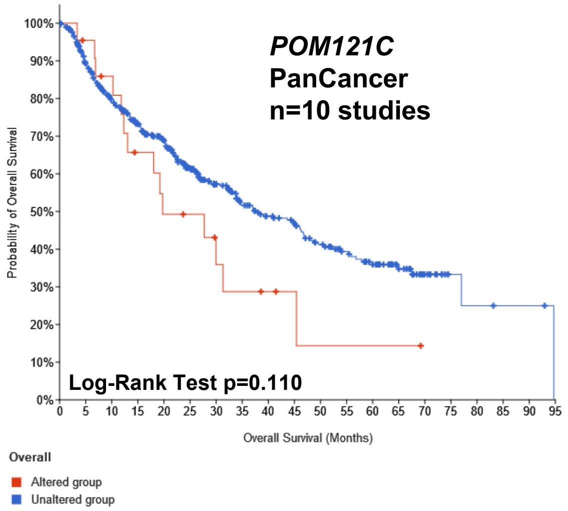

S5

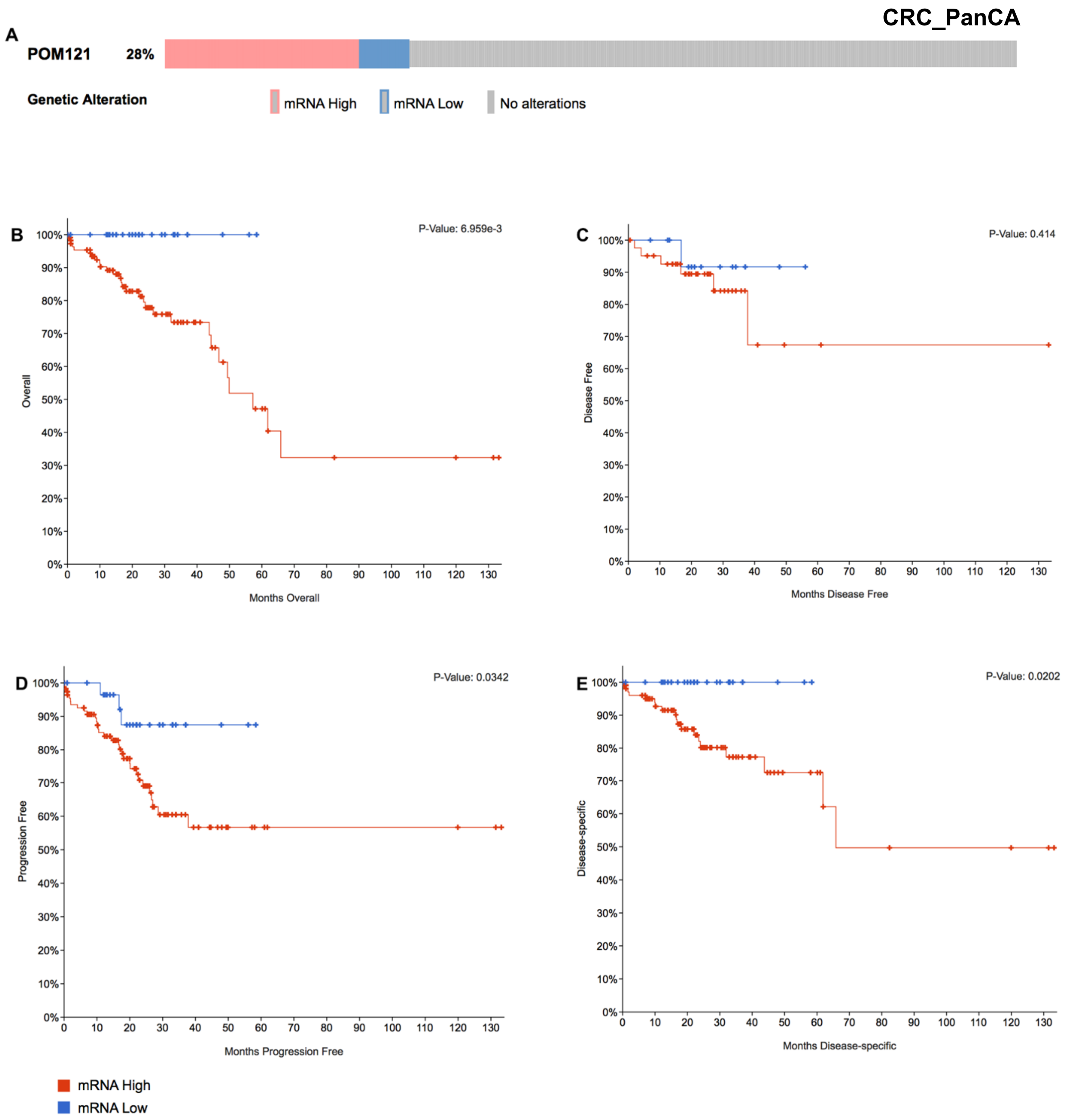
CRC_PanCA
A
POM121
28%
Genetic Alteration
mRNA High
mRNA Low
No alterations
B
P-Value: 6.959e-3
Overall
Months Overall
C
P-Value: 0.414
Disease Free
Months Disease Free
D
P-Value: 0.0342
Progression Free
Months Progression Free
E
P-Value: 0.0202
Disease-specific
Months Disease-specific
mRNA High
mRNA Low

S6

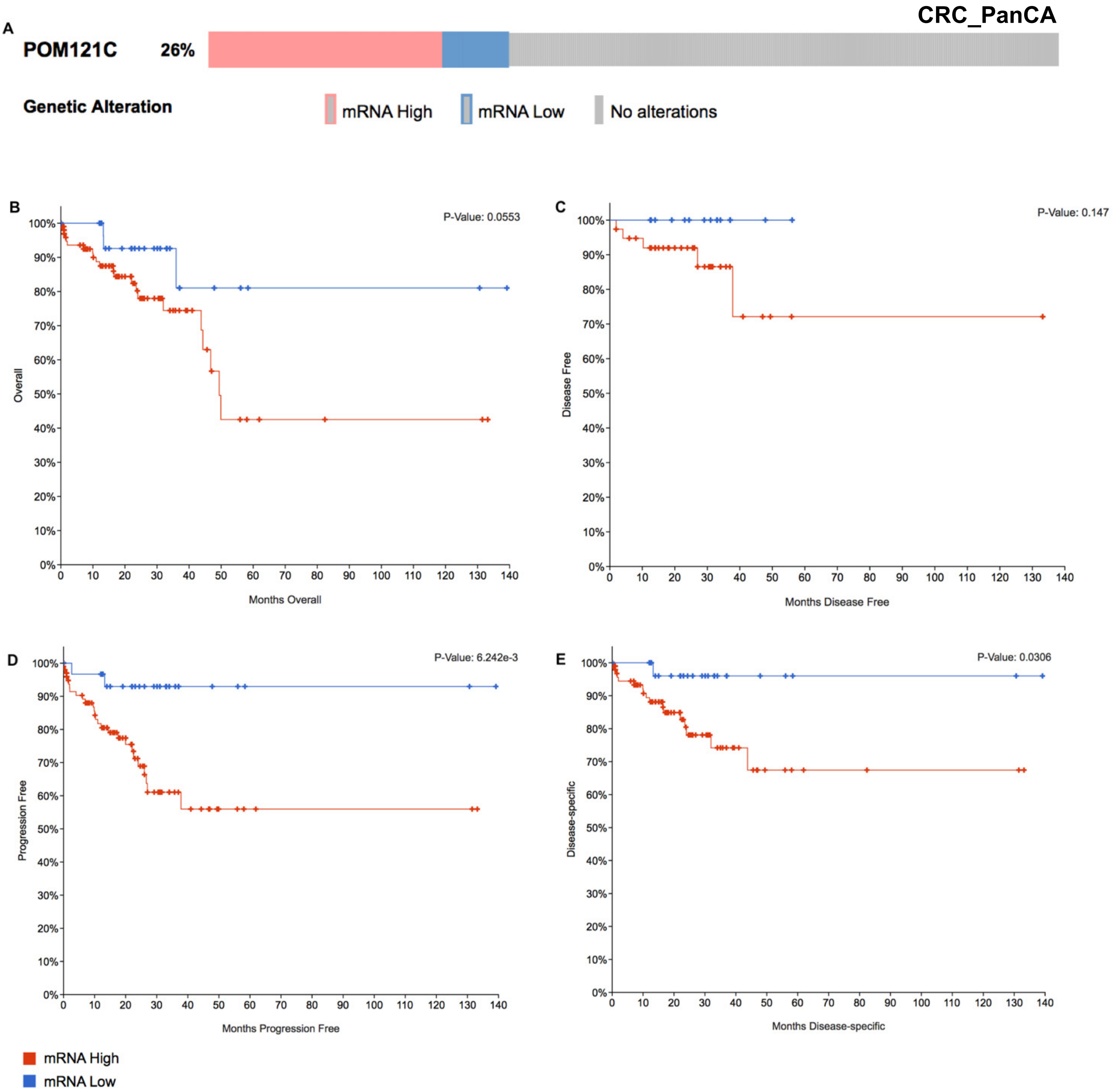

A
POM121C
26%
CRC_PanCA
Genetic Alteration
mRNA High
mRNA Low
No alterations
B
P-Value: 0.0553
Overall
Months Overall
C
P-Value: 0.147
Disease Free
Months Disease Free
D
P-Value: 6.242e-3
Progression Free
Months Progression Free
E
P-Value: 0.0306
Disease-specific
Months Disease-specific
mRNA High
mRNA Low

S7

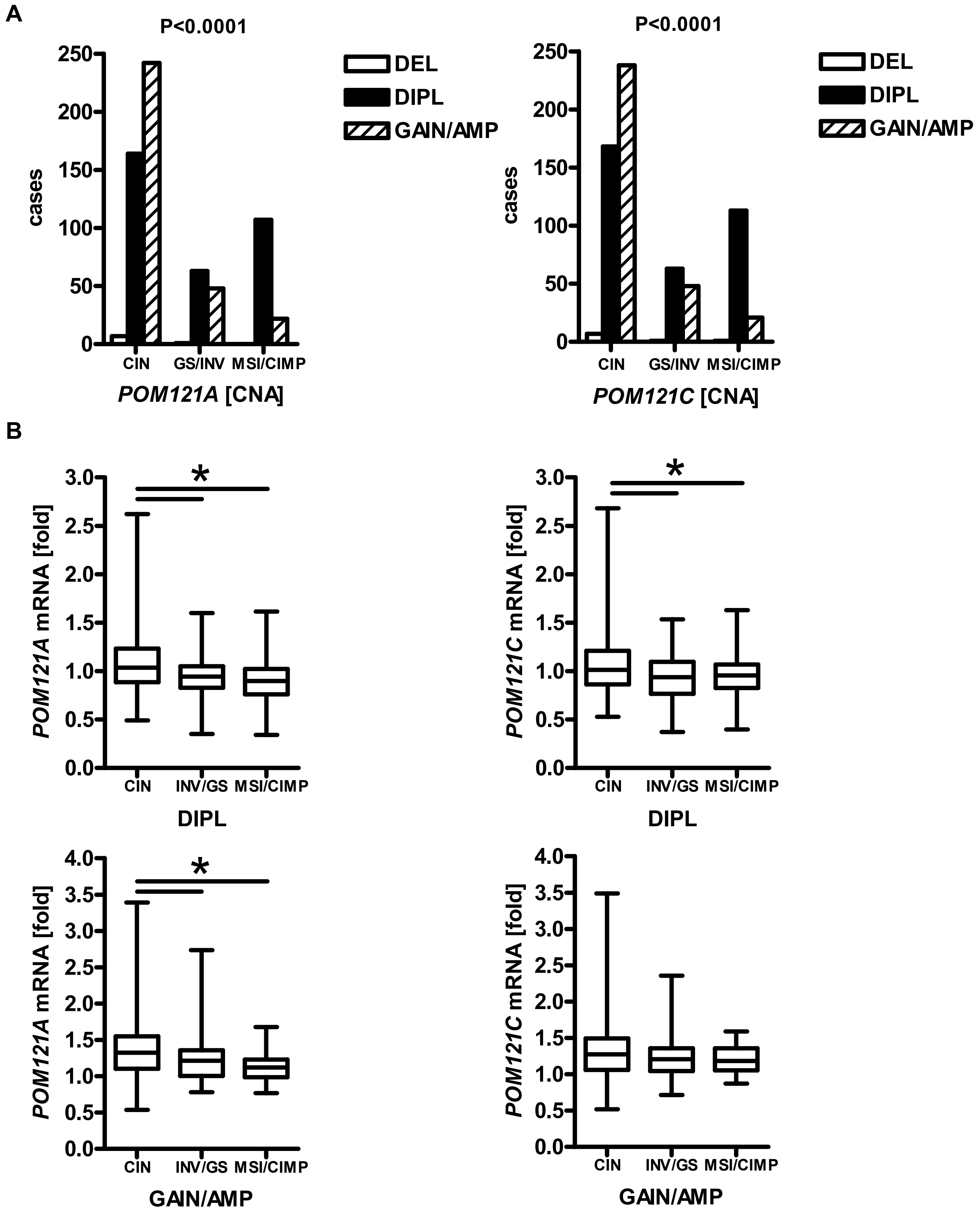
A
P<0.0001
cases
250
200
150
100
50
0
CIN
GS/INV
MSI/CIMP
POM121A [CNA]
DEL
DIPL
GAIN/AMP
P<0.0001
cases
CIN
GS/INV
MSI/CIMP
POM121C [CNA]
DEL
DIPL
GAIN/AMP
B
POM121A mRNA [fold]
3.0
2.5
2.0
1.5
1.0
0.5
0.0
*
CIN
INV/GS
MSI/CIMP
DIPL
POM121C mRNA [fold]
*
CIN
INV/GS
MSI/CIMP
DIPL
POM121A mRNA [fold]
4.0
3.5
3.0
2.5
2.0
1.5
1.0
0.5
0.0
*
CIN
INV/GS
MSI/CIMP
GAIN/AMP
POM121C mRNA [fold]
CIN
INV/GS
MSI/CIMP
GAIN/AMP

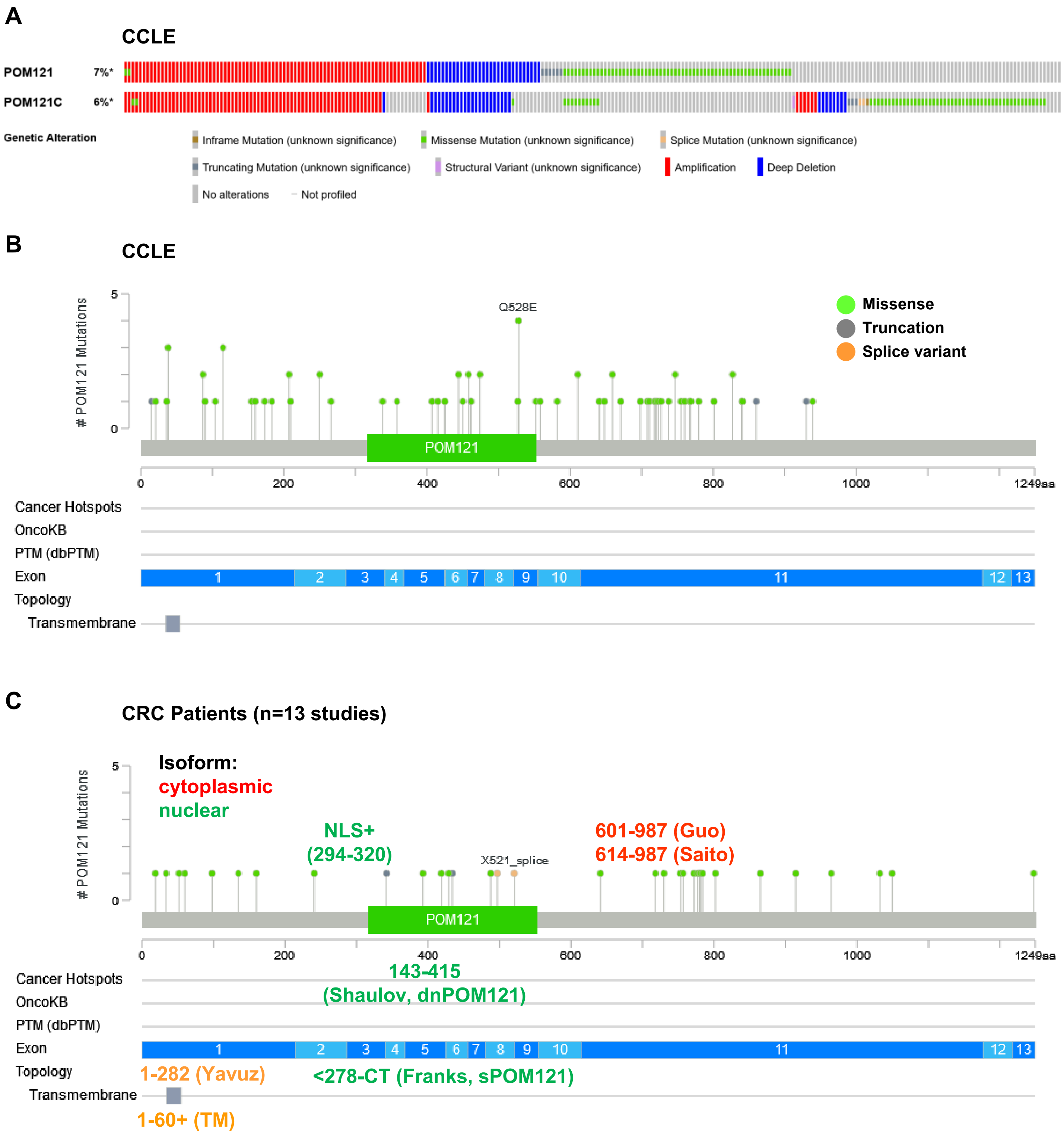

A
CCLE
POM121 7%*
POM121C 6%*
Genetic Alteration
Inframe Mutation (unknown significance)
Missense Mutation (unknown significance)
Splice Mutation (unknown significance)
Truncating Mutation (unknown significance)
Structural Variant (unknown significance)
Amplification
Deep Deletion
No alterations
Not profiled
B
CCLE
POM121 Mutations
Q528E
Missense
Truncation
Splice variant
POM121
0
200
400
600
800
1000
1249aa
Cancer Hotspots
OncoKB
PTM (dbPTM)
Exon
Topology
Transmembrane
C
CRC Patients (n=13 studies)
Isoform:
cytoplasmic
nuclear
NLS+
(294-320)
X521_splice
601-987 (Guo)
614-987 (Saito)
143-415
(Shaulov, dnPOM121)
1-282 (Yavuz)
<278-CT (Franks, sPOM121)
1-60+ (TM)

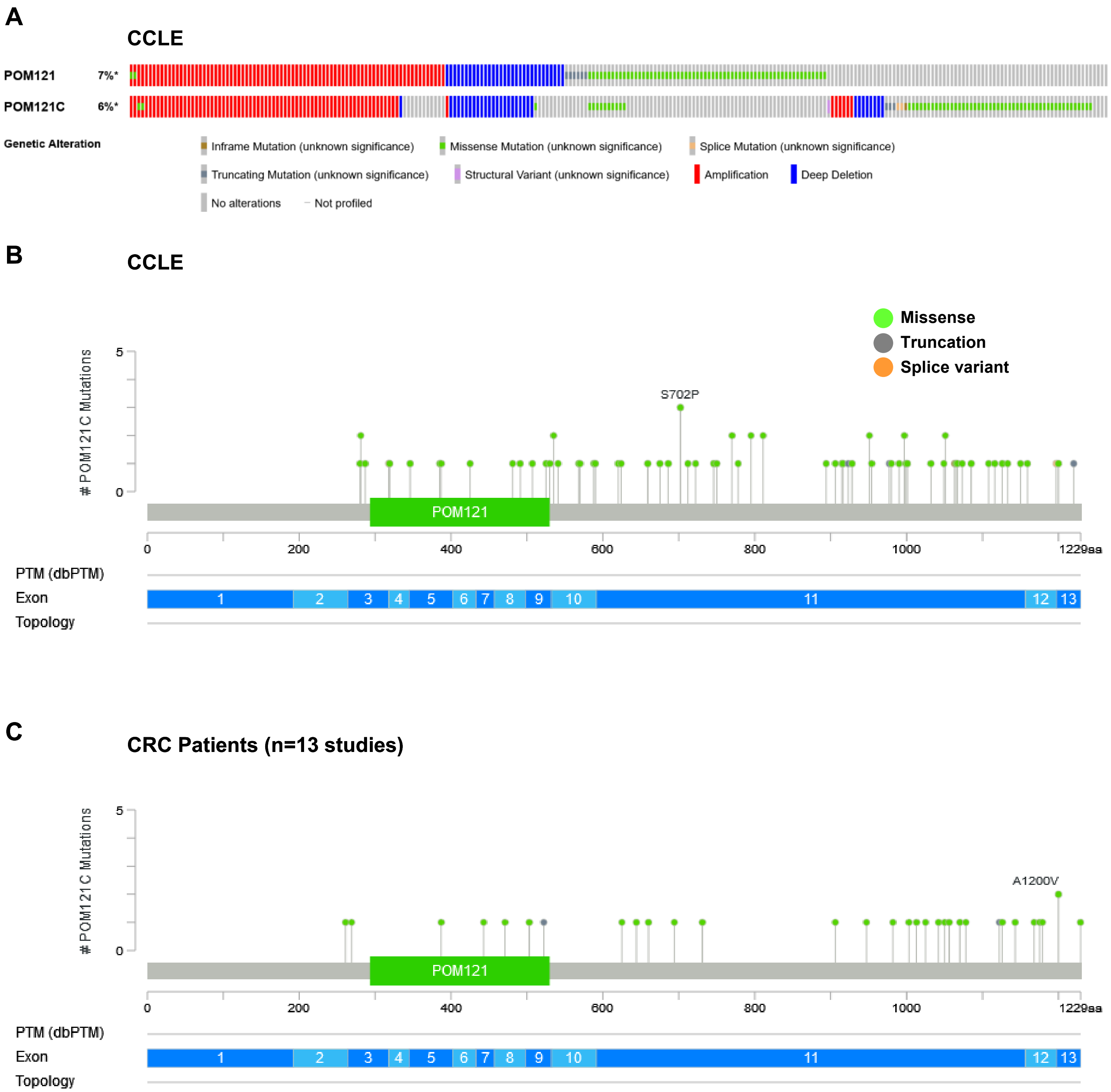

A
CCLE
POM121 7%*
POM121C 6%*
Genetic Alteration
Inframe Mutation (unknown significance)
Missense Mutation (unknown significance)
Splice Mutation (unknown significance)
Truncating Mutation (unknown significance)
Structural Variant (unknown significance)
Amplification
Deep Deletion
No alterations
Not profiled
B
CCLE
Missense
Truncation
Splice variant
POM121C Mutations
S702P
POM121
0
200
400
600
800
1000
1229aa
PTM (dbPTM)
Exon
Topology
C
CRC Patients (n=13 studies)
POM121C Mutations
A1200V
POM121
PTM (dbPTM)
Exon
Topology

S10

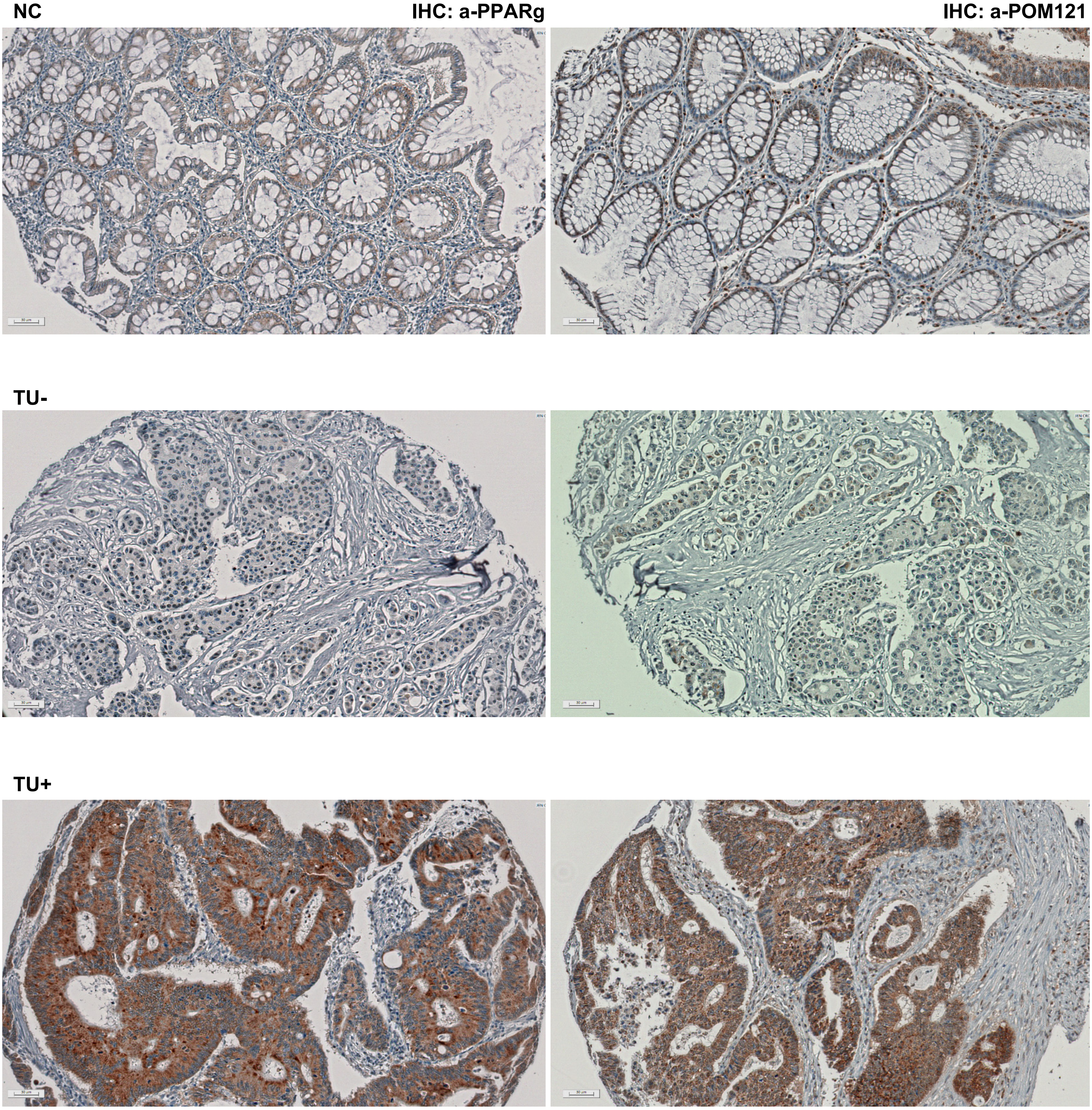

S11

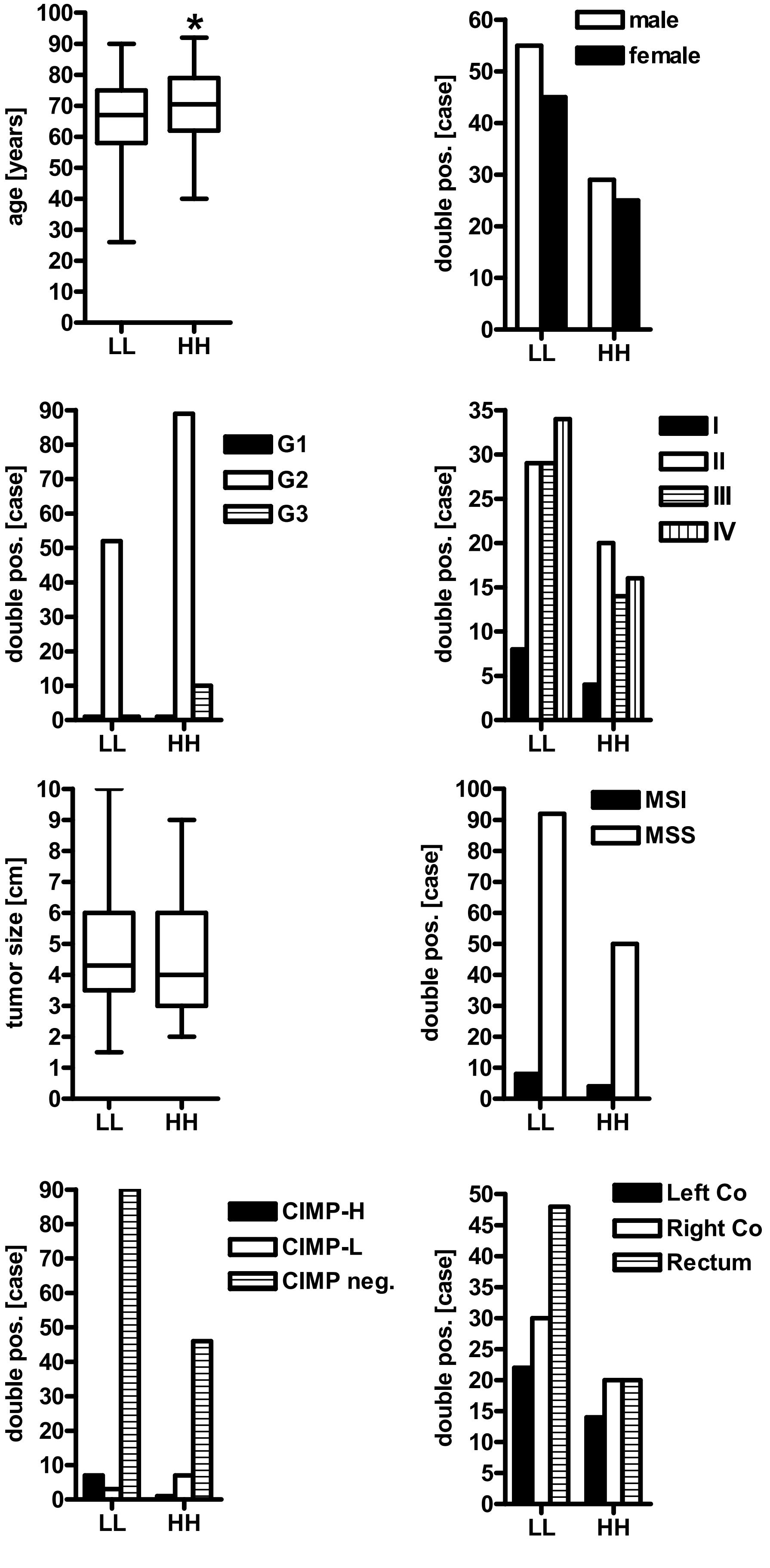
age [years]
*
LL
HH
double pos. [case]
male
female
LL
HH
double pos. [case]
G1
G2
G3
LL
HH
double pos. [case]
I
II
III
IV
LL
HH
tumor size [cm]
LL
HH
double pos. [case]
MSI
MSS
LL
HH
double pos. [case]
CIMP-H
CIMP-L
CIMP neg.
LL
HH
double pos. [case]
Left Co
Right Co
Rectum
LL
HH

S12

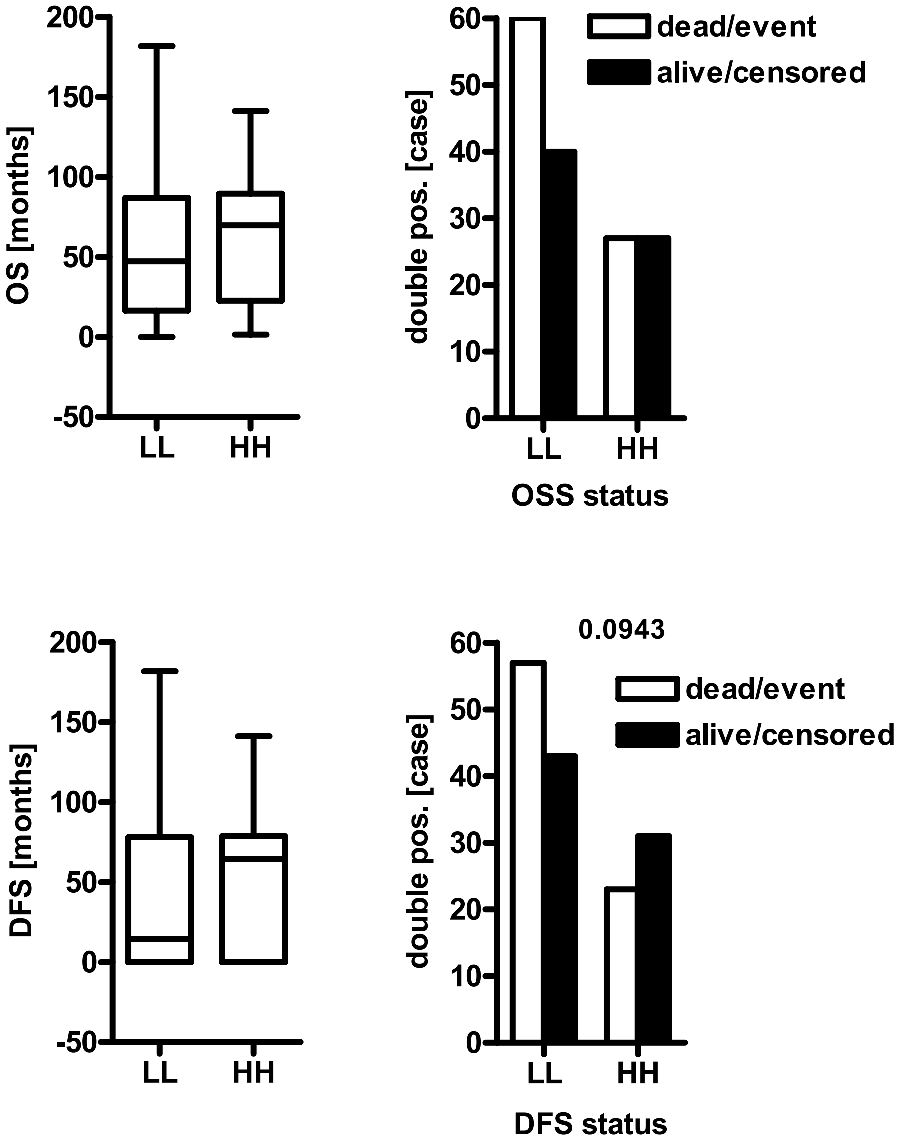

200
150
100
50
0
-50
OS [months]
LL
HH
60
50
40
30
20
10
0
double pos. [case]
dead/event
alive/censored
LL
HH
OSS status
DFS [months]
LL
HH
0.0943
double pos. [case]
dead/event
alive/censored
LL
HH
DFS status

S13

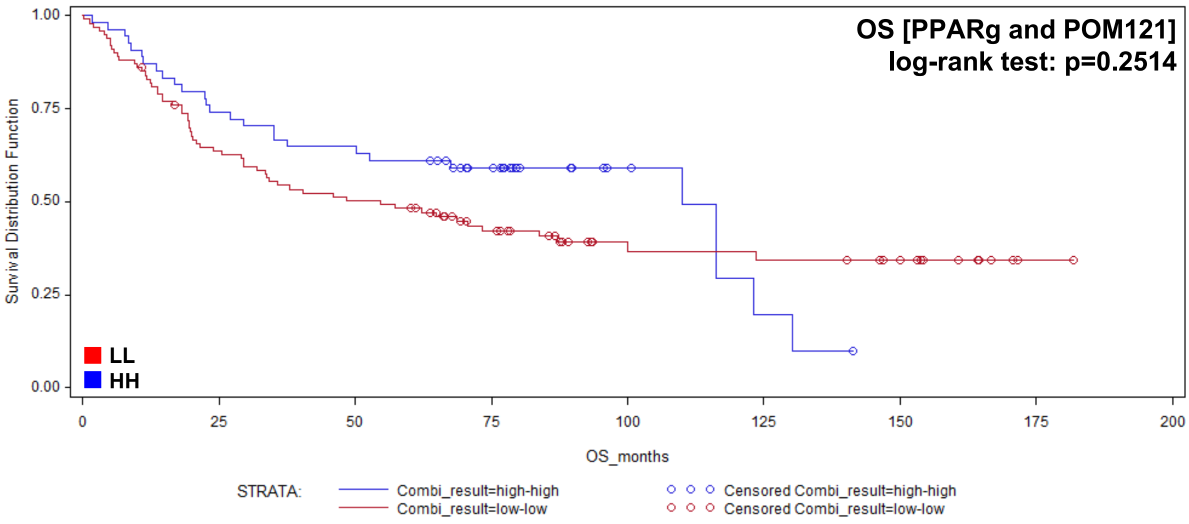

OS [PPARg and POM121]
log-rank test: p=0.2514
Survival Distribution Function
1.00
0.75
0.50
0.25
0.00
LL
HH
0
25
50
75
100
125
150
175
200
OS_months
STRATA:
Combi_result=high-high
Combi_result=low-low
Censored Combi_result=high-high
Censored Combi_result=low-low

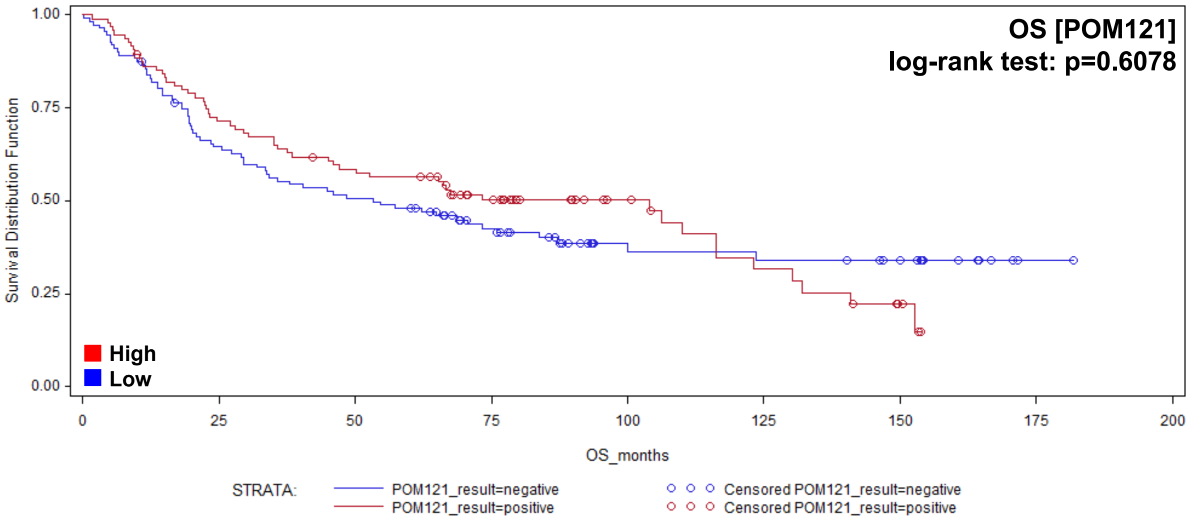

OS [POM121]
log-rank test: p=0.6078
Survival Distribution Function
1.00
0.75
0.50
0.25
0.00
High
Low
0
25
50
75
100
125
150
175
200
OS_months
STRATA:
POM121_result=negative
POM121_result=positive
Censored POM121_result=negative
Censored POM121_result=positive

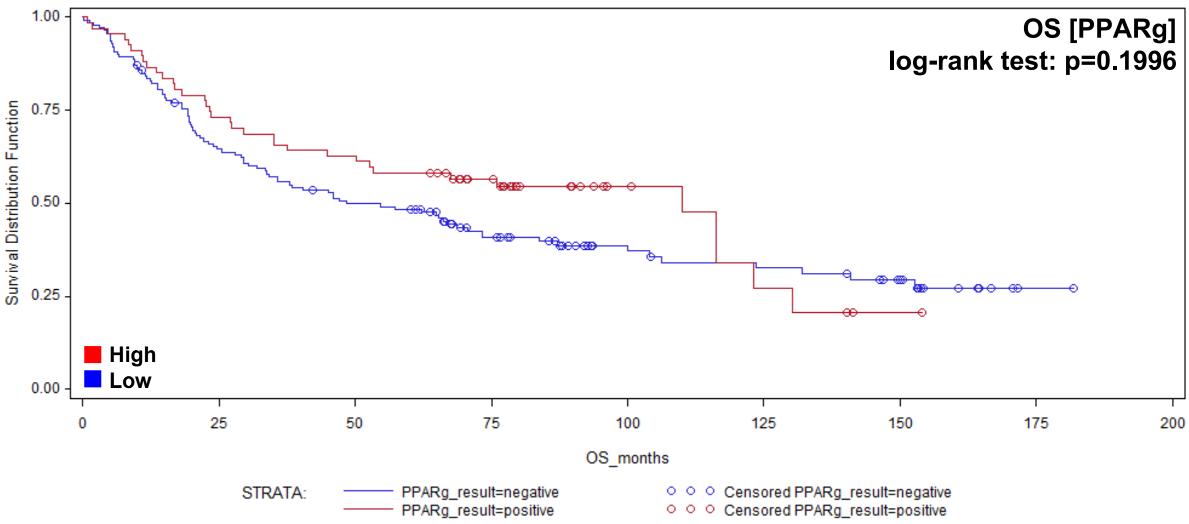

OS [PPARg]
log-rank test: p=0.1996
Survival Distribution Function
1.00
0.75
0.50
0.25
0.00
High
Low
0
25
50
75
100
125
150
175
200
OS_months
STRATA:
PPARg_result=negative
PPARg_result=positive
Censored PPARg_result=negative
Censored PPARg_result=positive

S14

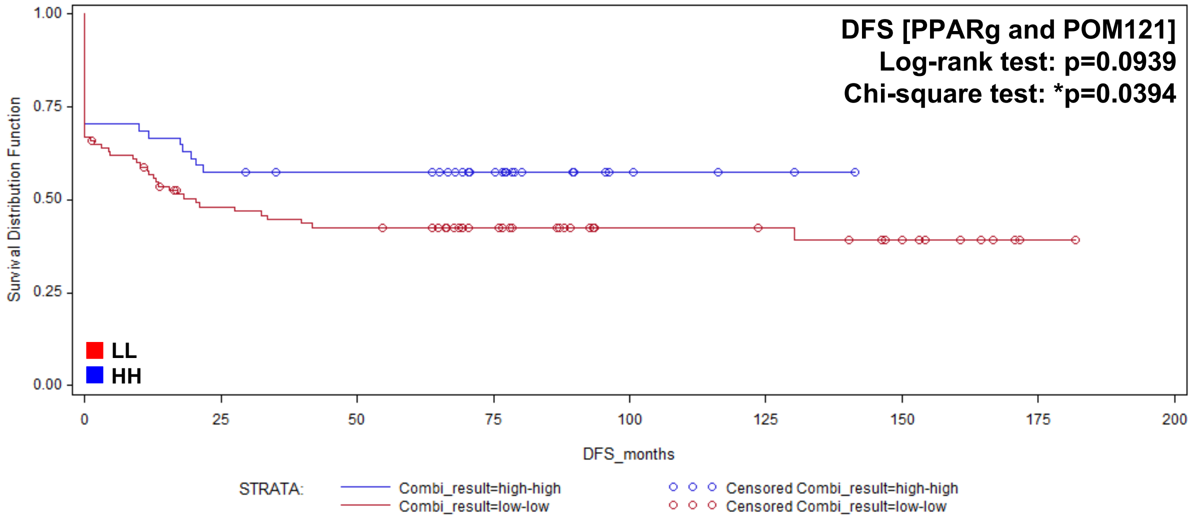

DFS [PPARg and POM121]
Log-rank test: p=0.0939
Chi-square test: *p=0.0394
Survival Distribution Function
1.00
0.75
0.50
0.25
0.00
LL
HH
0
25
50
75
100
125
150
175
200
DFS_months
STRATA:
Combi_result=high-high
Combi_result=low-low
Censored Combi_result=high-high
Censored Combi_result=low-low

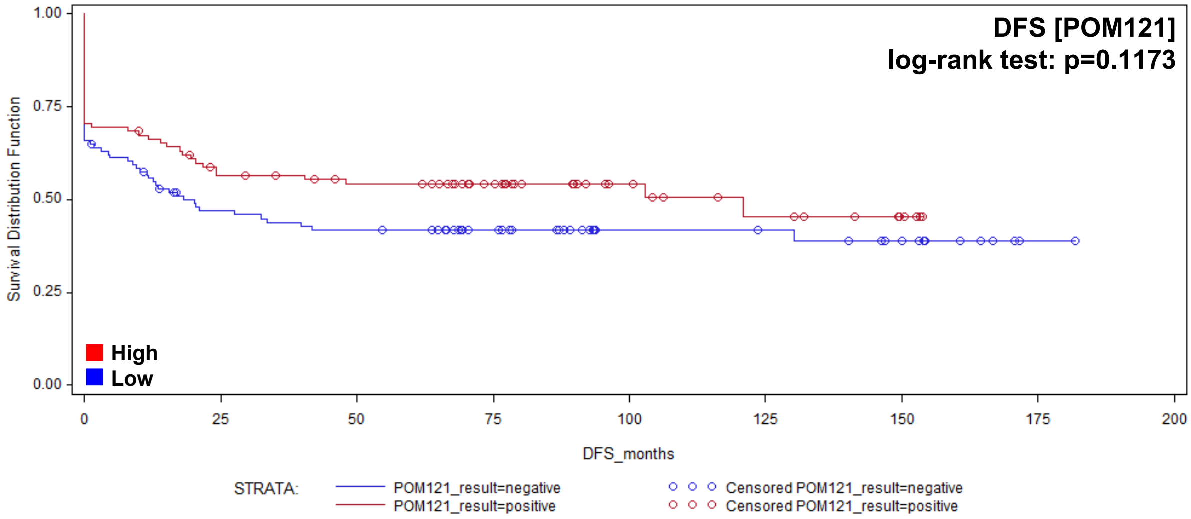

DFS [POM121]
log-rank test: p=0.1173
Survival Distribution Function
1.00
0.75
0.50
0.25
0.00
High
Low
0
25
50
75
100
125
150
175
200
DFS_months
STRATA:
POM121_result=negative
POM121_result=positive
Censored POM121_result=negative
Censored POM121_result=positive

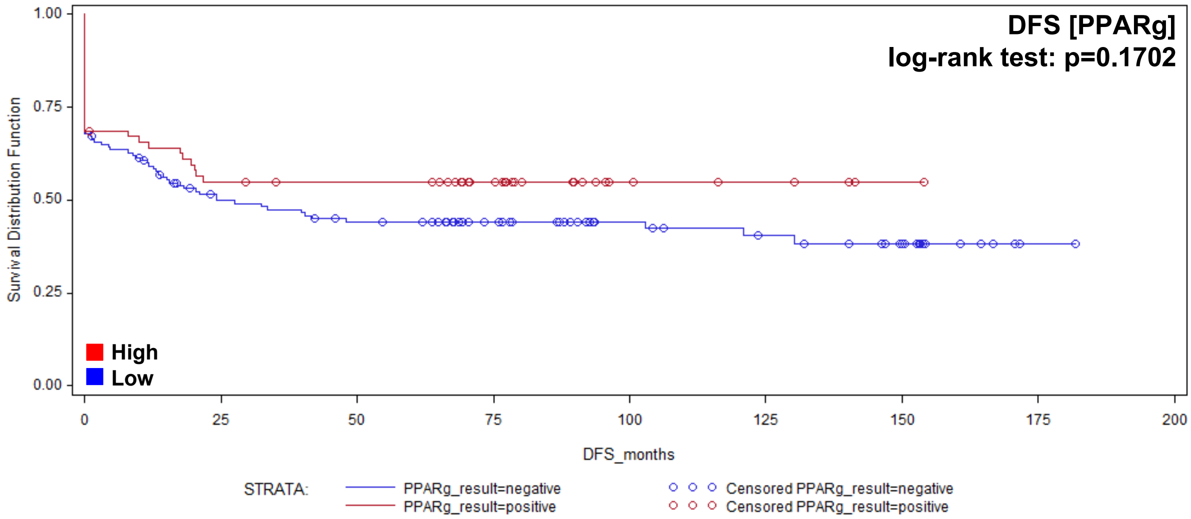

DFS [PPARg]
log-rank test: p=0.1702
Survival Distribution Function
1.00
0.75
0.50
0.25
0.00
High
Low
0
25
50
75
100
125
150
175
200
DFS_months
STRATA:
PPARg_result=negative
PPARg_result=positive
Censored PPARg_result=negative
Censored PPARg_result=positive

S15

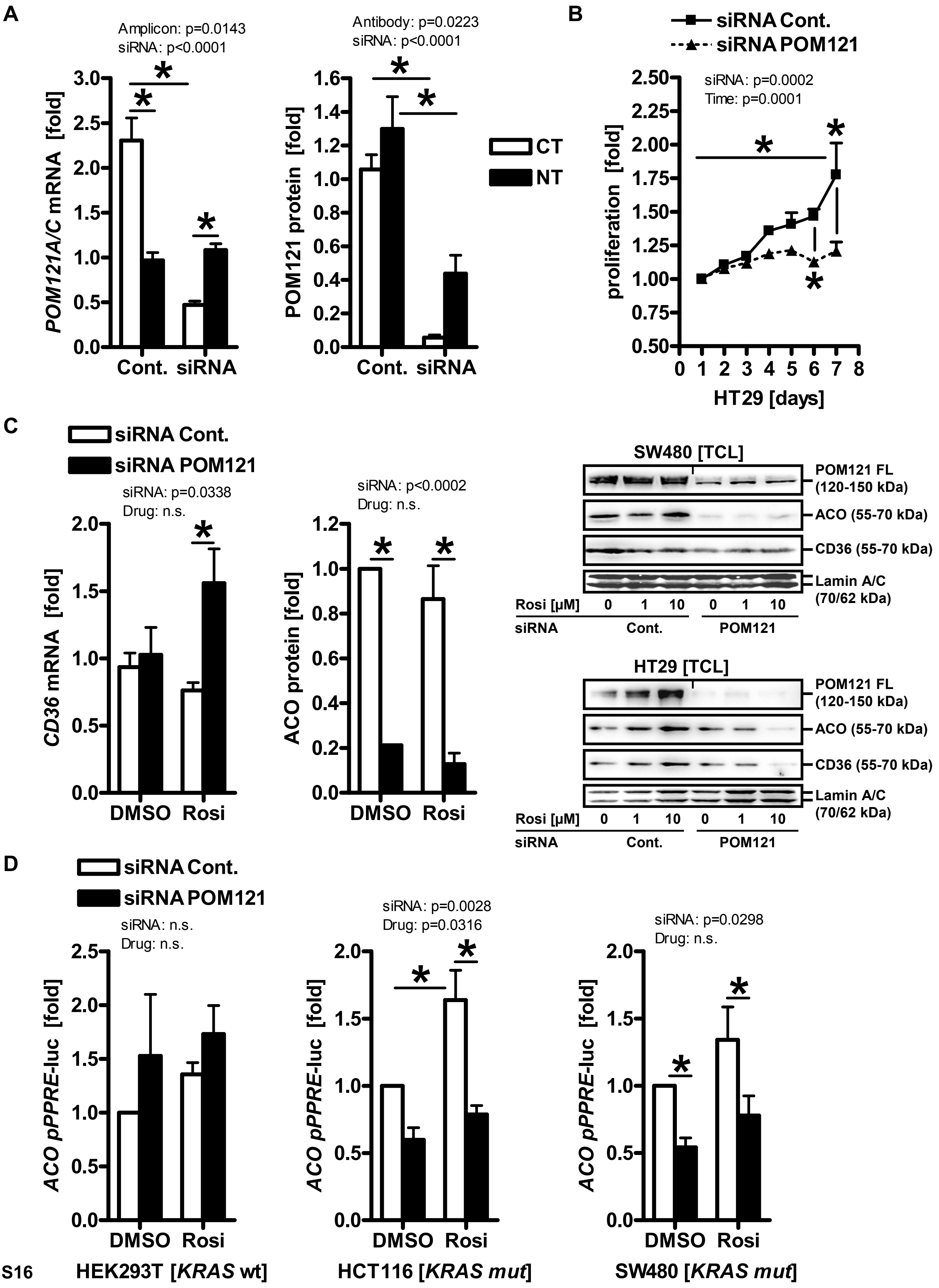
A
Amplicon: p=0.0143
siRNA: p<0.0001
POM121A/C mRNA [fold]
Cont. siRNA
Antibody: p=0.0223
siRNA: p<0.0001
POM121 protein [fold]
Cont. siRNA
CT
NT
B
siRNA Cont.
siRNA POM121
siRNA: p=0.0002
Time: p=0.0001
proliferation [fold]
HT29 [days]
C
siRNA Cont.
siRNA POM121
siRNA: p=0.0338
Drug: n.s.
CD36 mRNA [fold]
DMSO Rosi
siRNA: p<0.0002
Drug: n.s.
ACO protein [fold]
DMSO Rosi
SW480 [TCL]
POM121 FL (120-150 kDa)
ACO (55-70 kDa)
CD36 (55-70 kDa)
Lamin A/C (70/62 kDa)
Rosi [µM] 0 1 10 0 1 10
siRNA Cont. POM121
HT29 [TCL]
POM121 FL (120-150 kDa)
ACO (55-70 kDa)
CD36 (55-70 kDa)
Lamin A/C (70/62 kDa)
Rosi [µM] 0 1 10 0 1 10
siRNA Cont. POM121
D
siRNA Cont.
siRNA POM121
siRNA: n.s.
Drug: n.s.
ACO pPPRE-luc [fold]
DMSO Rosi
HEK293T [KRAS wt]
siRNA: p=0.0028
Drug: p=0.0316
ACO pPPRE-luc [fold]
DMSO Rosi
HCT116 [KRAS mut]
siRNA: p=0.0298
Drug: n.s.
ACO pPPRE-luc [fold]
DMSO Rosi
SW480 [KRAS mut]

A

Jpred4

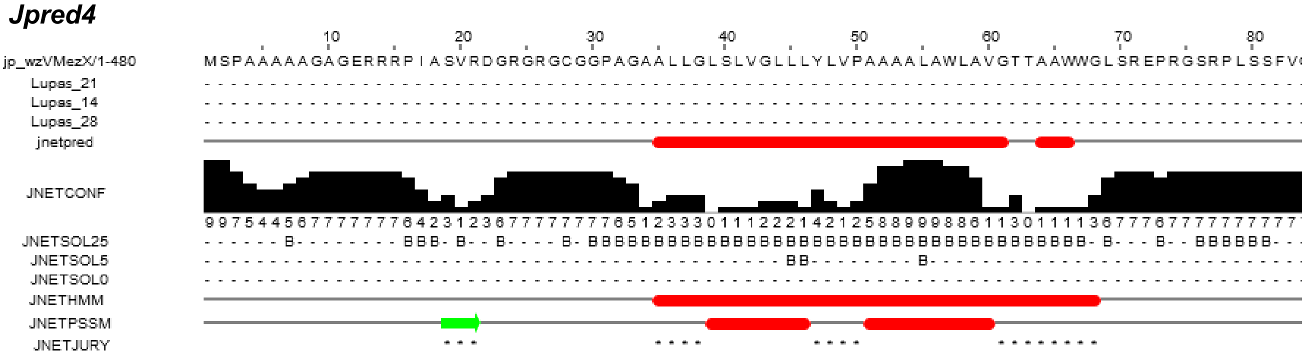

B ***pepwindow octanol***

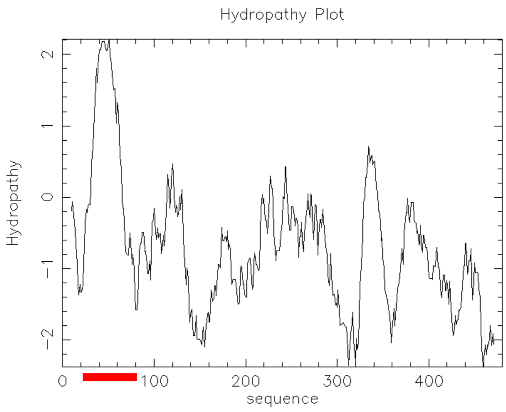

C

Phyre2

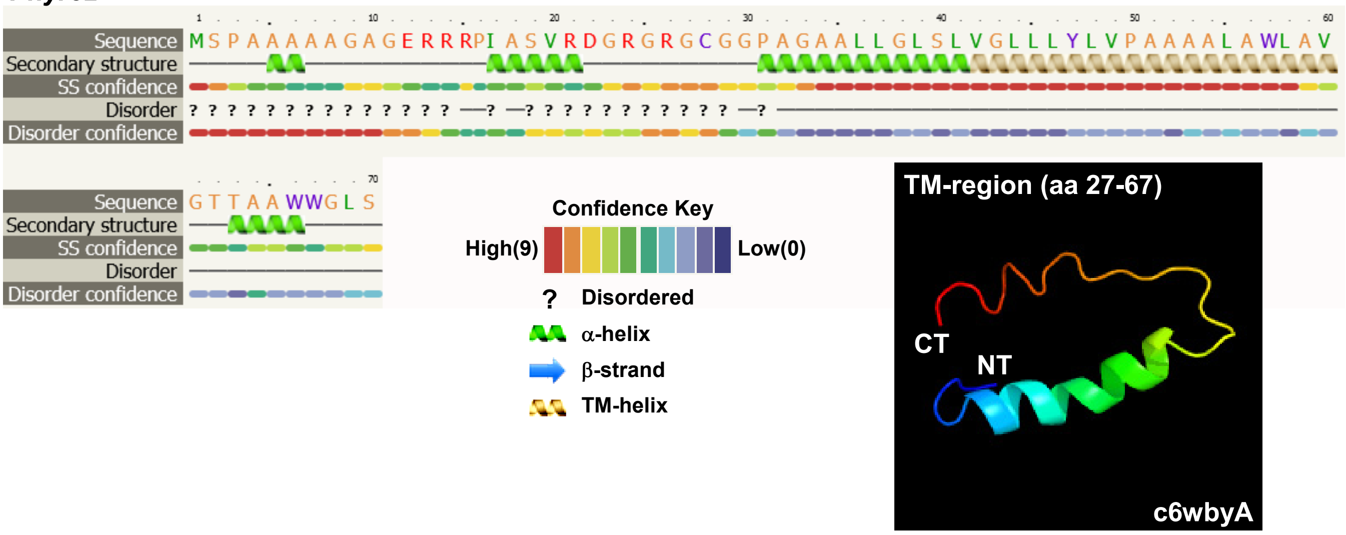

D

EzMol

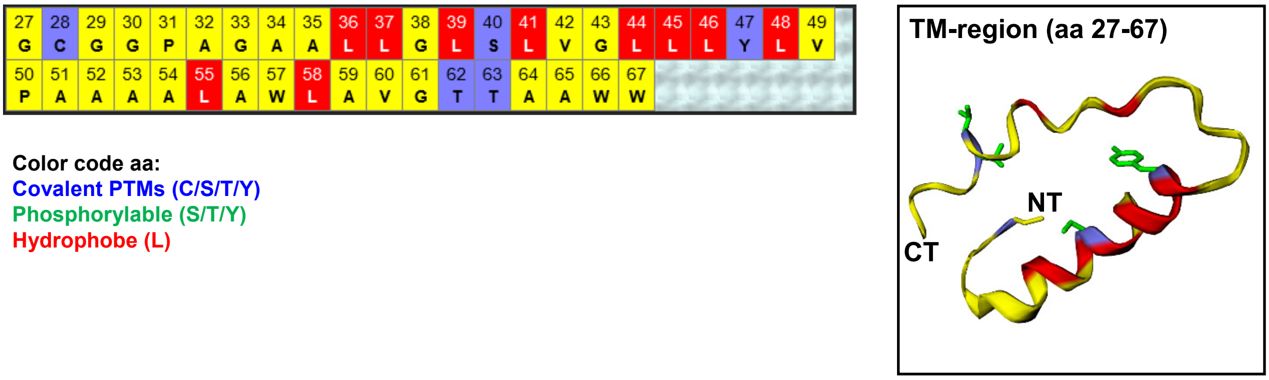

Color code aa:
Covalent PTMs (C/S/T/Y)
Phosphorylable (S/T/Y)
Hydrophobe (L)

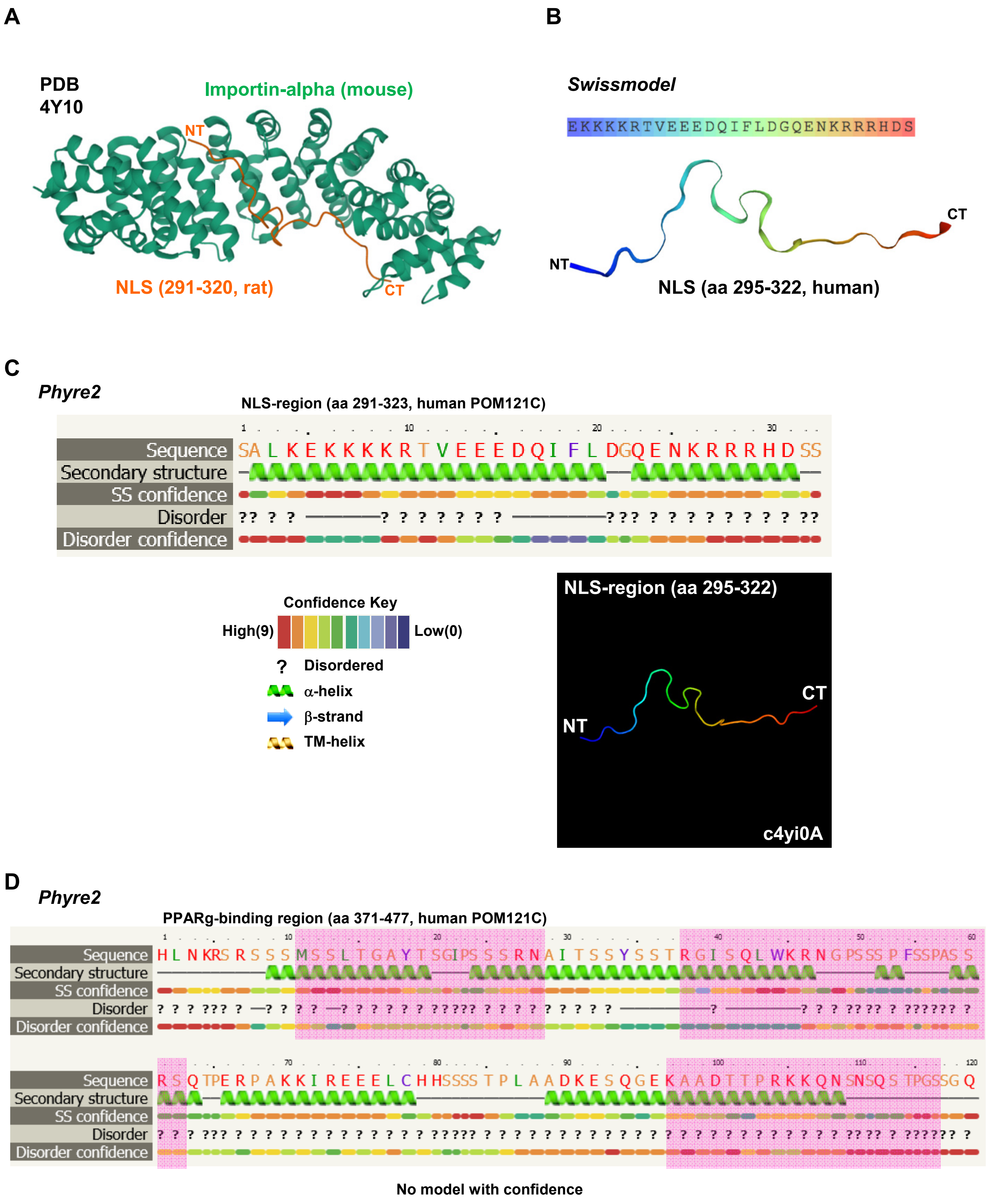
A
PDB
4Y10
Importin-alpha (mouse)
NT
CT
NLS (291-320, rat)
B
Swissmodel
EKKKKRTVEEEDQIFLDGQENKRRRHDS
NT
CT
NLS (aa 295-322, human)
C
Phyre2
NLS-region (aa 291-323, human POM121C)
Sequence
Secondary structure
SS confidence
Disorder
Disorder confidence
SALKEKKKKRTVEEEDQIFLDGQENKRRRHDSS
Confidence Key
High(9)
Low(0)
? Disordered
α-helix
β-strand
TM-helix
NLS-region (aa 295-322)
NT
CT
c4yi0A
D
Phyre2
PPARg-binding region (aa 371-477, human POM121C)
Sequence
Secondary structure
SS confidence
Disorder
Disorder confidence
No model with confidence
