# Supplementary material for: Nuclear pore protein POM121 regulates subcellular localization and transcriptional activity of PPARγ
Source: Cell Death Dis. 2024 Jan 4;15(1):7. doi: 10.1038/s41419-023-06371-1 (PMC10766976; doi:10.1038/s41419-023-06371-1)
Supplement: Supplementary file 3 — Supplementary tables R2 [file 41419_2023_6371_MOESM3_ESM.docx]

**Supplementary Tables**

| **Table S1 Antibodies**  Legend: N = N-terminal; C = C-terminal epitope of full-length (FL) protein. | | | |
| --- | --- | --- | --- |
| **Protein** | **Clone (Add-On)** | **Cat. No.** | **Company** |
| PPARγ (N) | Rabbit | sc-7196 (H100, discontinued) | Santa Cruz Bio. |
| PPARγ (N) | Rabbit | 2435 | Cell Signaling |
| PPARγ (C) | Mouse | sc-7273 (E8) | Santa Cruz Bio. |
| PPARγ (C) | Mouse | 95128 | Cell Signaling |
| POM121 (N) | Rabbit | PA5-85161 | Thermofisher |
| POM121 (C) | Rabbit | ab137938 | Abcam |
| ACO | Rabbit | PA5-76341 | Thermofisher |
| CD36 | Rabbit | PA1-16813 | Thermofisher |
| P21 (WAF1/CIP1) | Rabbit | 2947 | Cell Signaling |
| HSP90 | Mouse | sc-13119 | Santa Cruz Bio. |
| β-Actin | Mouse | sc-69879 | Santa Cruz Bio. |
| Lamin A/C | Rabbit | sc-20681 | Santa Cruz Bio. |

| **Table S2 Oligonucleotides**  Legend: I = internal; N = N-terminal; C = C-terminal amplicon of full-length (FL) cDNA. | | | |
| --- | --- | --- | --- |
| **Cloning** | **Sequence (5' > 3')** | **Amplicon (bp)** | **Gene** |
| *5-sgRNA-POM121A/C* | CACCGGTGATAACCATTCCAGCACA | 25 | *POM121A/C* |
| *3-sgRNA-POM121A/C* | AAACTGTGCTGGAATGGTTATCACC |  |  |
| **RT-qPCR** | **Sequence (5' > 3')** | **Amplicon (bp)** | **Gene** |
| *5-P21* | gacaccactggagggtgact | 172 | *CIP1/WAF1* |
| *3-P21* | caggtccacatggtcttcct |  |  |
| *5-PPARG* | Gctggcctccttgatgaata | 113 | *PPARG* |
| *3-PPARG* | Ttgggctccataaagtcacc |  |  |
| *5-POM121A/C(I)* | aaggctgcagatacaacccc | 164 | *POM121A/C* |
| *3-POM121A/C(I)* | tcggcagtgatcgaatagcc |  |  |
| *5-POM121(N)* | gatagcgagtgtcagggacg | 203 | *POM121A/C* |
| *3-POM121(N)* | gaacgaaggaggacaagggg |  |  |
| *5-POM121(C)* | cttaggtcagaacgccctgg | 210 | *POM121A/C* |
| *3-POM121(C)* | ggtccaacaccaacaaagcc |  |  |
| *5-Cas9* | AACCTATGCCCACCTGTTCG | 129 | *cas9* |
| *3-Cas9* | CAGTCCGGCAAGACAATCCT |  |  |
| *5-ACO* | ctgtgaggcaccagtctgaa | 186 | *ACOX1* |
| *3-ACO* | gttcactcaggtccccttga |  |  |
| *5-CD36* | agatgcagcctcatttccac | 150 | *CD36* |
| *3-CD36* | gccttggatggaagaacaaa |  |  |
| *5-ACTB* | GTCTTCCCCTCCATCGTG | 113 | *ACTB* |
| *3-ACTB* | AGGGTGAGGATGCCTCTCTT |  |  |
| *5-GAPDH* | GGAGCGAGATCCCTCCAAAAT | 197 | *GAPDH* |
| *3-GAPDH* | GCGTGTTGTCATACTTCTCATGG |  |  |
| *5-B2M* | tgctgtctccatgtttgatgtatct | 86 | *B2M* |
| *3-B2M* | tctctgctccccacctctaagt |  |  |

| **Table S3 Peptides ^#^ identified by MALDI-MS upon IP with PPARγ Ab**  ***** Mascot total ion score by GPS Explorer 2 software  (Applied Biosystems/Thermofisher Scientific). | | | | | |
| --- | --- | --- | --- | --- | --- |
| **Accession**  **(UniProt)** | **Mass (Dalton)** | **Score** * | **Description** | | **Species** |
| C9JFL1 | 11.368 | 30 | POM121 transmembrane  nucleoporin C | | C9JFL1_HUMAN |
| Q9H5C8 | 51.960 | 33 | cytoplasmic IQ domain-containing protein G / dynein regulatory complex subunit 9 | | Q9H5C8_HUMAN |
| P13645 | 59.020 | 78 | keratin, type I  cytoskeletal 10 | | K1C10_HUMAN |
| UPI00017BCE7F | 58.994 | 78 | keratin, type I  cytoskeletal 10 | | UPI00017BCE7F |
| **^#^** Location of peptides identified by MALDI-MS in the coding sequence (CDS)  of human POM121C protein aligned with BlastP software (NCBI). | | | | | |
| **Mass (Dalton)** | **CDS location** | | | **Peptide** | |
| 831,449  858,4559  1233,5585  1569,7635  1620,7492 | Internal (C-terminal of NLS)  Internal (C-terminal of NLS)  Internal (& **S**erine-rich)  Internal (& **S**erine-rich)  Internal (& **S**erine-rich) | | | R.GISQLWK.R + Deamidated (NQ)  K.AADTTPRK.K  K.KQN**S**N**S**Q**S**TPG**S**.-  M.**SS**LTGAYT**S**GIP**SSS**R.N  K.RNGP**SSS**PF**SS**PA**SS**R.**S** + Deamidated (NQ) | |

| **Table S4 POM121A/C protein isoforms**  Legend: aa amino acid; TM transmembrane domain; NLS nuclear localization sequence; FL full-length; NT N-Terminus; CT C-Terminus;  * experimental mutant; $ endogenous variant. | | | |
| --- | --- | --- | --- |
| **POM121 [aa]** | **TM** | **NLS** | **UniProt / Ref.** |
| 1-1249 FL ^$^ | + | + | P121A_HUMAN |
| 1-1229 FL ^$^ | + | + | P121C_HUMAN |
| 1-107 NT ^$^ | + | - | C9JFL1_HUMAN |
| 1-282 NT * | + | - | *Yavuz et al.* |
| 143-415 NT * | - | + | *Shaulov et al.* |
| <278-CT ^$^ | - | + | *Franks et al.* |
| 601-CT * | - | - | *Guo et al.* |
| 614-CT ^$^ | - | - | *Saito et al.* |

| **Table S5**  **Association of POM121/PPARγ protein expression with clinical factors in CRC patients *^a^***  *^a^* IHC with PPARγ and POM121 Abs on CRC TMAs followed by dichotome analysis of staining scores (negative = score 0/1 *vs.* positive = score 2/3). Note that only double positive *vs.* double negative samples were compared; single stained samples were excluded from the analysis. Abbreviations: G=tumor grade; pTNM-categories: T=local tumor growth, N=nodal spread, M=distant metastasis, UICC=tumor staging, CIMP=CpG island methylator phenotype, MSI/MSS=microsatellite instability/stability, wt=wildtype, mut=mutated. | | | | |
| --- | --- | --- | --- | --- |
| **N=154** | **PPARγ+**  **POM121+** | **PPARγ-**  **POM121-** | **PPARγ+**  **POM121+ (%)** | **PPARγ-**  **POM121- (%)** |
| **Gender** |  |  |  |  |
| Female | 25 | 45 | 46 | 45 |
| Male | 29 | 55 | 54 | 55 |
| p=1.0000 (Fisher Exact Test) | | | | |
| **Age** |  |  |  |  |
| Cases | 54 | 100 |  |  |
| Mean ± SD | 70.09±12.05 | 65.80±12.10 |  |  |
| ***p=0.0375** (t-test Procedure) | | | | |
| **Localization** |  |  |  |  |
| Right Colon | 20 | 30 | 37 | 30 |
| Left Colon | 14 | 22 | 26 | 22 |
| Rectum | 20 | 48 | 37 | 48 |
| p=0.2207 (Mantel-Haenszel Chi Square Test) | | | | |
| **Size [cm]** |  |  |  |  |
| Cases | 54 | 100 |  |  |
| Mean ± SD | 4.51±1.73 | 4.52±1.69 |  |  |
| p=0.7350 (Kruskal-Wallis Test) | | | | |
| **Grade** |  |  |  |  |
| G1 | 1 | 1 | 2 | 1 |
| G2 | 52 | 89 | 96 | 89 |
| G3 | 1 | 10 | 2 | 10 |
| **p=0.0611 (**Cochran-Armitage Trend Test) | | | | |
| **UICC stage** |  |  |  |  |
| I | 4 | 8 | 7 | 8 |
| II | 20 | 29 | 37 | 29 |
| III | 14 | 29 | 26 | 29 |
| IV | 16 | 34 | 30 | 34 |
| p=0.4913 (Cochran-Armitage Trend Test) | | | | |
| **CIMP** |  |  |  |  |
| Negative | 46 | 90 | 85 | 90 |
| Low | 7 | 3 | 13 | 3 |
| High | 1 | 7 | 2 | 7 |
| p=0.9681 (Cochran-Armitage Trend Test) | | | | |
| **MSI** |  |  |  |  |
| MSS+ | 50 | 92 | 93 | 92 |
| MSI+ | 4 | 8 | 7 | 8 |
| p=0.2459 (Fisher Exact Test) | | | | |
| ***KRAS* mutation** | **wt** | | **mut** | |
| **PPARγ localization** | **Nucleus** | **Cytoplasm** | **Nucleus** | **Cytoplasm** |
| **POM121+** | 11 | 33 | 8 | 55 |
| **POM121-** | 12 | 35 | 5 | 18 |
| ***p=0.0063** (Chi-Square Test) | | | | |
|  | | | | |

| **Table S6**  **Association of POM121/PPARγ protein expression with survival in CRC patients *^a^***  *^a^* IHC with PPARγ and POM121 Abs on CRC TMAs followed by dichotome analysis of staining scores  (negative = score 0/1 *vs.* positive = score 2/3). Abbreviations: OSS = overall survival, DFS = disease-free survival. | | | | |
| --- | --- | --- | --- | --- |
| **Multiple Logistic Regression (n=154)** | | | | |
| *Parameter* | *Odds Ratio* | *95% CI* | *Chi Square Test* |  |
| Age | 1.028 | [0.998-1.059] | **p=0.0678** |  |
| Grade | 0.291 | [0.059-1.431] | p=0.1288 |  |
| **Coexpression (n=205)** | | | | |
| *Protein* | **PPARγ-** | **PPARγ+** | *McNemar Test* |  |
| **POM121-** | 100 | 11 | ***p<0.0001** |  |
| **POM121+** | 40 | 54 |  |  |
| **OSS status (n=154)** | | | | |
| *Protein* | *Censored/Alive* | *Event/Death* | *Fisher Exact Test* |  |
| **PPARγ+ POM121+** | 27 [50 %] | 27 [50 %] | p=0.2393 |  |
| **PPARγ- POM121-** | 40 [40 %] | 60 [60 %] |  |  |
| **DFS status (n=154)** |  |  |  |  |
| *Protein* | *Censored/Alive* | *Event/Death* | *Fisher Exact Test* |  |
| **PPARγ+ POM121+** | 31 [57 %] | 23 [43%] | **p=0.0943** |  |
| **PPARγ- POM121-** | 43 [43 %] | 57 [57 %] |  |  |
| **Prognosis (n=154)** | | | | |
|  | **OSS** | | **DFS** | |
| *Protein* | *Log Rank Test* | *Chi Square Test* | *Log Rank Test* | *Chi Square Test* |
| **POM121** | p=0.6078 | p=0.6089 | p=0.1173 | p=0.1604 |
| **PPARγ** | p=0.1996 | p=0.2009 | p=0.1702 | p=0.2184 |
| **POM121 & PPARγ** | p=0.2514 | p=0.2529 | **p=0.0939** | ***p=0.0394** |

| **Table S7 Predicted interactome of POM121**  Protein candidates were identified (total >100) and selected by biological relevance using BioGRID for POM121 [UniProt ID: Q96HA1 (P121A_HUMAN)] and IntAct/STRING for POM121C [UniProt ID: A8CG34 (P121C_HUMAN)]. Legend: * interactors for both POM121 proteins; § interactors for POM121C protein only. | | |
| --- | --- | --- |
| **Gene** | **UniProt ID** | **Full Name** |
| ***Metabolism*** | | |
| esr2 § | Q92731 | estrogen receptor beta |
| TRIP6 | Q15654 | thyroid hormone receptor interactor 6 |
| TRHDE | Q9UKU6 | thyrotropin-releasing hormone degrading enzyme 1 |
| TSHB | P01222 | TSH-B, TSH-BETA thyroid stimulating hormone, beta |
| BSCL2 | Q96G97 | Berardinelli-Seip congenital lipodystrophy 2 (seipin) |
| SCAP | Q12770 | SREBF chaperone |
| ***Cancer & Immunity*** | | |
| APC * | P25054 | adenomatous polyposis coli |
| BRCA1 | P38398 | breast cancer 1, early onset |
| CTLA4 | P16410 | cytotoxic T-lymphocyte-associated protein |
| NFKBIA | P25963 | nuclear factor of kappa light polypeptide gene enhancer in B-cells inhibitor alpha |
| STAT3 | P40763 | signal transducer and activator of transcription 3 |
| sta5a § | P42229 | signal transducer and activator of transcription 5A |
| pcnA § | P12004 | proliferating cell nuclear antigen |

| **Table S8 *POM121A/C* mRNA up-regulation in GI cancers**  Data were retrieved from *Oncomine*®. Legend: n patient case numbers; NT non-tumor (normal colon tissue); TU tumor.  Significance thresholds: p<1.0e-4, fold change >2, gene rank top 10%. | | | | | |
| --- | --- | --- | --- | --- | --- |
| **Up-regulation in TU [*POM121A/C* mRNA]** | | | | | |
| **Data set** | **Σn** | **NT** | **n** | **TU** | **n [p-value]** |
| **Graudens**  **Colon** | 60 | **Normal**  **Colon** | 12 | **Colorectal**  **Carcinoma** | 48 [p>1.0e-4] |
| **Sabates-Bellver**  **Colon** | 64 | **Normal**  **Colon** | 32 | **Colon & Rectal**  **Adenoma** | 32 [p>1.0e-4] |
| **Hong**  **Colorectal** | 82 | **Normal**  **Colon** | 12 | **Colorectal**  **Carcinoma** | 70 [*p=3.24e-14] |
| **TCGA**  **Colorectal** | 237 | **Normal**  **Colon & Rectum** | 22 | **Cecum & Colon & Rectosigmoid**  **& Rectal Adenocarcinoma** | 215 [*p=1.32e-32] |

| **Table S9 *POM121A/C* mRNA up-regulation in GI cancers**  Data were retrieved from *OncoDB*®. Legend: n patient case numbers; NT non-tumor  (normal colon tissue); TU tumor. | | | | | | | |
| --- | --- | --- | --- | --- | --- | --- | --- |
|  | | | | | | **Up-regulation in TU [mRNA]** | |
| **Data set** | **Σn** | **NT** | **n** | **TU** | **n** | ***POM121C*** | ***POM121A*** |
| **COAD** | 349 | **Normal**  **Colon** | 41 | **Colorectal**  **Adenocarcinoma** | 308 | *p=1.6*e-14 | *p=9.5*e-21 |
| **READ** | 104 | **Normal**  **Rectum** | 10 | **Rectal**  **Adenocarcinoma** | 94 | *p=1.4*e-4 | *p=1.6*e-6 |
| **STAD** | 450 | **Normal**  **Stomach** | 35 | **Gastric**  **Adenocarcinoma** | 415 | *p=6.1*e-17 | *p=9.9*e-21 |

| **Table S10 Pathway gene alterations in human cancers ***  * based on cBioPortal® database; all tumors were included in the analysis.  Legend: a = Amplification, b = Deep deletion, c = missense/truncation mutation,  d = mRNA up-regulation, e = mRNA down-regulation, N= number of patient cases. | | | | | | | |
| --- | --- | --- | --- | --- | --- | --- | --- |
| **Data set** | | **Cases altered** | **Type and number of alterations (N)** | | | | |
|  | |  | **a** | **b** | **c** | **d** | **e** |
| **Colorectal Adenocarcinoma (TCGA, Nature 2012)** | | | | | | | |
| *POM121C* | | 29 of 276 (11%) | **3** | **0** | **1** | **20** | **5** |
| *POM121A* | | 27 of 276 (9%) | **2** | **0** | **5** | **19** | **1** |
| **Colorectal Adenocarcinoma (TCGA, Firehose Legacy)** | | | | | | | |
| *POM121C* | | 3 of 640 (0.5%) | **2** | **0** | **1** | **0** | **0** |
| *POM121A* | | 27 of 640 (4%) | **3** | **0** | **5** | **17** | **2** |
| **Colorectal Adenocarcinoma (TCGA, PanCancer Atlas)** | | | | | | | |
| *POM121C* | | 84 of 594 (14%) | **2** | **0** | **12** | **67** | **3** |
| *POM121A* | | 104 of 594 (17%) | **3** | **0** | **12** | **86** | **3** |
| **Stomach Adenocarcinoma (TCGA, PanCancer Atlas)** | | | | | | | |
| *POM121C* | 76 of 440 (15 %) | | **8** | 0 | **10** | **54** | 4 |
| *POM121A* | 90 of 440 (18 %) | | **8** | 1 | **12** | **68** | 1 |
| **Stomach Adenocarcinoma (TCGA, Nature 2014)** | | | | | | | |
| *POM121C* | 37 of 295 (11 %) | | **7** | 0 | **6** | **23** | 1 |
| *POM121A* | 45 of 295 (14 %) | | **6** | 0 | **8** | **30** | 1 |
| **Esophageal Adenocarcinoma (TCGA, PanCancer Atlas)** | | | | | | | |
| *POM121C* | 40 of 182 (20 %) | | **4** | 0 | **0** | **36** | 0 |
| *POM121A* | 27 of 182 (14 %) | | **4** | 1 | **2** | **20** | 0 |
| **Esophageal Carcinoma (TCGA, Nature 2017)** | | | | | | | |
| *POM121C* | 21 of 559 (4 %) | | **13** | 0 | **8** | **0** | 0 |
| *POM121A* | 37 of 559 (7%) | | **15** | 2 | **20** | **0** | 0 |

| **Table S11**  **Correlation of *POM121C* pathway gene alterations with prognosis in human cancers**  Kaplan-Meier analysis based on the cBioPortal® database: * cases with alterations (“ALT“); # cases without alterations (“WT”); § not assessable (“NA”); OS overall survival; DFS disease-free survival. *POM121A* gene alterations were n.s.  $ PanCancer Studies (n=10 studies with total n=76639 cases): Cancer Therapy and Clonal Hematopoiesis (MSK, Nat Genet 2020) 24146 samples; China Pan-cancer (OrigiMed, Nature 2022) 10194 samples; MSK MetTropism (MSK, Cell 2021) 25775 samples; MSK-IMPACT Clinical Sequencing Cohort (MSK, Nat Med 2017) 10945 samples; MSS Mixed Solid Tumors (Broad/Dana-Farber, Nat Genet 2018) 249 samples; Metastatic Solid Cancers (UMich, Nature 2017) 500 samples; Pan-cancer analysis of whole genomes (ICGC/TCGA, Nature 2020) 2922 samples; SUMMIT - Neratinib Basket Study (Multi-Institute, Nature 2018) 141 samples; TMB and Immunotherapy (MSK, Nat Genet 2019) 1661 samples; Tumors with TRK fusions (MSK, Clin Cancer Res 2020) 106 samples. | | | | | |
| --- | --- | --- | --- | --- | --- |
| **Data set** | **Status** | **Total cases** | **Cases deceased** | **Median month**  **survival** | **log-rank test**  **p-value** |
| *[Chr7: POM121C]* | | | | | |
| **Esophageal Adenocarcinoma (TCGA, PanCancer Atlas)** | | | | | |
| **OS** | ALT | 36 | 11 | 47.93 | 0.130 |
|  | WT | 146 | 65 | 25.08 |  |
| **Esophageal Adenocarcinoma (TCGA, Provisional)** | | | | | |
| **OS** | ALT | 37 | 11 | 47.9 | **0.0883** |
|  | WT | 148 | 66 | 25.07 |  |
| **Esophageal Carcinoma (TCGA, Nature 2017)** | | | | | |
| **OS** | ALT | 21 | 7 | 37.93 | 0.566 |
|  | WT | 519 | 192 | 28.98 |  |
| **Colorectal Adenocarcinoma (TCGA, PanCancer Atlas)** | | | | | |
| **OS** | ALT | 82 | 16 | 49.41 | 0.640 |
|  | WT | 508 | 104 | 92.74 |  |
| **Colorectal Adenocarcinoma (TCGA, Nature 2012)** | | | | | |
| **OS** | ALT | 28 | 3 | NA | 0.966 |
|  | WT | 244 | 22 | NA |  |
| **Colorectal Adenocarcinoma (TCGA, Firehose Legacy)** | | | | | |
| **OS** | ALT | 3 | 0 | NA | 0.622 |
|  | WT | 619 | 129 | 83.18 |  |
| **PanCancer Studies $** | | | | | |
| **OS** | ALT | 25 | 14 | 19.74 | **0.110** |
|  | WT | 506 | 213 | 37.73 |  |

| **Table S12 *POM121A/C* gene mutations in GI cancers**  Types of mutations annotated in the cBioPortal® database: * nonsense (stop codon); X splice;  - fusion; **bold** putative N-terminal truncation (missense/other mutations are not listed).  Abbrev: CCLE Cancer Cell Line Encyclopedia; CRC colorectal cancer; EC esophageal cancer;  GC gastric cancer; NCI National Cancer Institute.  § Cancer Cell Line Encyclopedia (Broad, 2019) 1739 samples; Cancer Cell Line Encyclopedia (Novartis/Broad, Nature 2012) 1020 samples; NCI-60 Cell Lines (NCI, Cancer Res 2012) 67 samples  $ PanCancer Studies (n=10 studies) as listed in **Table S11**; **Σ** Combined Studies for Rectal, Colon, Colorectal, Esophageal and Stomach/Gastric Cancer/Carcinoma/Adenocarcinoma accessed 2023 in cBioportal®. | | | | |
| --- | --- | --- | --- | --- |
| **Data set** | **Studies**  **(n)** | **Sample number (N)** | ***POM121A*** | ***POM121C*** |
| *[Chr7: POM121A/C]* | | | | |
| **Cell lines [combined studies]** | | | | |
| **CCLE and NCI-60 §** | | | | |
|  | 3 | 2826 | **R15*,R250***  G860*,S930* | **X318_splice**, X1197_splice, Q1220*,POM121C-TBCD |
| **Cancer tissues [combined studies]** | | | | |
| **“PanCancer Studies” $** | | | | |
|  | 10 | 76639 | **X456_splice** | None |
| **“Bowel” Σ** | | | | |
|  | 13 | 4535 | **X521_splice,**  **X497_splice** | **Q522*** |
| **“Esophagus/Stomach” Σ** | | | | |
|  | 17 | 3791 | **R156*,**  **X380_splice** | None |
| **Colorectal cancer tissues [single studies]** | | | | |
| **Colorectal Adenocarcinoma (TCGA, PanCancer Atlas)** | | | | |
|  | 1 | 594 | **X497_splice** | None |
| **Colorectal Adenocarcinoma (TCGA, Firehose Legacy)** | | | | |
|  | 1 | 640 | **X521_splice** | None |
| **Colorectal Adenocarcinoma (TCGA, Nature 2012)** | | | | |
|  | 1 | 276 | **X521_splice** | None |

| **Table S13 POM121 protein structures predicted by I-TASSER**  Top 5 proteins from the PDB which have the closest structural similarity (i.e. the highest TM-score) to the predicted I-TASSER model. Legend: C-score is the confidence score for estimating the quality of the predicted models by I-TASSER. It is calculated based on the significance of threading template alignments and the convergence parameters of the structure assembly simulations. The C-score is typically in the range of [-5,+2], where a C-score of higher value signifies a model with a high confidence and vice versa. The TM-score is a scale for measuring the structural similarity between two structures; a TM-score >0.5 indicates a model of correct topology and a TM-score<0.17 means random similarity. RMSD (root-mean-square deviation) is non-negative, and a value of 0 indicates a perfect fit to the data. In general, a lower RMSD is better than a higher one. IDEN is the percentage of sequence identity in the structurally aligned region. Cov represents the coverage of the threading alignment and is equal to the number of aligned residues divided by the length of query protein. | | | | | | | | |
| --- | --- | --- | --- | --- | --- | --- | --- | --- |
| **Rank** | **C score** | **PDB** | **TM**  **score** | **RMSD** | **IDEN** | **Cov** | **Protein**  **name** | **UniProt**  **ID** |
| A | **-1.19** | 3gavA | **0.937** | **1.05** | 0.063 | 0.944 | Solution structure of Human Complement  Factor H in 137 mM NaCl buffer | P08603  CFAH_HUMAN |
| B | -1.60 | 6reyc | 0.257 | 9.69 | 0.061 | 0.400 | Human 20S-PA200  Proteasome Complex | P60900  PSA6_HUMAN |
| C | -3.43 | 5vkqA | 0.212 | 9.47 | 0.030 | 0.324 | Structure of a mechanotransduction ion  channel Drosophila NOMPC in nanodisc | E0A9E1  E0A9E1_DROME |
| D | -3.96 | 2uv8G | 0.212 | 9.09 | 0.029 | 0.316 | Crystal structure of yeast fatty acid synthase with stalled acyl carrier protein at 3.1 Å resolution | P19097  FAS2_YEAST |
| E | -4.22 | 5jcss | 0.211 | 9.70 | 0.030 | 0.328 | Cryo-EM structure of the RIX1-REA1  Pre-60S particle | P0CX45  RL2A_YEAST |
