# Supplementary material for: Nuclear pore protein POM121 regulates subcellular localization and transcriptional activity of PPARγ
Source: Cell Death Dis. 2024 Jan 4;15(1):7. doi: 10.1038/s41419-023-06371-1 (PMC10766976; doi:10.1038/s41419-023-06371-1)
Supplement: Supplementary file 4 — Original Western Blots [file 41419_2023_6371_MOESM4_ESM.pdf]

Supplement

„Original Western Blots“

Fig.1A

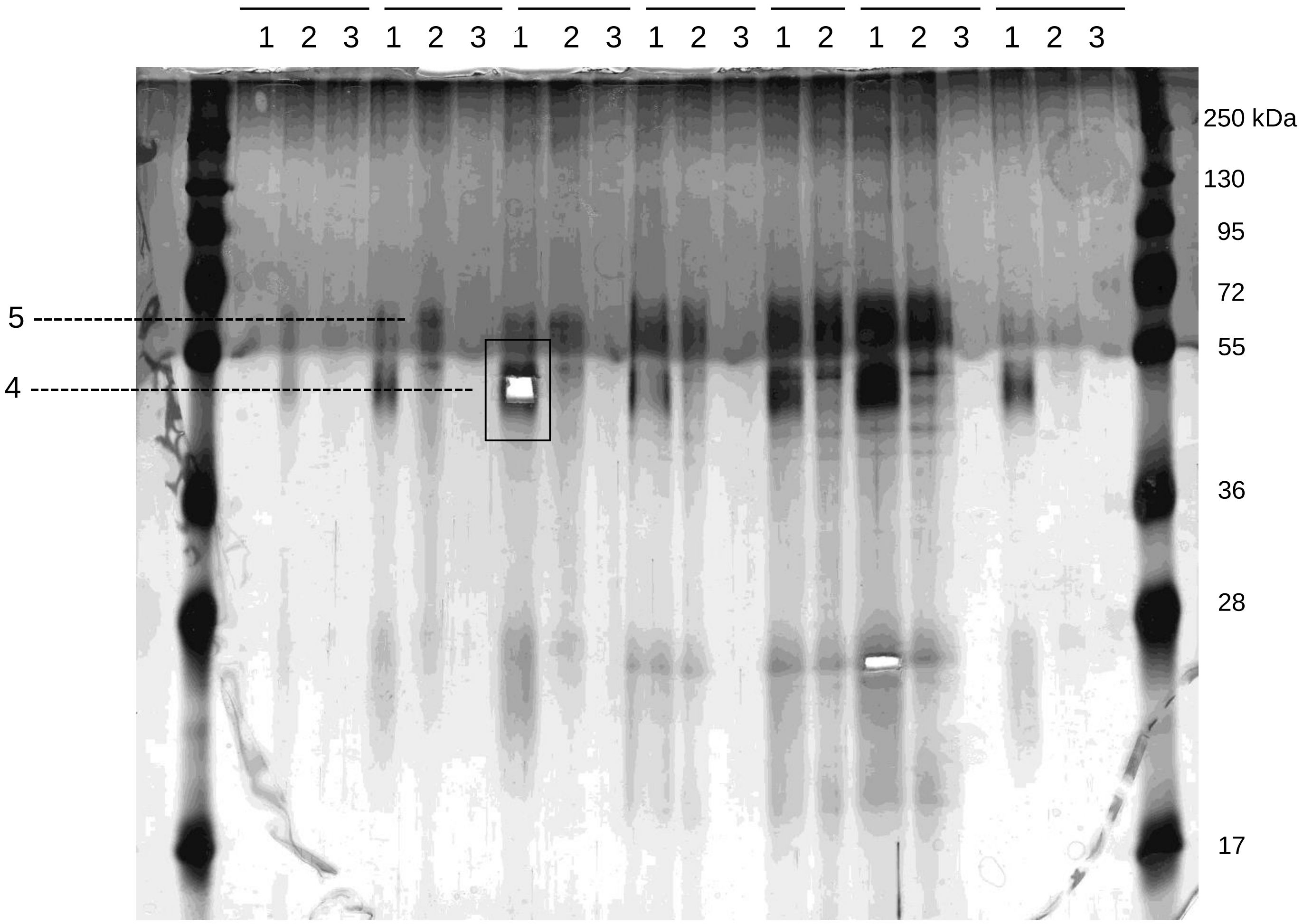

1 = IP with PPARg Ab
2 = IP with empty beads (neg. control)
3 = empty lane
4 = excised band sent to MS (POM121 peptides)
5 = IgH

Fig.1D

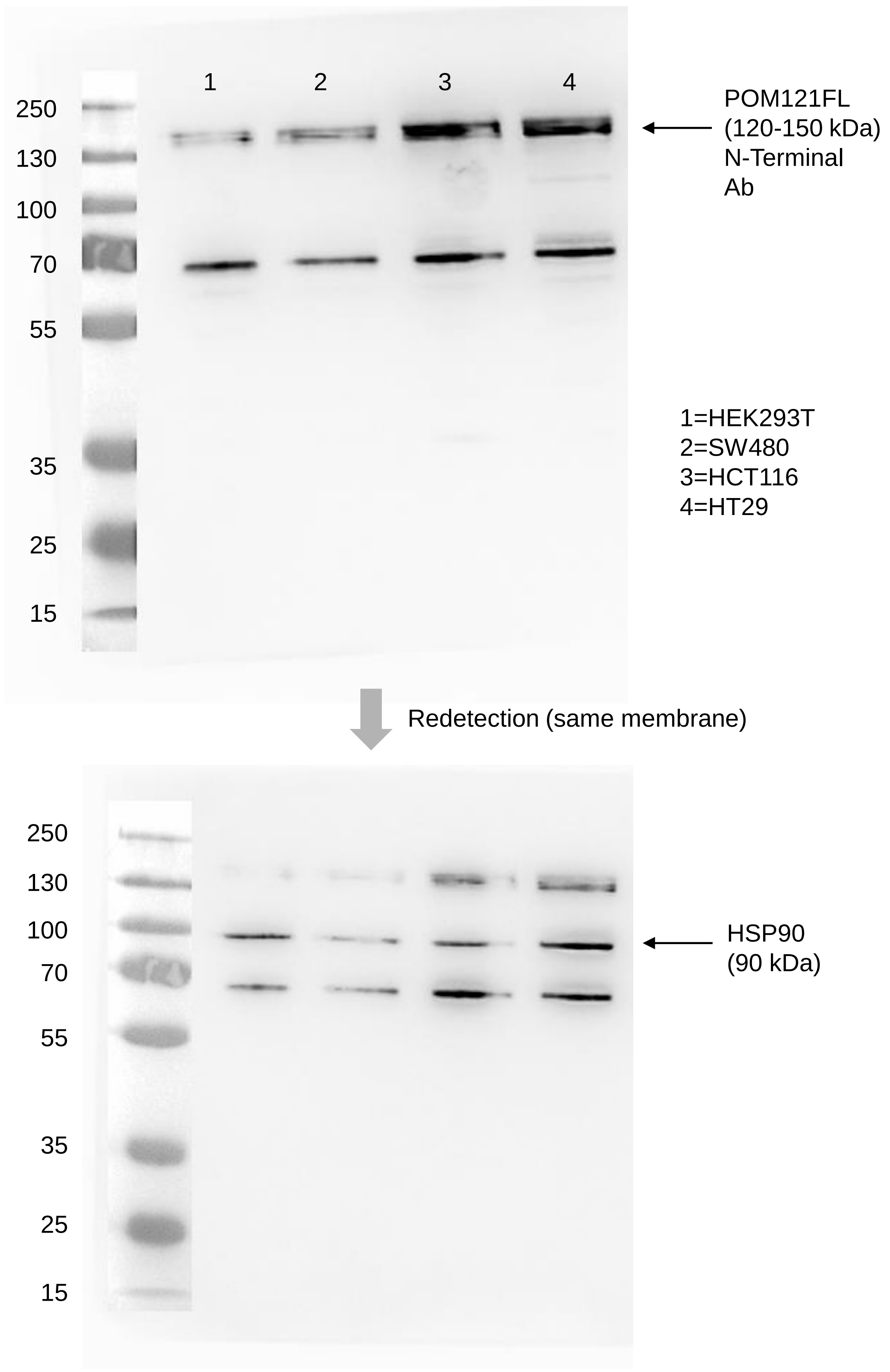
1
2
3
4
250
130
100
70
55
35
25
15
POM121FL
(120-150 kDa)
N-Terminal
Ab
1=HEK293T
2=SW480
3=HCT116
4=HT29
Redetection (same membrane)
250
130
100
70
55
35
25
15
HSP90
(90 kDa)

Fig.2C

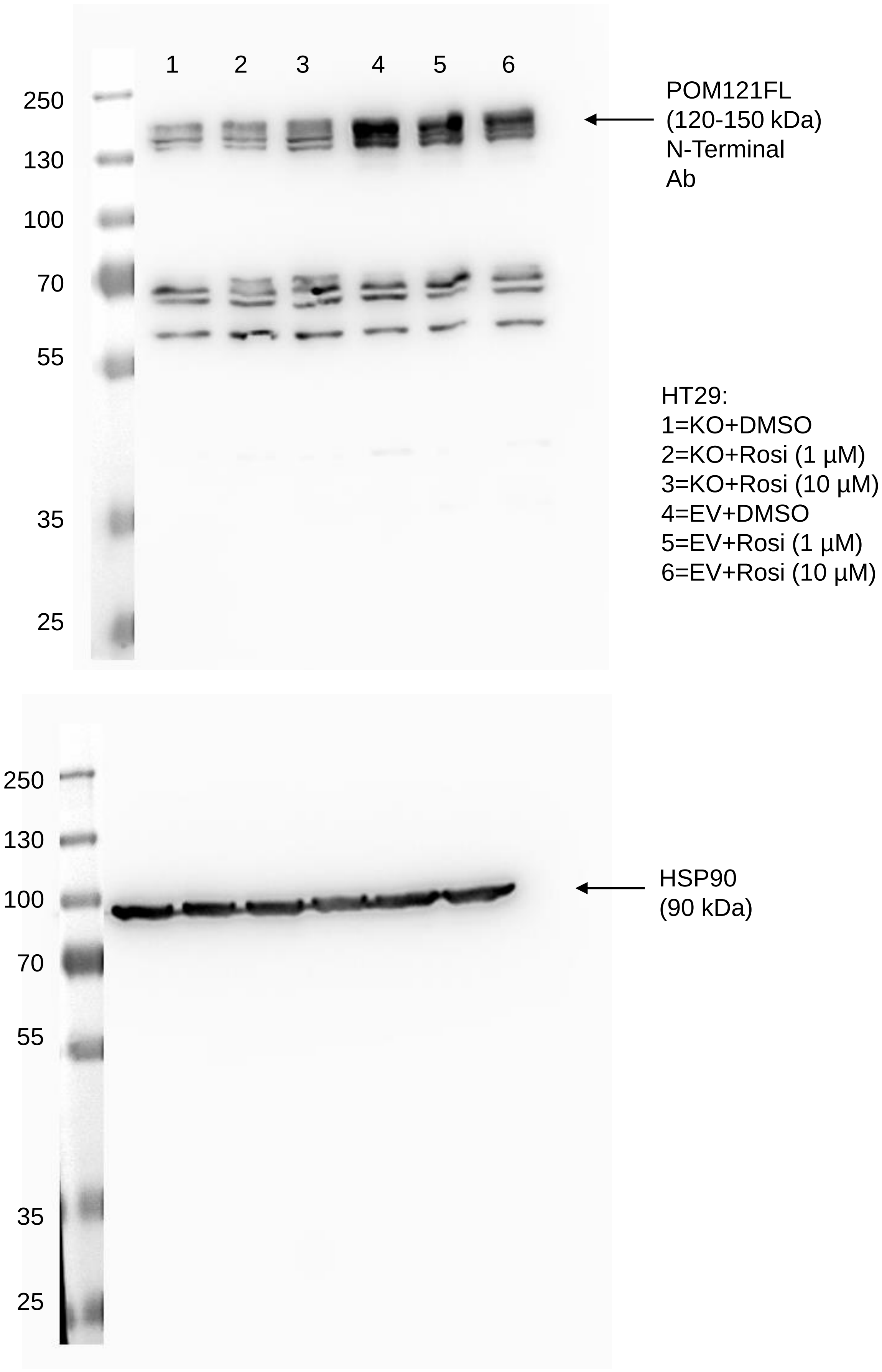
1
2
3
4
5
6
250
130
100
70
55
35
25
POM121FL
(120-150 kDa)
N-Terminal
Ab
HT29:
1=KO+DMSO
2=KO+Rosi (1 µM)
3=KO+Rosi (10 µM)
4=EV+DMSO
5=EV+Rosi (1 µM)
6=EV+Rosi (10 µM)
250
130
100
70
55
35
25
HSP90
(90 kDa)

Fig.3B

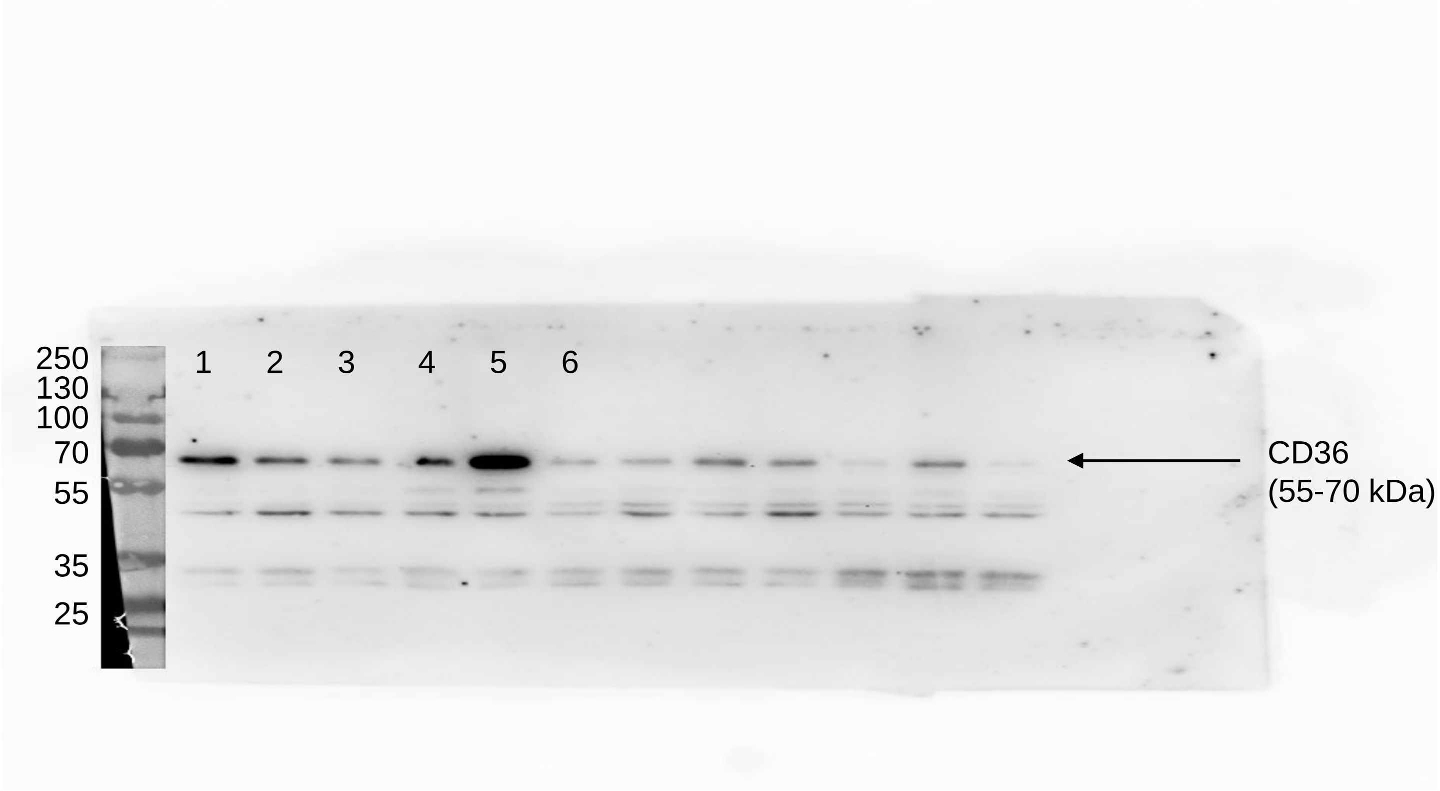

HT29:
1=KO+DMSO
2=KO+Rosi (1 µM)
3=KO+Rosi (10 µM)
4=EV+DMSO
5=EV+Rosi (1 µM)
6=EV+Rosi (10 µM)

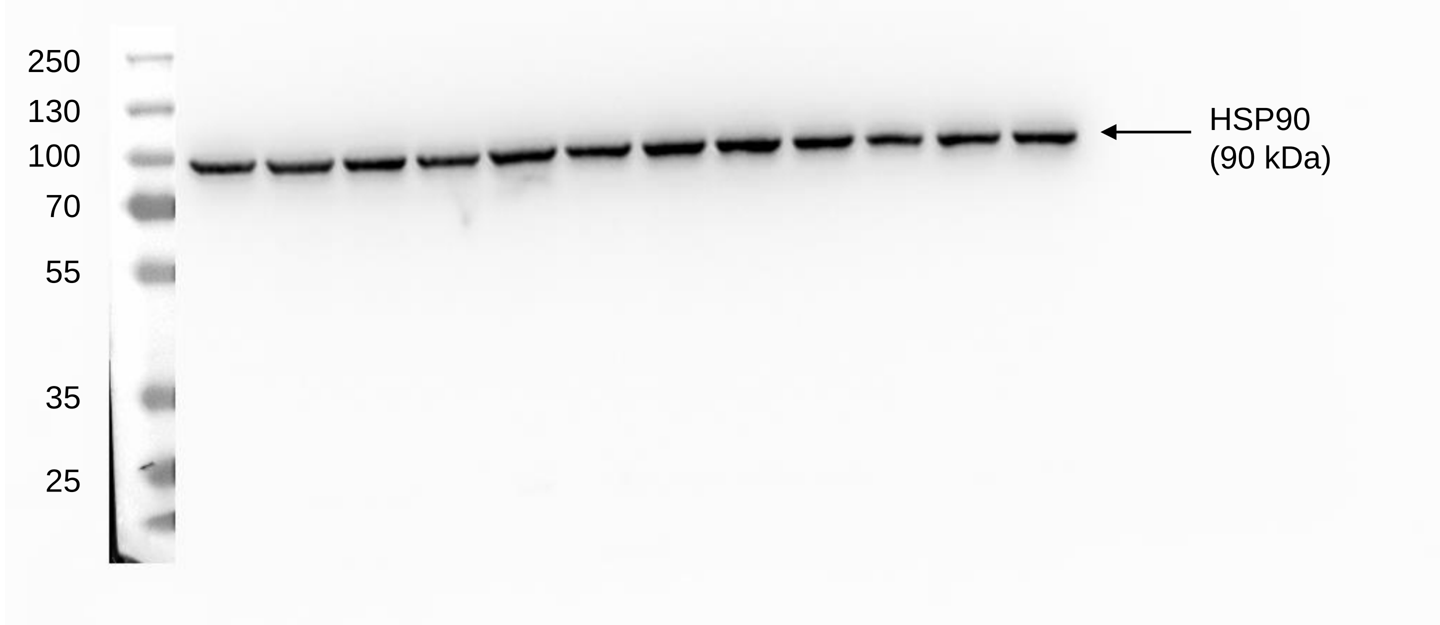

Fig.3B cont.

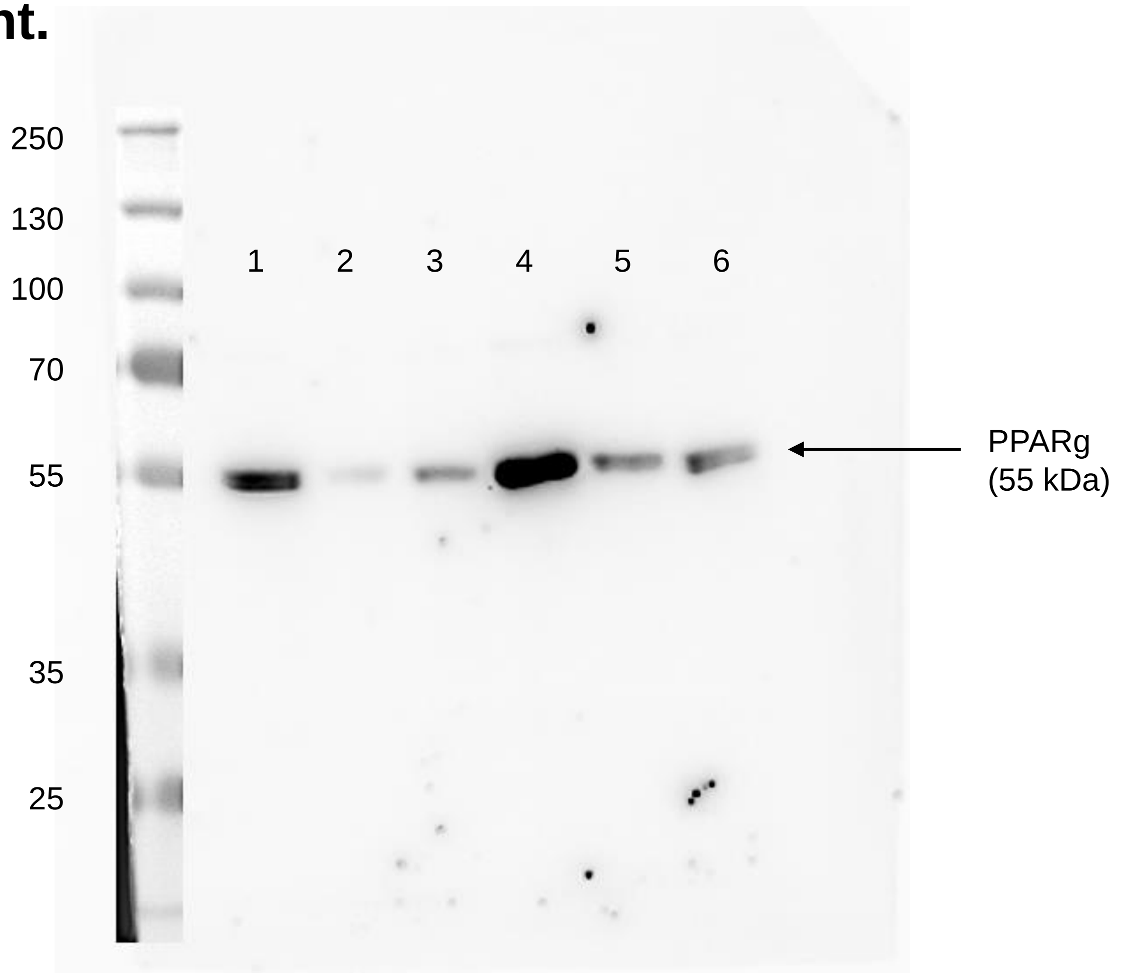

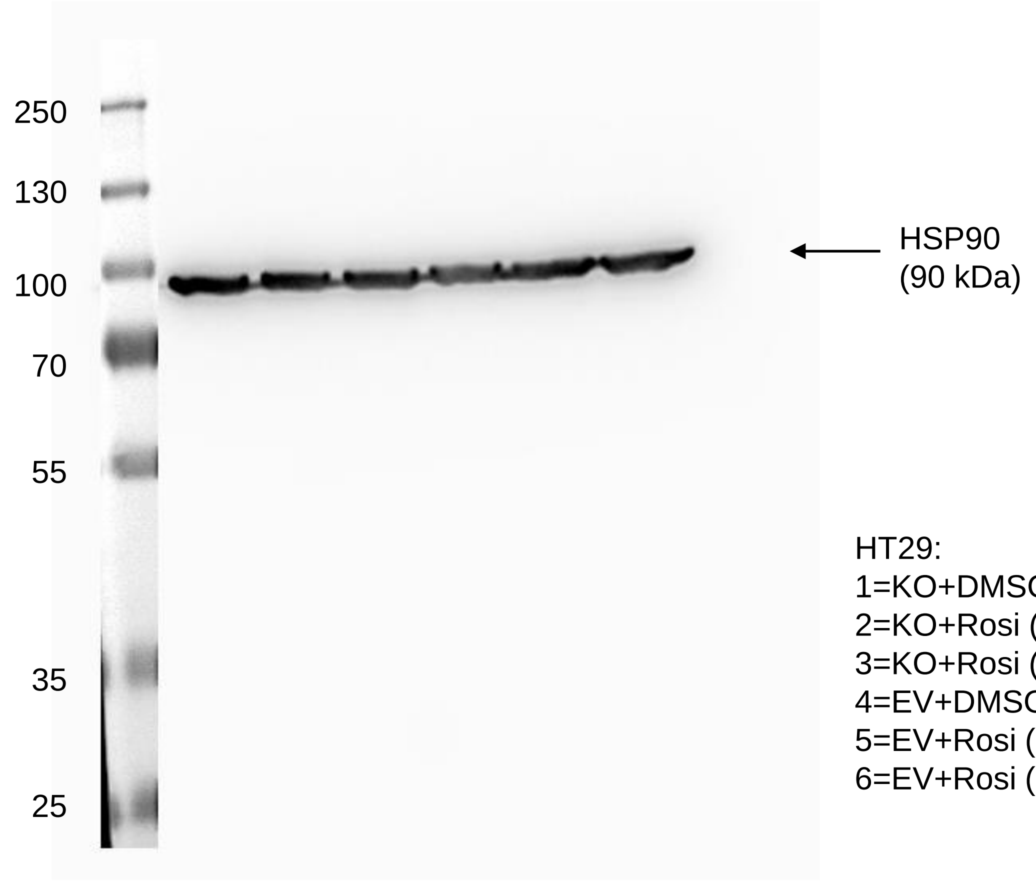

HT29:
1=KO+DMSO
2=KO+Rosi (1 µM)
3=KO+Rosi (10 µM)
4=EV+DMSO
5=EV+Rosi (1 µM)
6=EV+Rosi (10 µM)

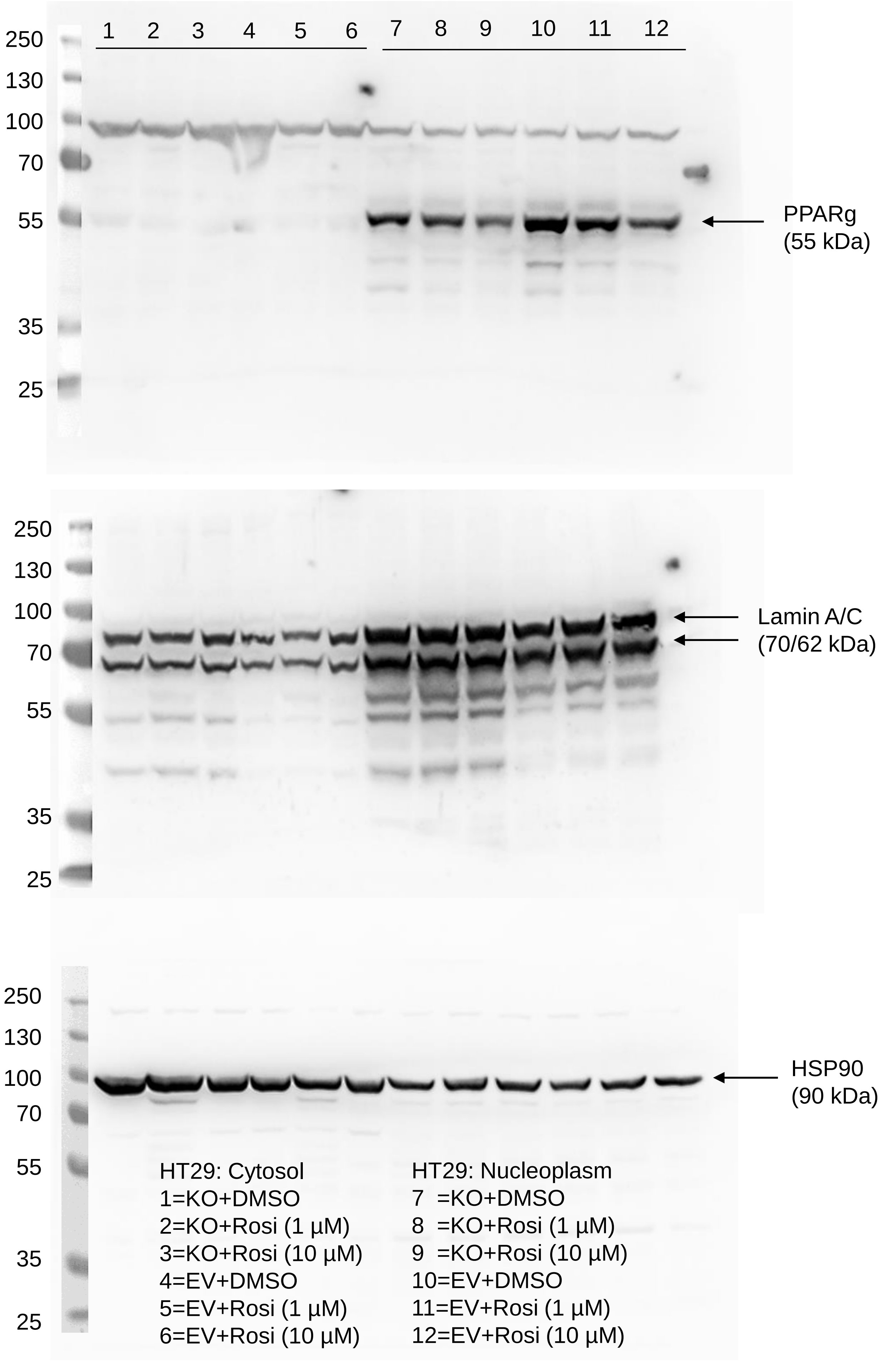
1 2 3 4 5 6 7 8 9 10 11 12
250
130
100
70
55
35
25
PPARg
(55 kDa)
Lamin A/C
(70/62 kDa)
HSP90
(90 kDa)
HT29: Cytosol
1=KO+DMSO
2=KO+Rosi (1 µM)
3=KO+Rosi (10 µM)
4=EV+DMSO
5=EV+Rosi (1 µM)
6=EV+Rosi (10 µM)
HT29: Nucleoplasm
7 =KO+DMSO
8 =KO+Rosi (1 µM)
9 =KO+Rosi (10 µM)
10=EV+DMSO
11=EV+Rosi (1 µM)
12=EV+Rosi (10 µM)

Fig.3D

Fig.3D cont.

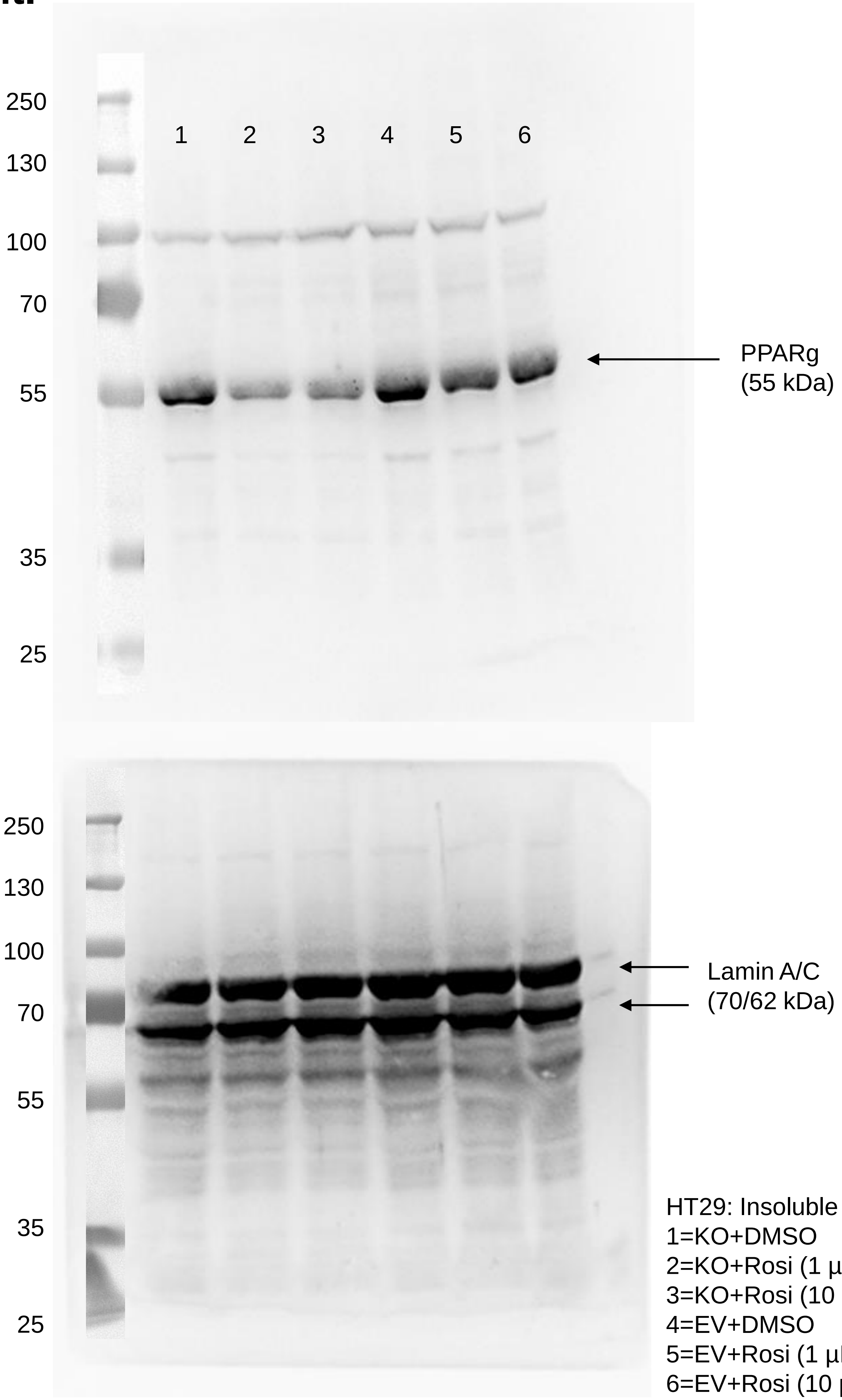

HT29: Insoluble
1=KO+DMSO
2=KO+Rosi (1 µM)
3=KO+Rosi (10 µM)
4=EV+DMSO
5=EV+Rosi (1 µM)
6=EV+Rosi (10 µM)

Fig.3D cont.

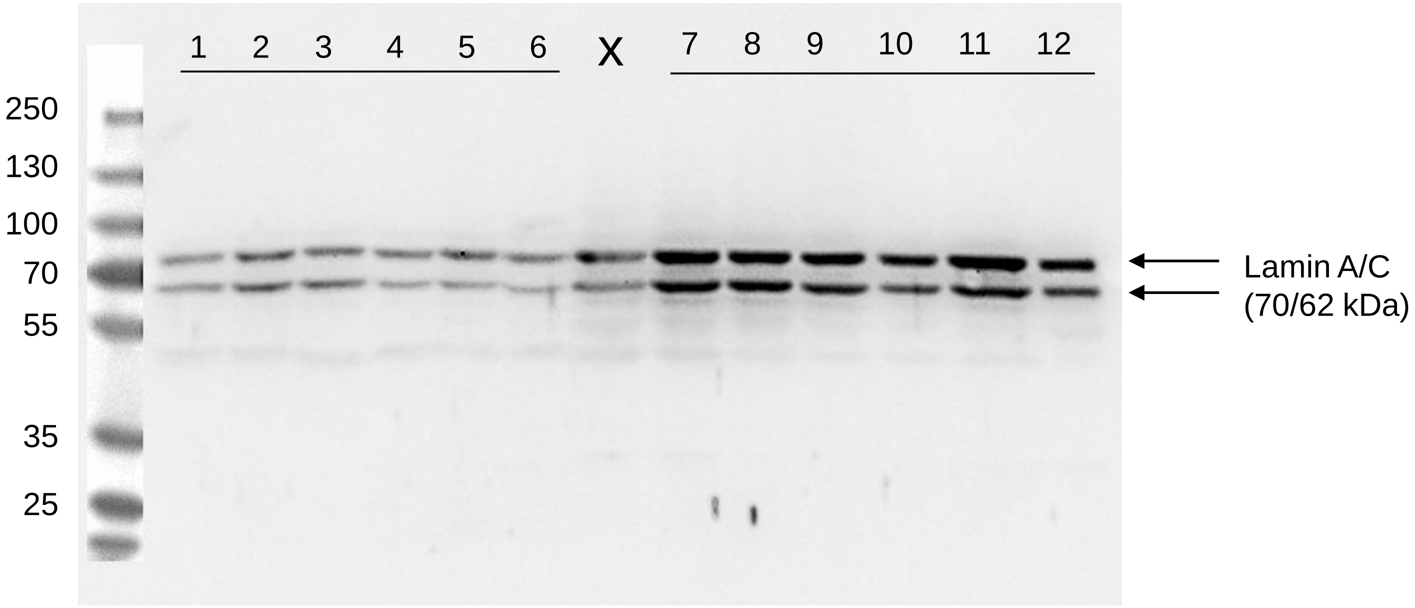

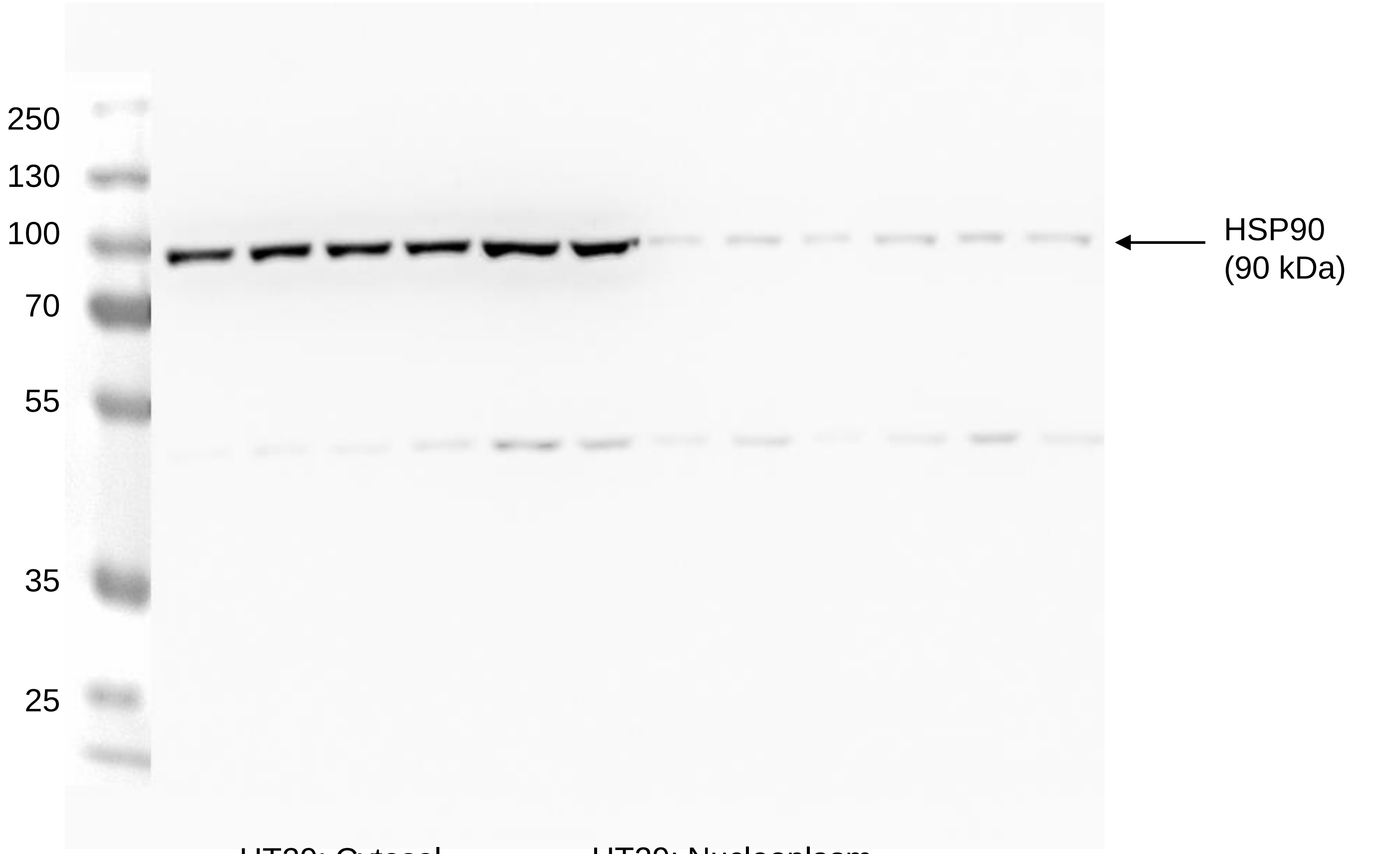

HT29: Cytosol
1=KO+DMSO
2=KO+Rosi (1 µM)
3=KO+Rosi (10 µM)
4=EV+DMSO
5=EV+Rosi (1 µM)
6=EV+Rosi (10 µM)

HT29: Nucleoplasm
7 =KO+DMSO
8 =KO+Rosi (1 µM)
9 =KO+Rosi (10 µM)
10=EV+DMSO
11=EV+Rosi (1 µM)
12=EV+Rosi (10 µM)

Fig.5A HT29

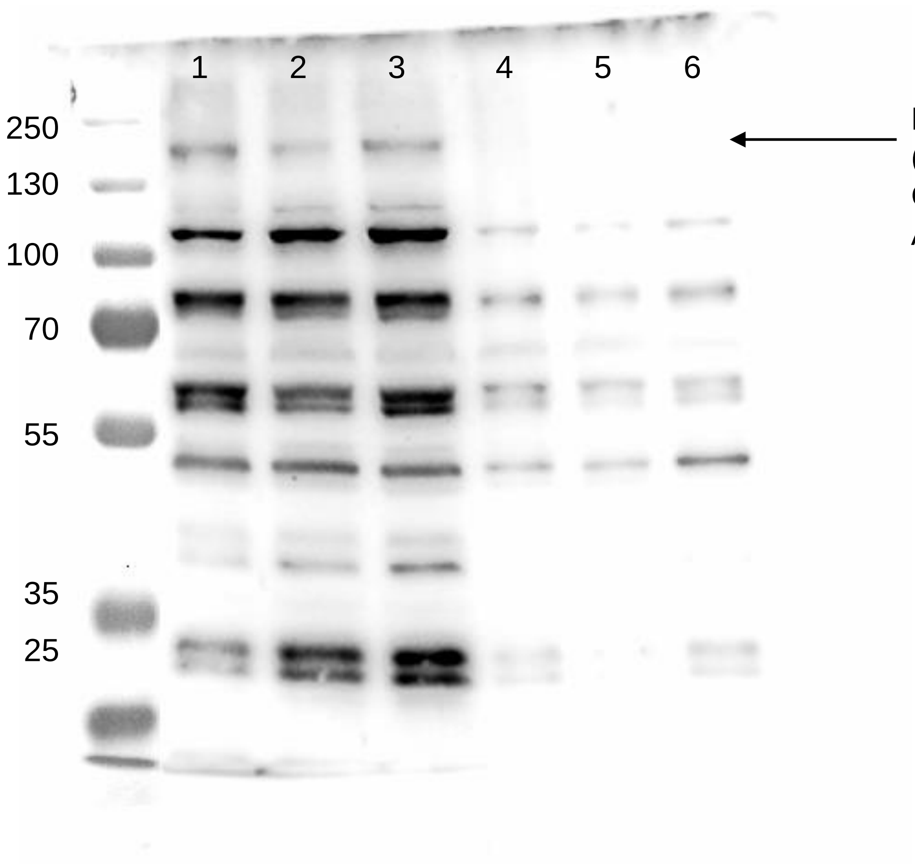

POM121FL
(120-150 kDa)
C-Terminal
Ab

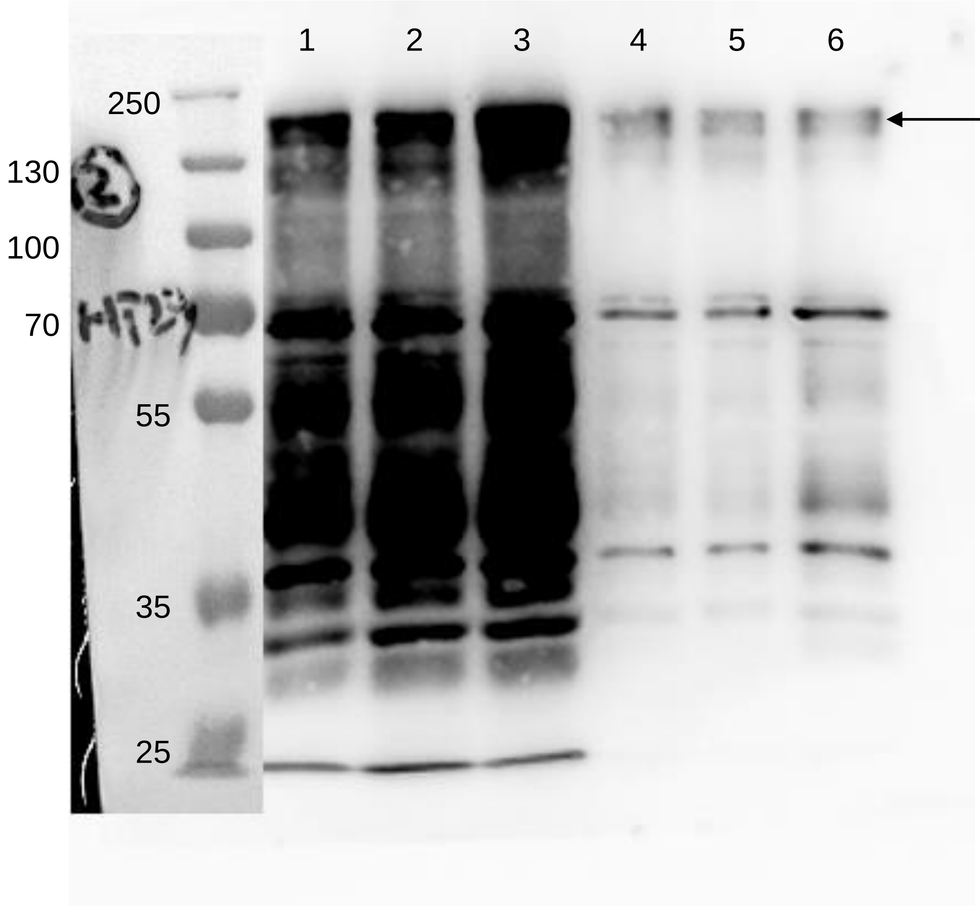

POM121FL
(120-150 kDa)
N-Terminal
Ab

HT29: siRNA
1=Control+DMSO
2=Control+Rosi (1 µM)
3=Control+Rosi (10 µM)
4=POM121+DMSO
5=POM121+Rosi (1 µM)
6=POM121+Rosi (10 µM)

Fig.5A HT29 cont.

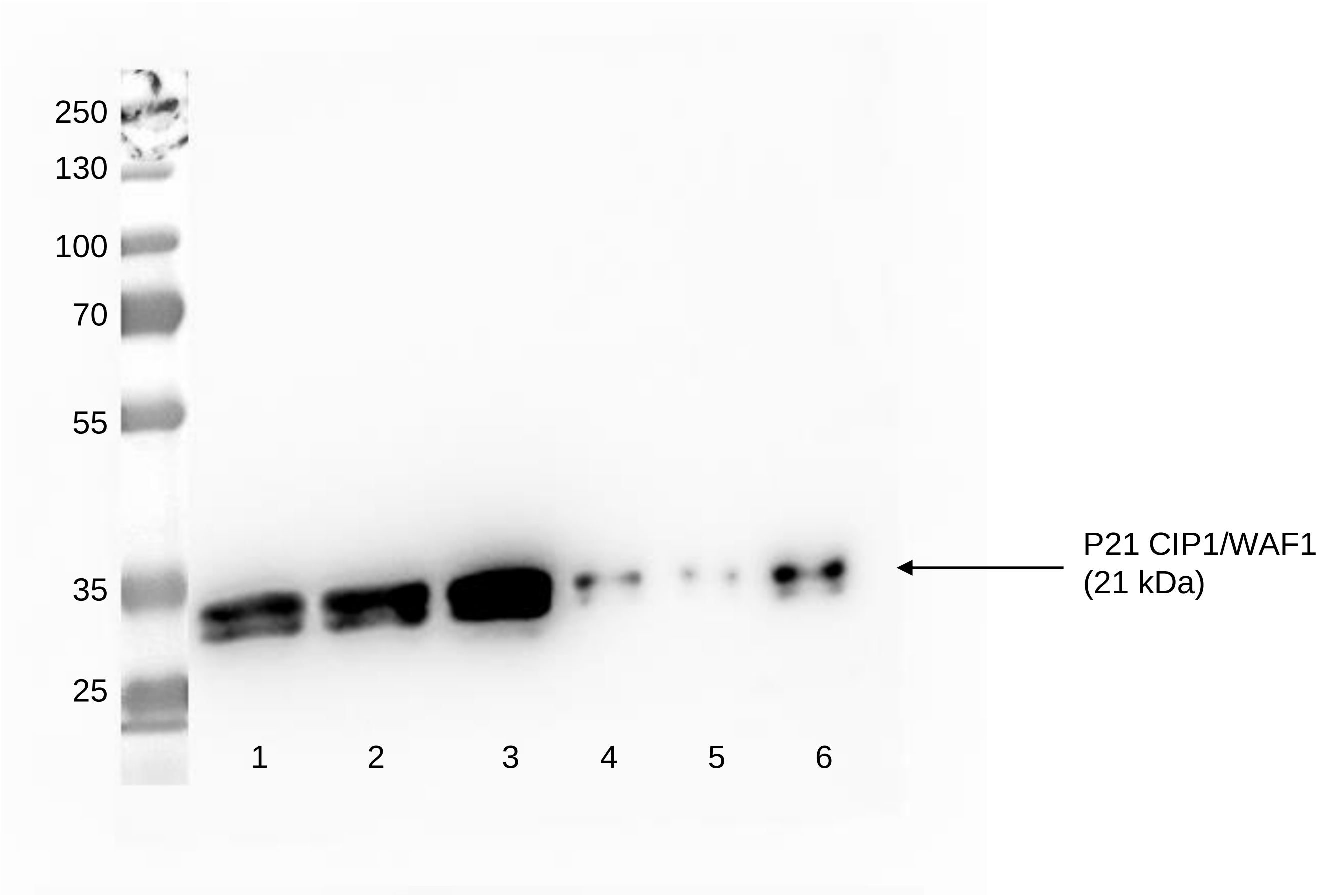

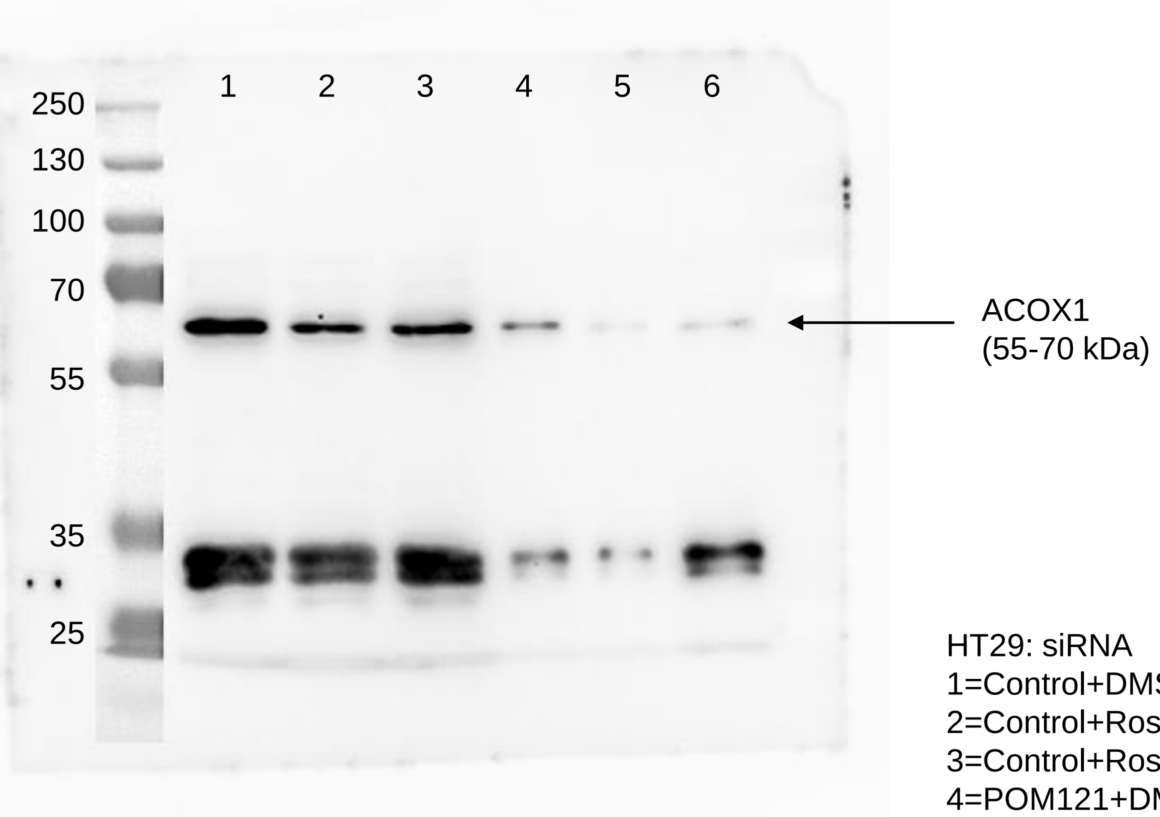

HT29: siRNA
1=Control+DMSO
2=Control+Rosi (1 µM)
3=Control+Rosi (10 µM)
4=POM121+DMSO
5=POM121+Rosi (1 µM)
6=POM121+Rosi (10 µM)

Fig.5A HT29 cont.

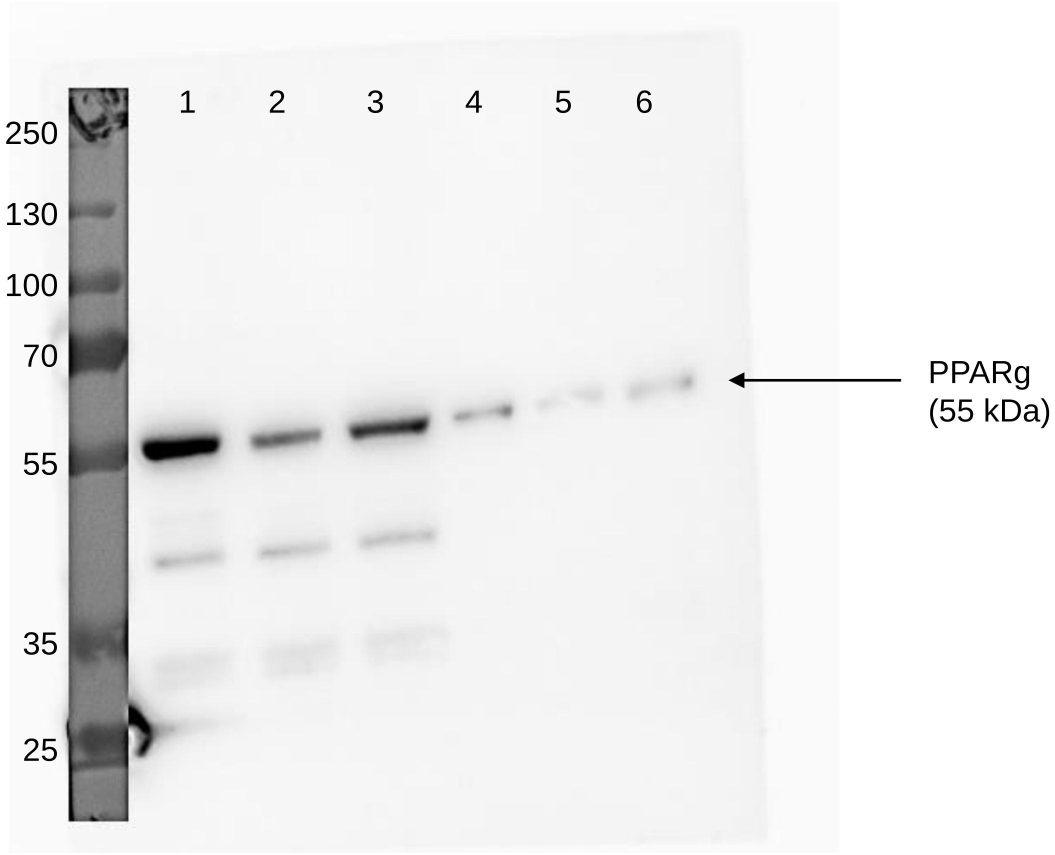

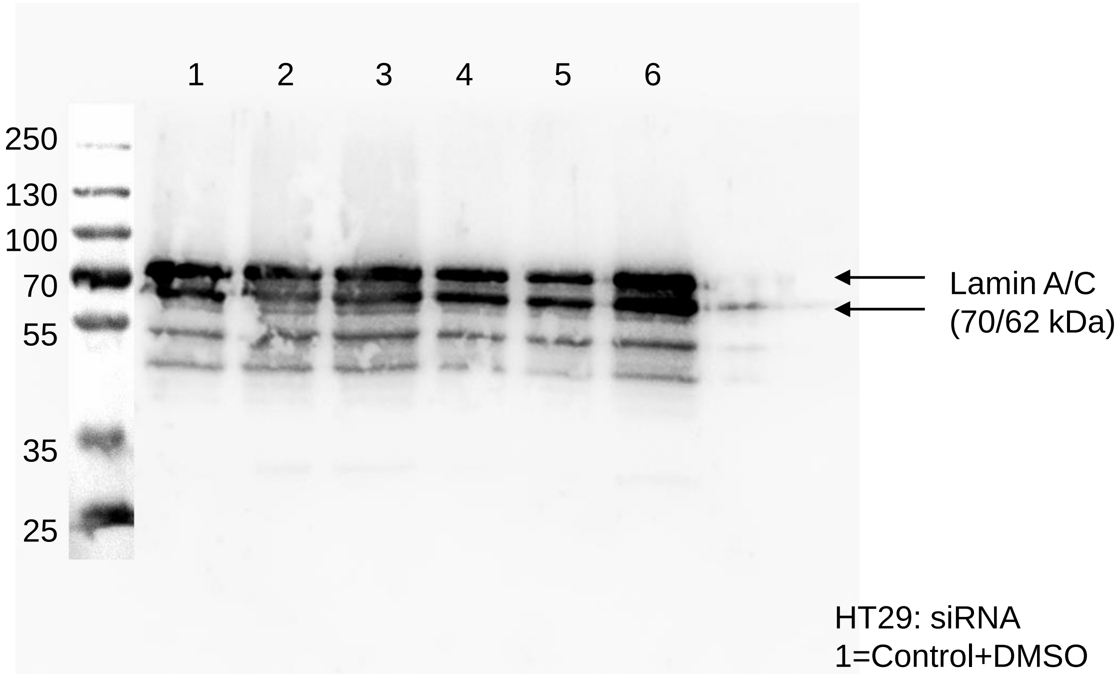

HT29: siRNA
1=Control+DMSO
2=Control+Rosi (1 µM)
3=Control+Rosi (10 µM)
4=POM121+DMSO
5=POM121+Rosi (1 µM)
6=POM121+Rosi (10 µM)

Fig.5A&S16 SW480

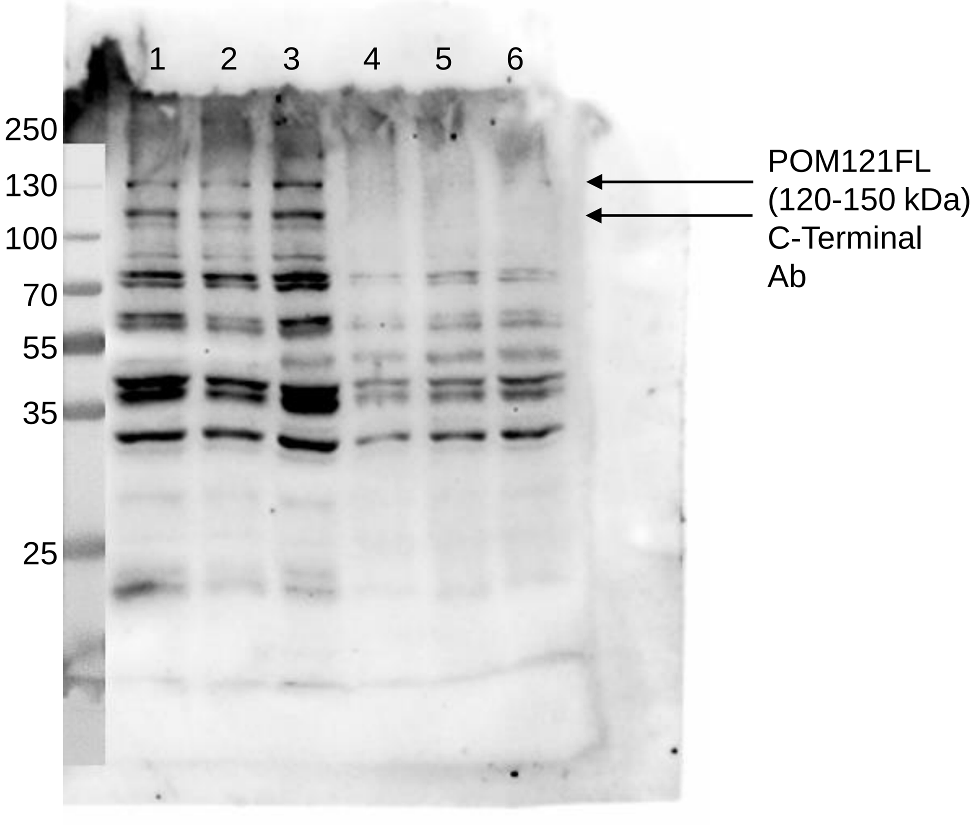

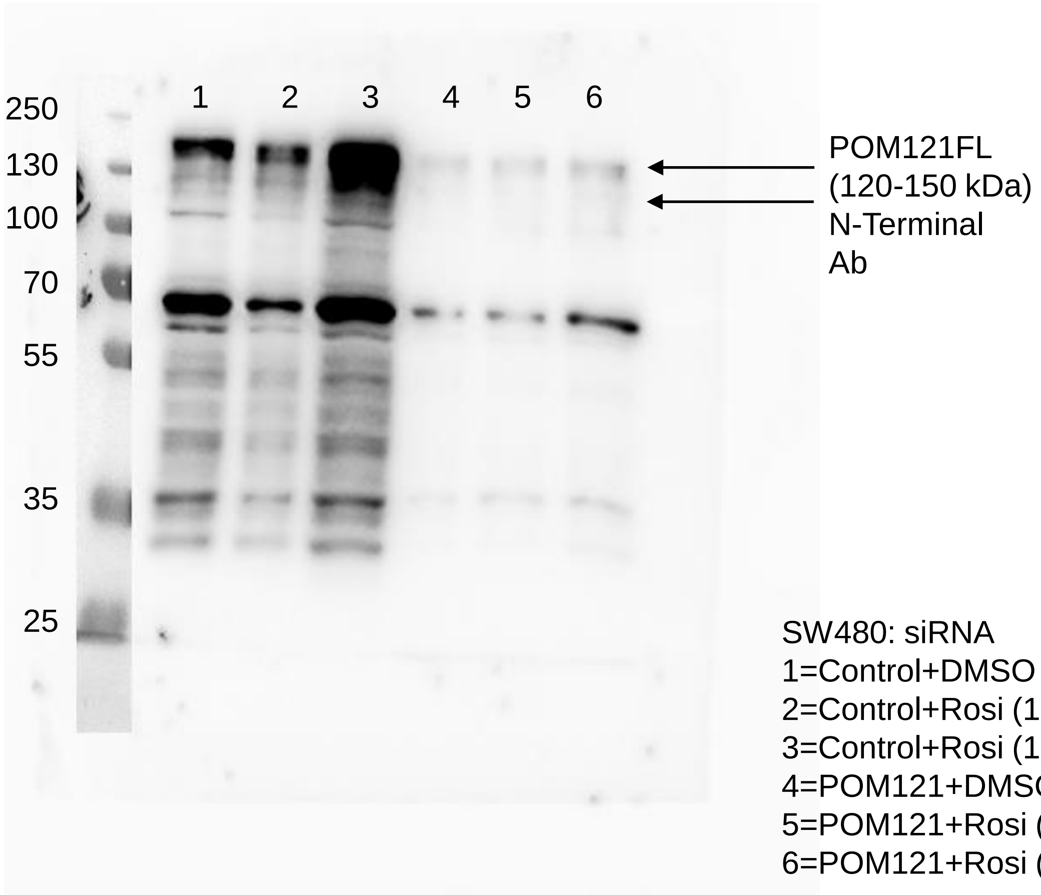

SW480: siRNA
1=Control+DMSO
2=Control+Rosi (1 µM)
3=Control+Rosi (10 µM)
4=POM121+DMSO
5=POM121+Rosi (1 µM)
6=POM121+Rosi (10 µM)

Fig.5A&S16 SW480

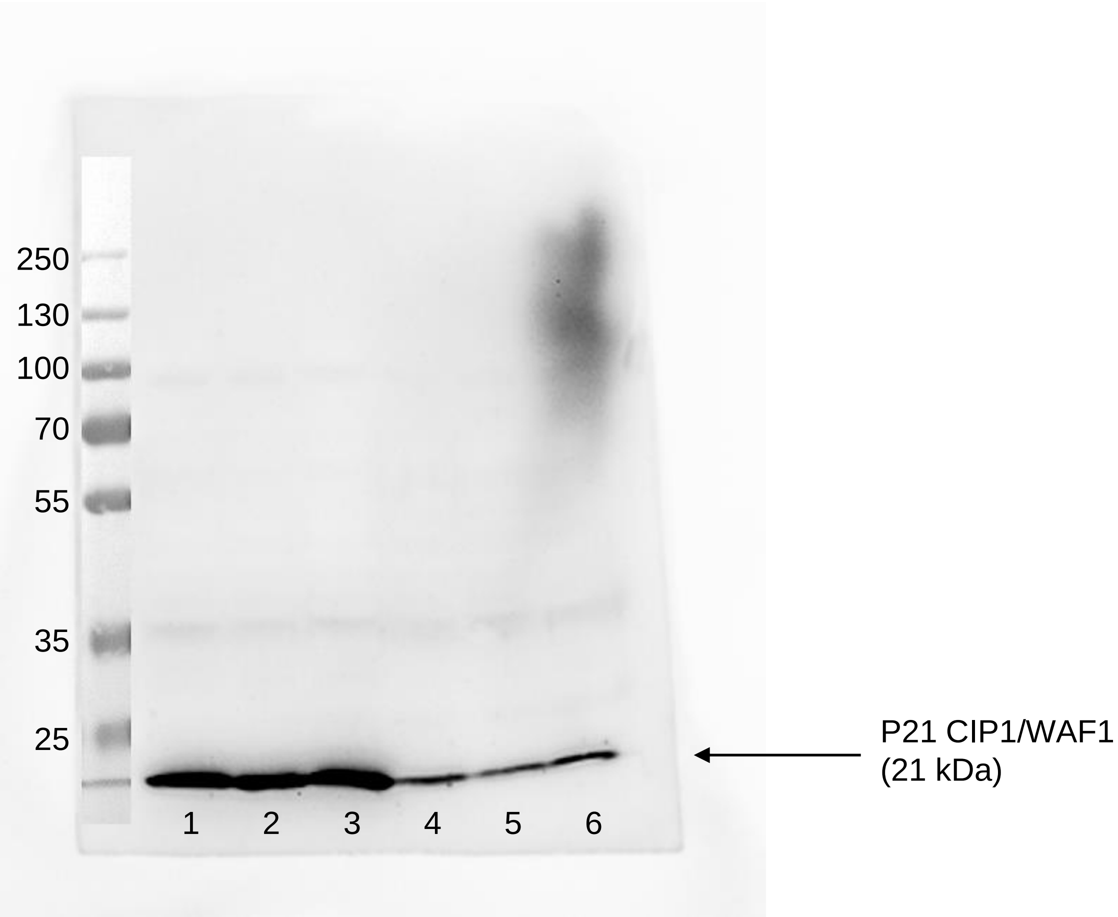

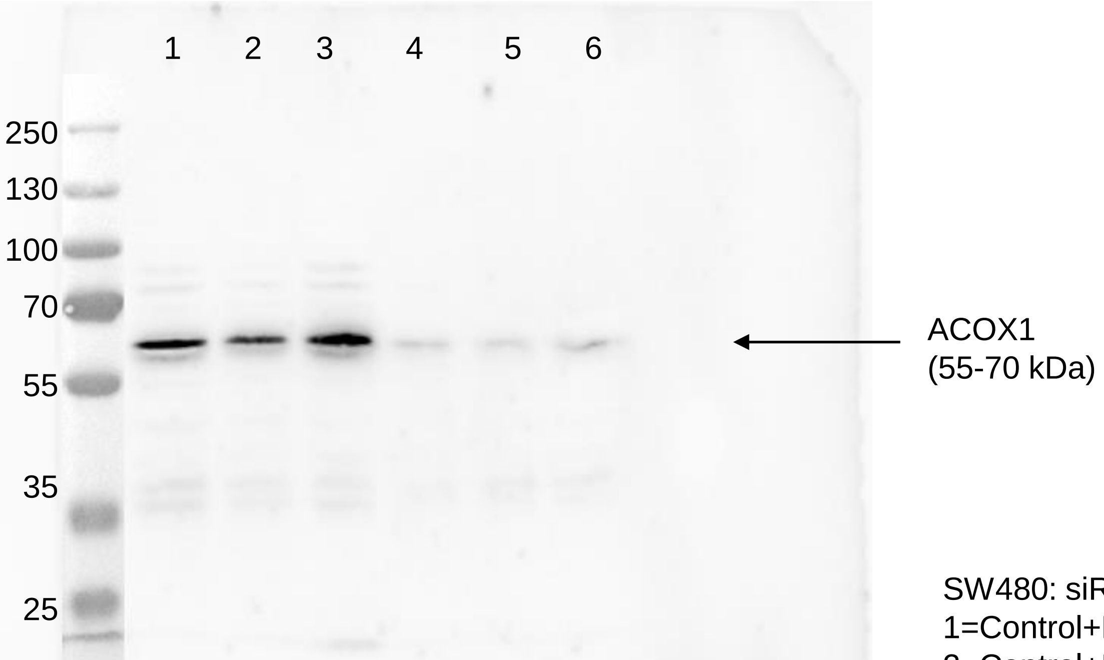

SW480: siRNA
1=Control+DMSO
2=Control+Rosi (1 μM)
3=Control+Rosi (10 μM)
4=POM121+DMSO
5=POM121+Rosi (1 μM)
6=POM121+Rosi (10 μM)

Fig.5A&S16 SW480

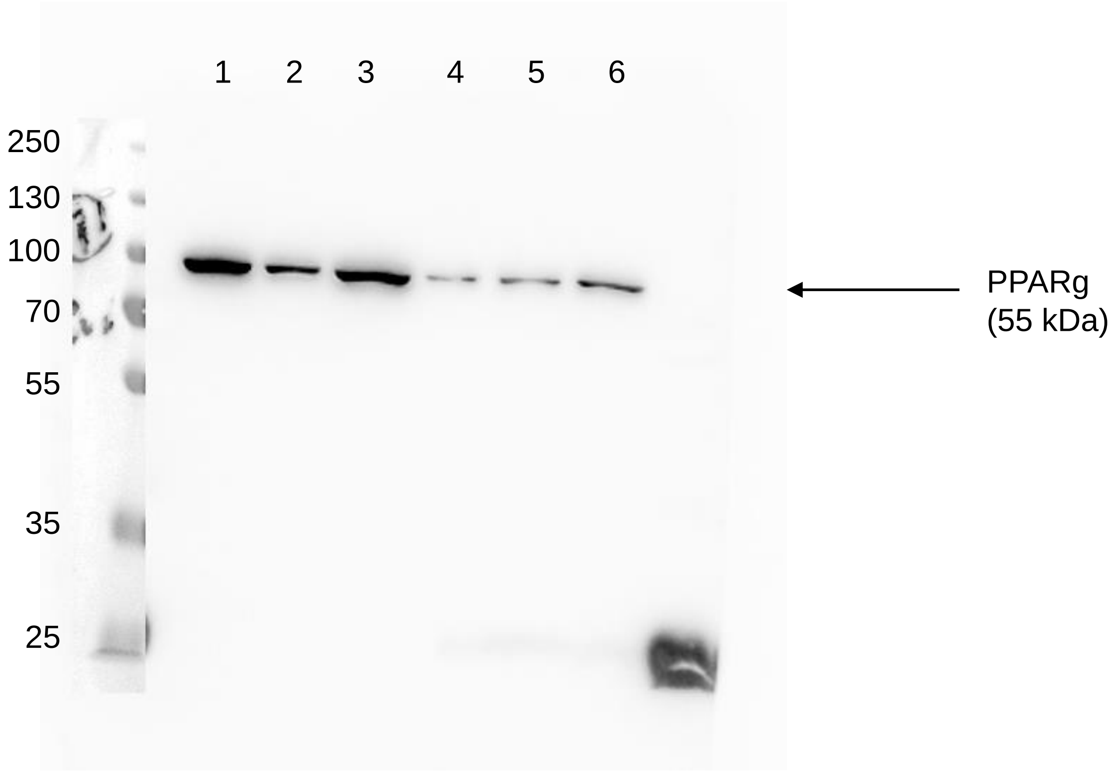

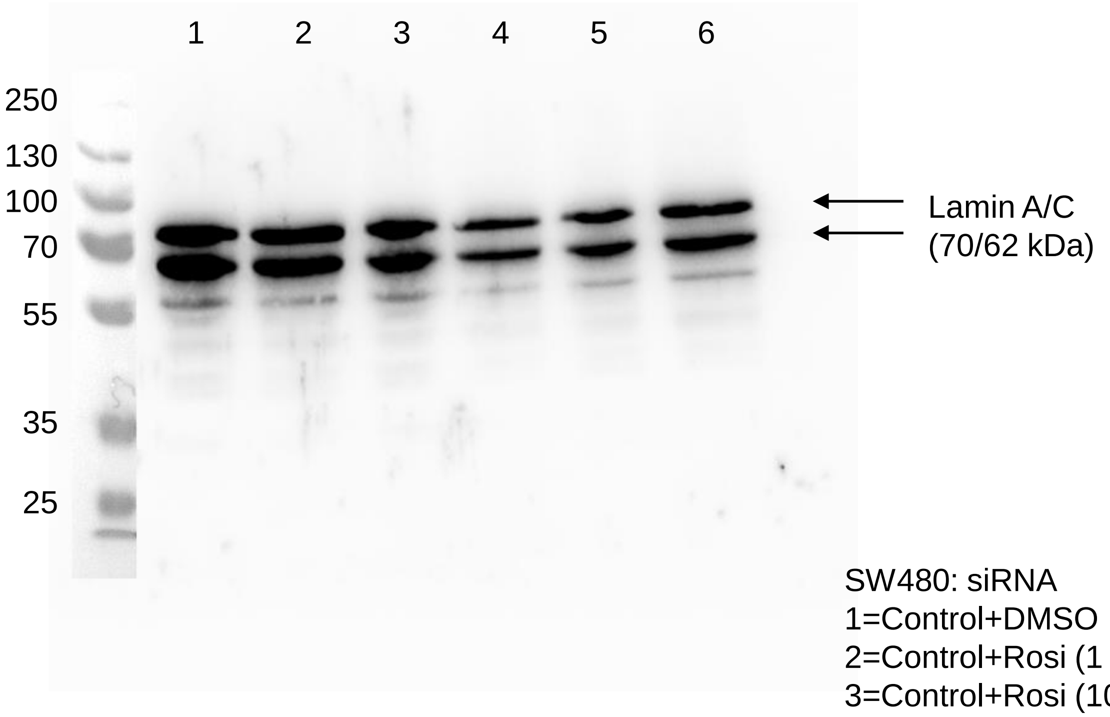

SW480: siRNA
1=Control+DMSO
2=Control+Rosi (1 µM)
3=Control+Rosi (10 µM)
4=POM121+DMSO
5=POM121+Rosi (1 µM)
6=POM121+Rosi (10 µM)

S16 HT29

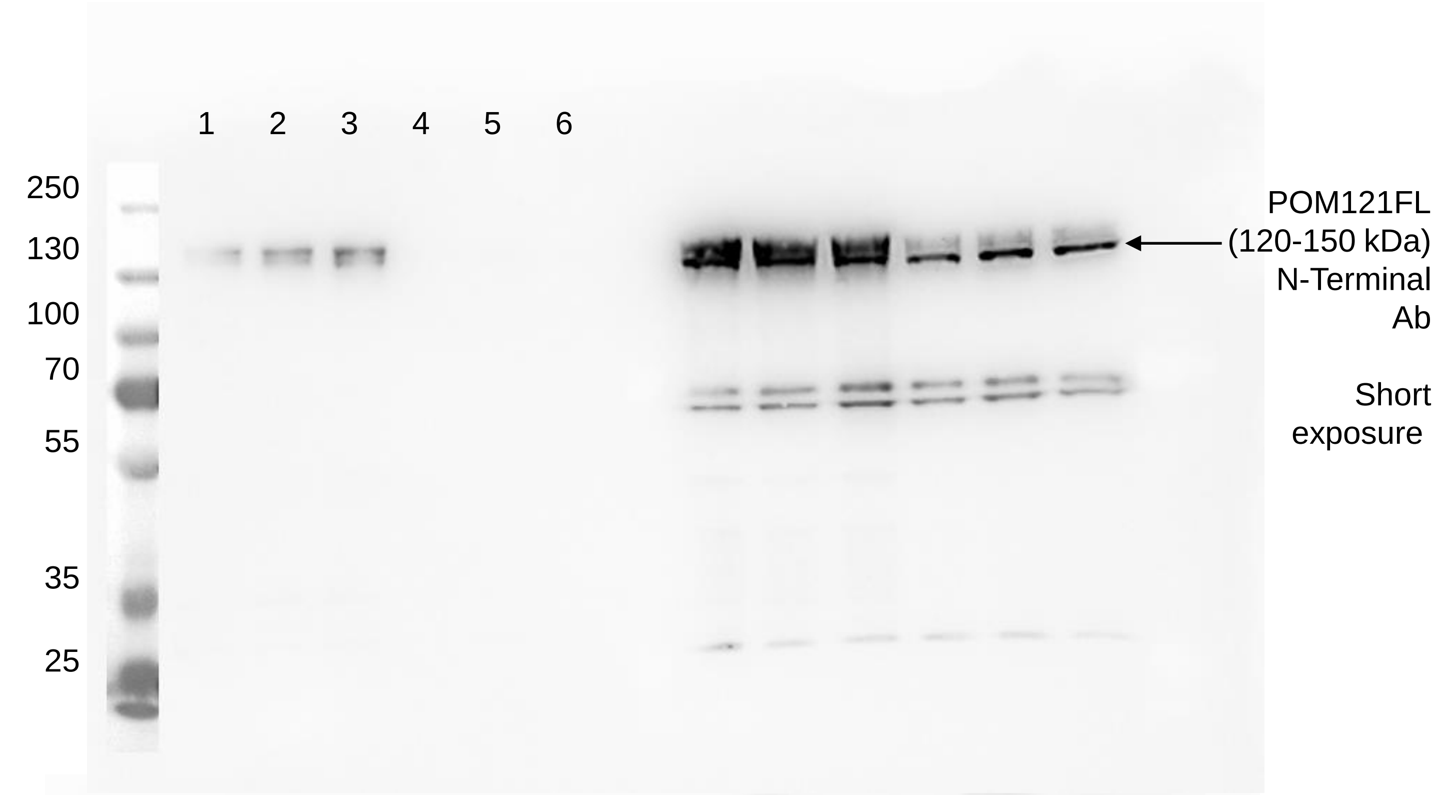

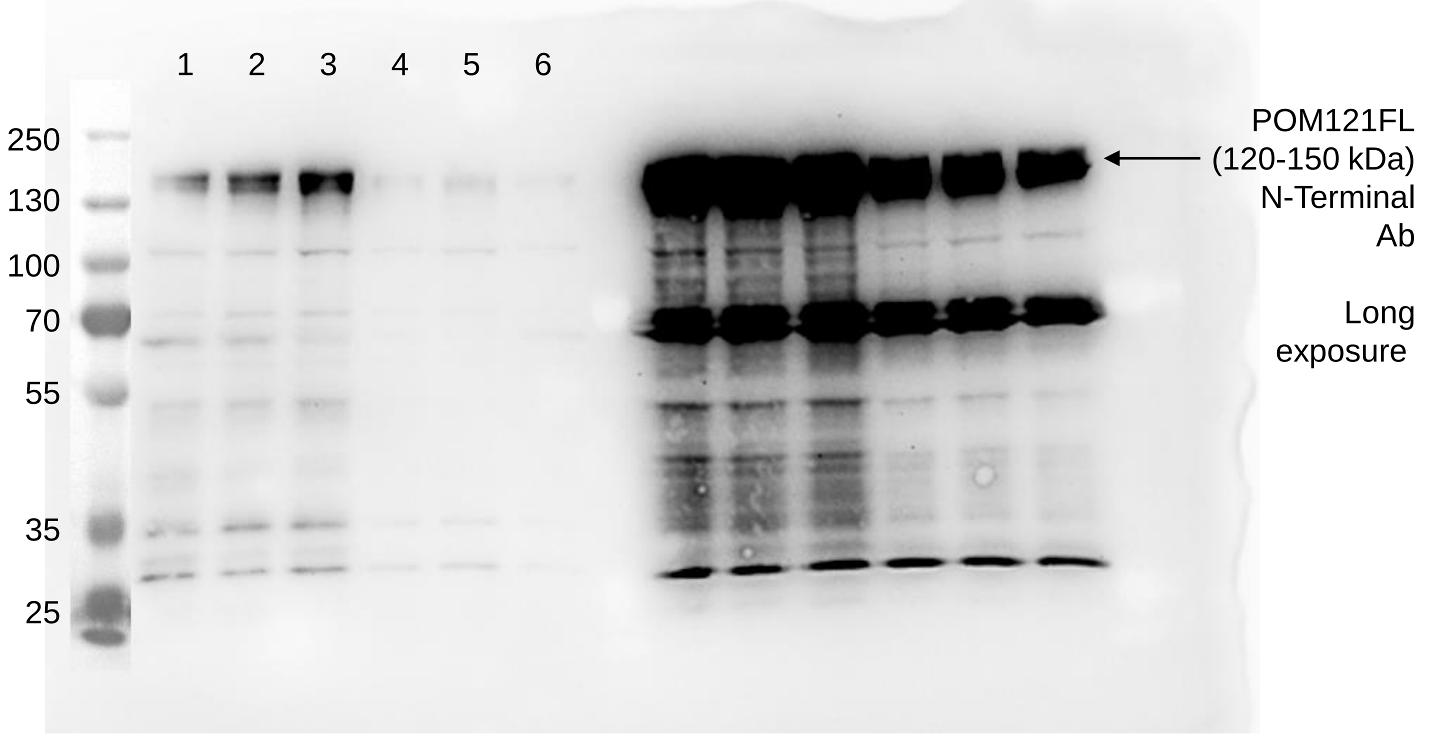

HT29: siRNA
1=Control+DMSO
2=Control+Rosi (1 µM)
3=Control+Rosi (10 µM)
4=POM121+DMSO
5=POM121+Rosi (1 µM)
6=POM121+Rosi (10 µM)

S16 HT29 cont.

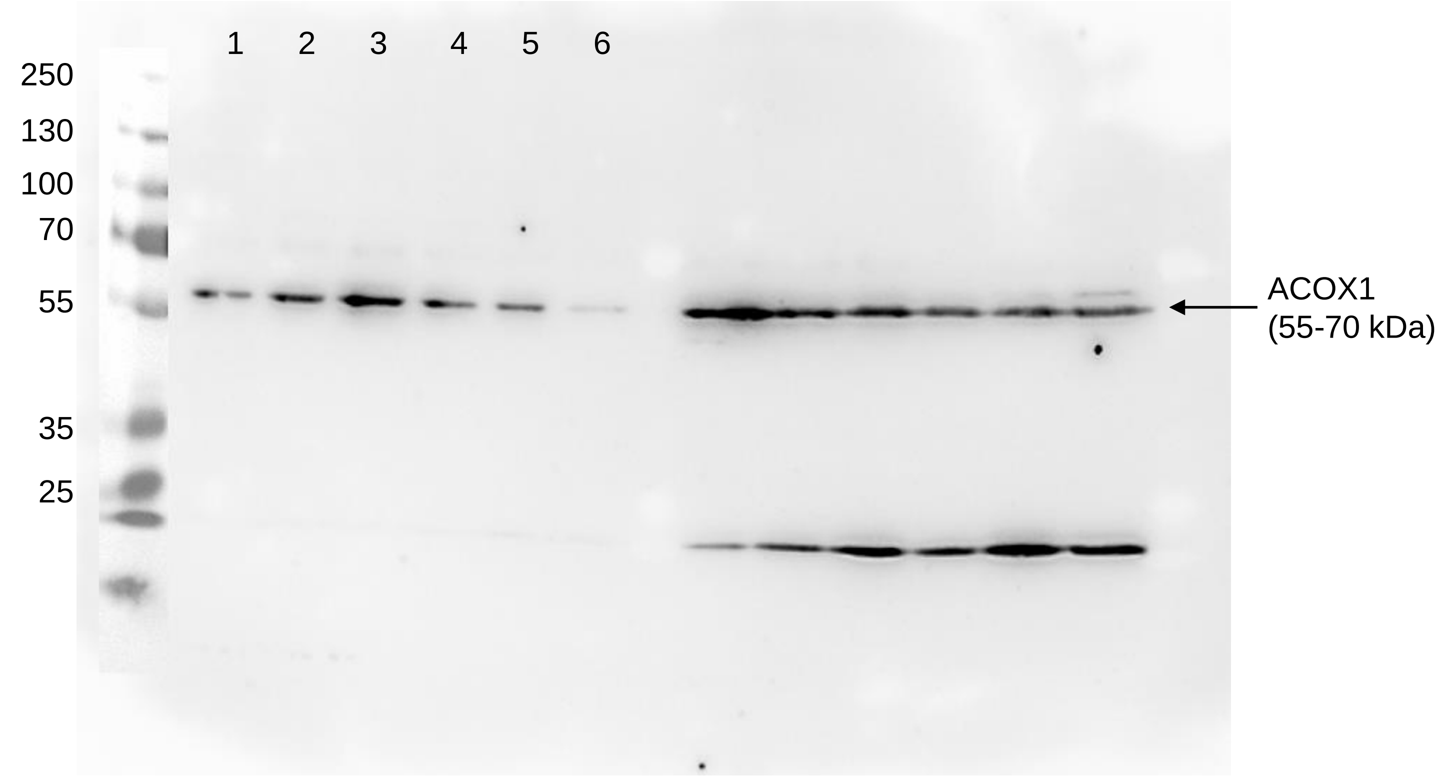

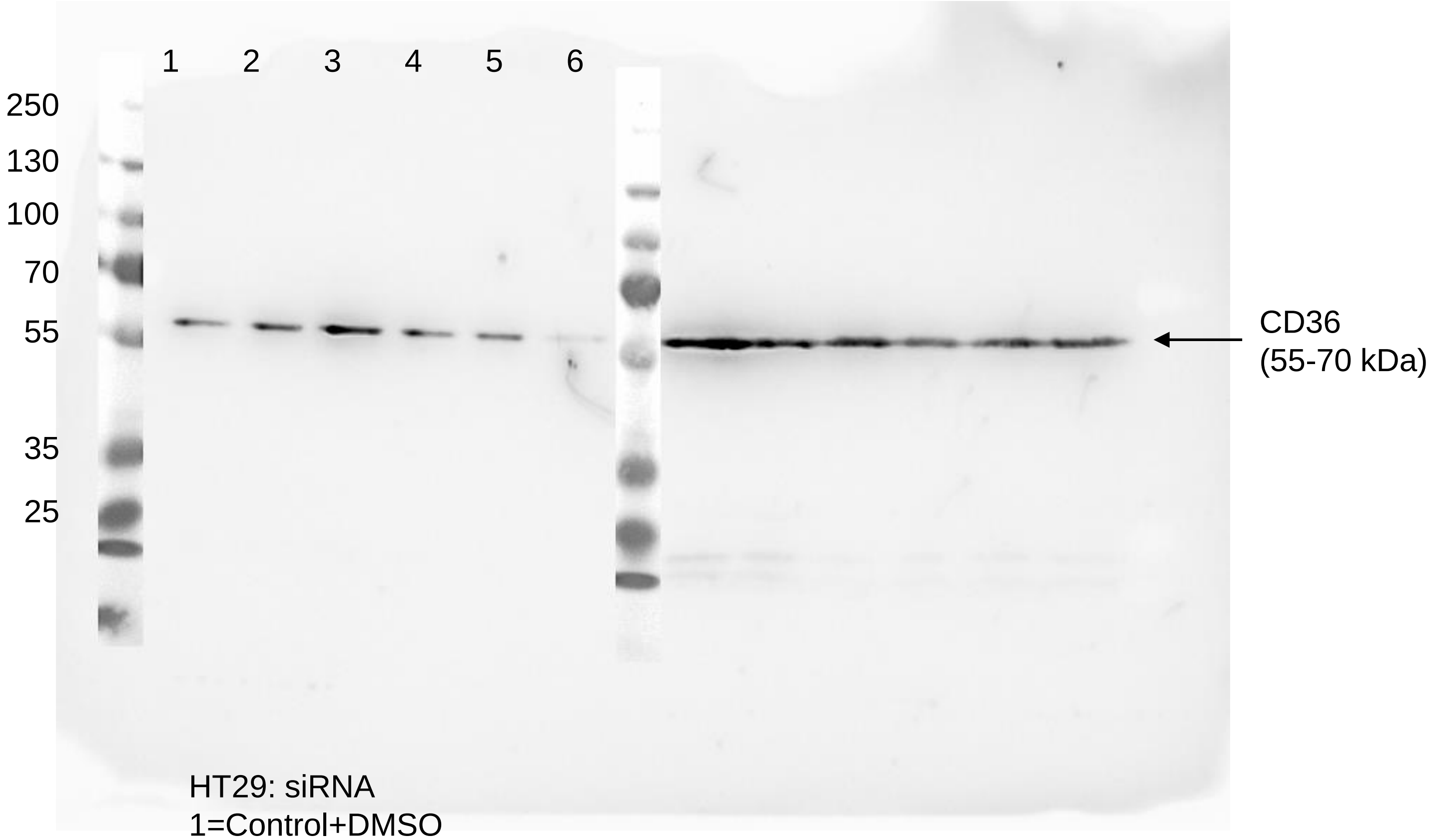

HT29: siRNA
1=Control+DMSO
2=Control+Rosi (1 µM)
3=Control+Rosi (10 µM)
4=POM121+DMSO
5=POM121+Rosi (1 µM)
6=POM121+Rosi (10 µM)

S16 HT29 cont.

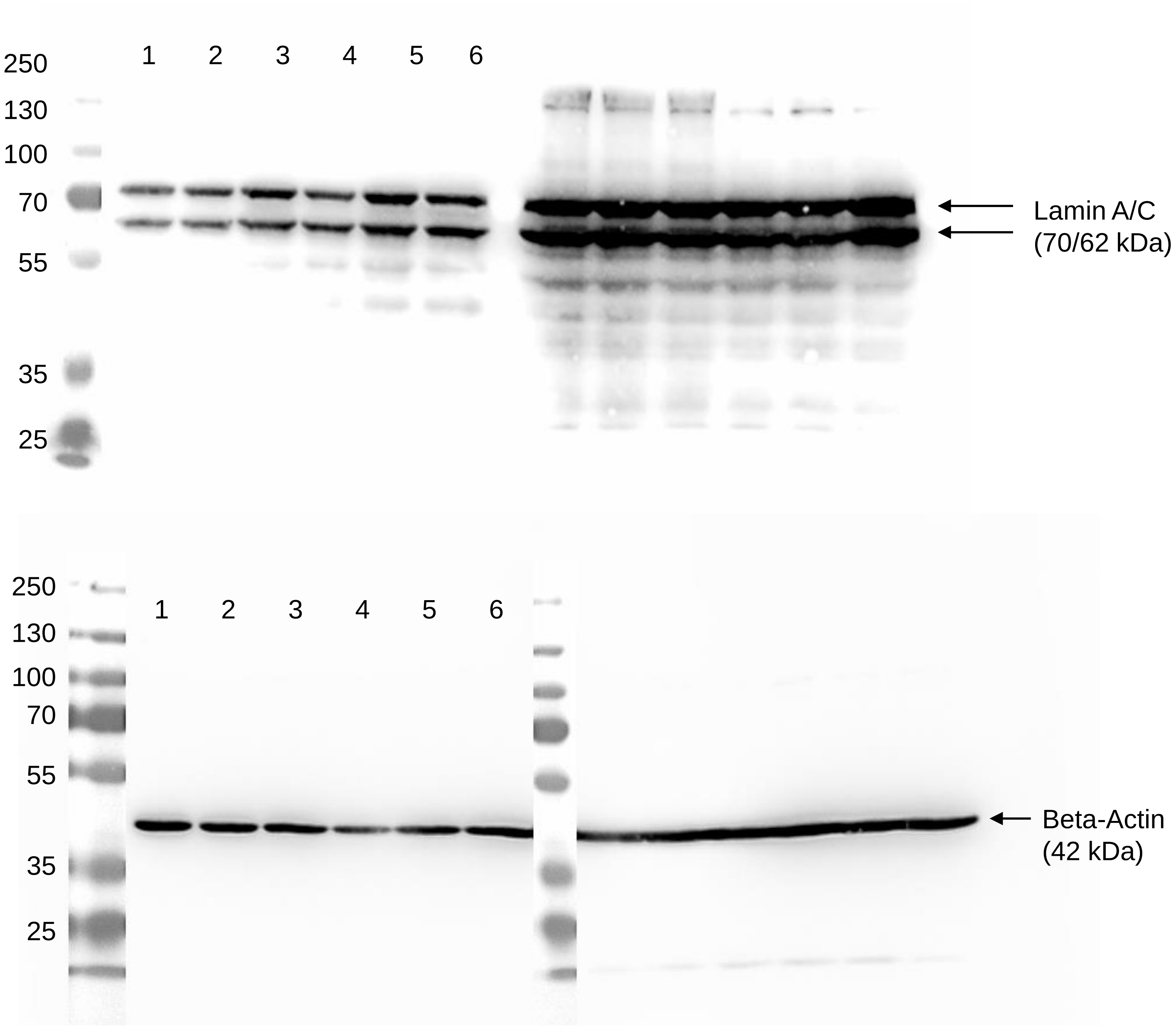

HT29: siRNA
1=Control+DMSO
2=Control+Rosi (1 µM)
3=Control+Rosi (10 µM)
4=POM121+DMSO
5=POM121+Rosi (1 µM)
6=POM121+Rosi (10 µM)
